# Customizable and stable multilocus chromosomal integration: a novel glucose-dependent selection system in *Aureobasidium* spp.

**DOI:** 10.1186/s13068-024-02531-3

**Published:** 2024-06-17

**Authors:** Shuo Zhang, Tao Ma, Fu-Hui Zheng, Muhammad Aslam, Yu-Jie Wang, Zhen-Ming Chi, Guang-Lei Liu

**Affiliations:** 1 MOE Key Laboratory of Evolution and Marine Biodiversity, College of Marine Life Sciences, Ocean University of China, Yushan Road, No. 5, Qingdao, 266003 Shandong China; 2 Laboratory for Marine Biology and Biotechnology, Qingdao Marine Science and Technology Center, No.1 Wenhai Road, Qingdao, 266237 China; 3 Faculty of Basic Sciences, Bolan University of Medical and Health Sciences, Quetta, 87600 Pakistan

**Keywords:** Non-conventional yeasts, Phosphofructokinase, Glucose-deficiency, Chromosomal integration, Selection system

## Abstract

**Background:**

Non-conventional yeasts hold significant potential as biorefinery cell factories for microbial bioproduction. Currently, gene editing systems used for these yeasts rely on antibiotic and auxotrophic selection mechanisms. However, the drawbacks of antibiotics, including high costs, environmental concerns, and the dissemination of resistance genes, make them unsuitable for large-scale industrial fermentation. For auxotrophic selection system, the engineered strains harboring auxotrophic marker genes are typically supplemented with complex nutrient-rich components instead of precisely defined synthetic media in large-scale industrial fermentations, thus lack selection pressure to ensure the stability of heterologous metabolic pathways. Therefore, it is a critical to explore alternative selection systems that can be adapted for large-scale industrial fermentation.

**Results:**

Here, a novel glucose-dependent selection system was developed in a high pullulan-producing non-conventional strain *A. melanogenum* P16. The system comprised a glucose-deficient chassis cell Δ*pfk* obtained through the knockout of the phosphofructokinase gene (*PFK*) and a series of chromosomal integration plasmids carrying a selection marker *PFK* controlled by different strength promoters. Utilizing the green fluorescent protein gene (*GFP*) as a reporter gene, this system achieved a 100% positive rate of transformation, and the chromosomal integration numbers of *GFP* showed an inverse relationship with promoter strength, with a customizable copy number ranging from 2 to 54. More importantly, the chromosomal integration numbers of target genes remained stable during successive inoculation and fermentation process, facilitated simply by using glucose as a cost-effective and environmental-friendly selectable molecule to maintain a constant and rigorous screening pressure. Moreover, this glucose-dependent selection system exhibited no significant effect on cell growth and product synthesis, and the glucose-deficient related selectable marker *PFK* has universal application potential in non-conventional yeasts.

**Conclusion:**

Here, we have developed a novel glucose-dependent selection system to achieve customizable and stable multilocus chromosomal integration of target genes. Therefore, this study presents a promising new tool for genetic manipulation and strain enhancement in non-conventional yeasts, particularly tailored for industrial fermentation applications.

**Supplementary Information:**

The online version contains supplementary material available at 10.1186/s13068-024-02531-3.

## Background

Non-conventional yeasts represent a diverse group of yeasts beyond well-established model organisms such as *Saccharomyces cerevisiae*, *Candida albicans*, and *Schizosaccharomyces pombe* [1]. They hold significant potential as biorefinery cell factories for microbial bioproduction owing to their inherent capacity to utilize a wide range of carbon sources and produce desired chemicals and proteins [2]. Additionally, non-conventional yeasts exhibit high tolerance to various stressful environments in biological processes, including osmotolerance, thermotolerance, and acid resistance [3]. However, the application of advanced metabolic engineering efforts in non-conventional yeasts remains limited due to the scarcity of well-characterized synthetic biology components and optimized genome engineering tools, impeding further progress in this field.

*Aureobasidium* spp., belonging to the subphylum Pezizomycotina within the class Dothideomycetes, are polyextremotolerant, melanin-producing, and dimorphism non-conventional yeasts with high biotechnological and biological importance [4]. They demonstrate a high adaptability to complex and varied environments, leading to their wide ecological distribution in marine, soil, beehive, polar marine sediments, desert, limestone, and mangrove ecosystems [5]. Moreover, *Aureobasidium* spp. possess the ability to synthesize various economically valuable primary and secondary metabolites such as fumaric acid [6], polymalate [7, 8], pullulan [9], intracellular lipids [10], liamocins [11], siderophore [12], Aureobasidin A [13], β-glycan [5], etc. Altogether, *Aureobasidium* spp. have garnered significant attention as promising eukaryotic hosts for industrial microbial bioproduction.

Genome editing plays a crucial role in enhancing the performance of non-conventional yeasts as cell factories by precisely engineering metabolic pathways to redirect carbon flow towards specific products [2]. Currently, the majority of metabolic engineering endeavors in *Aureobasidium* spp. rely on a Cre/LoxP site-specific recombination system, which allows for sequential gene deletion and expression, and has proven successful in investigating biosynthesis and regulation mechanism and constructing new cell factories [14]. However, it is noteworthy that the selectable markers of this system are limited to a few antibiotic resistance genes towards nourseothricin, hygromycin, and bleomycin [6, 11, 14]. Consequently, the maintenance of heterologous genes in engineered strains relies on the presence of antibiotics. However, the high cost and serious environmental issues associated with problems make them unsuitable for use in large-scale industrial fermentation, ultimately leading to a decline in the performance of engineered strains. Moreover, the excessive utilization of antibiotic resistance genes in metabolic engineering carries substantial risks, including their wide dissemination across multiple microbial taxa and their increasing accumulation in environments, thereby posing significant threats to public health and food safety [15, 16]. Therefore, the development of non-antibiotic selectable markers in *Aureobasidium* spp. is essential.

Auxotrophic marker genes, which do not rely on antibiotics, are extensively employed in genetic manipulation for microbial cell factory construction. In the case of non-conventional yeasts, a variety of auxotrophic strains have been developed as chassis cells, including those with amino acids, nucleotides, and vitamin auxotrophs [17–19]. These strains require specific nutritional formula that can be provided in defined synthetic media, facilitating control over growth and metabolism, and simplifying genetic manipulation. Moreover, due to their limited survival ability in the natural environment, they pose a lower risk of biological contamination [20]. However, the growth and product biosynthesis of auxotrophic strains are inherently constrained by the insufficient supplementation of essential growth factors such as amino acids and nucleotides [21]. This limitation becomes particularly significant in industrial fermentation, where there is a desire to streamline the process and reduce costs. In large-scale industrial fermentations, nutritional complementation of auxotrophs is typically accomplished using complex medium components that are rich in the required growth factors, rather than using precisely defined synthetic media [3, 6, 11]. In such cases, engineered strains harboring auxotrophic marker genes are not subject to selection pressure to maintain the stability of heterologous metabolic pathways. Consequently, it becomes crucial to explore novel selection systems that can be adapted for large-scale industrial fermentations.

Glucose, a simple sugar molecule, plays a crucial role as both an energy source and a carbon building block for cellular processes. It is widely recognized as the preferred carbon source for the majority of microorganisms in natural environments and is extensively utilized as the primary carbon source in industrial fermentation processes. Therefore, exploiting glucose deficiency as a selectable marker offers several advantages, including its low cost, widespread availability, robust screening capabilities, and the ability to exert continuous selection pressure during industrial fermentation. However, these possibilities remain to be explored.

In this study, we developed a novel glucose-dependent selection system in the high pullulan-producing strain *A. melanogenum* P16. This system involved generating of a glucose-deficient chassis cell obtained through the knockout of the phosphofructokinase gene (*PFK*). Additionally, we constructed a series of chromosomal integration plasmids carrying the selection marker *PFK*, controlled by various promoters. To assess the impact of different promoter strength of *PFK* on transformation efficiency, as well as on the number and stability of chromosomal integrations of target genes, we employed the green fluorescent protein gene (*GFP*) as a reporter gene. The results of our study demonstrate the feasibility of the glucose-dependent selection system in *Aureobasidium* spp., specifically enabling customizable and stable multilocus chromosomal integration of target genes under typical fermentation conditions. This advancement presents a promising new tool for genetic manipulation and strain improvement in non-conventional yeasts.

## Results and discussion

### The phosphofructokinase gene can serve as a glucose-deficient related selectable marker

Glucose plays a fundamental role in the metabolism of microorganisms and serves as the primary carbon source for industrial fermentation processes. Glycolysis initiates glucose metabolism by breaking down one glucose molecule into two pyruvate molecules while providing energy for cell growth. The phosphofructokinase enzyme (pfk) catalyzes the ATP-dependent phosphorylation of fructose 6-phosphate to form fructose 1,6-bisphosphate and ADP, representing a critical rate-limiting step in glycolysis [22]. Disruption of two copies of the *PFK* gene in *S. cerevisiae* abolished growth on glucose [23], indicating that *PFK* can serve as a viable glucose-deficient related selectable marker.

**Fig. 1:**
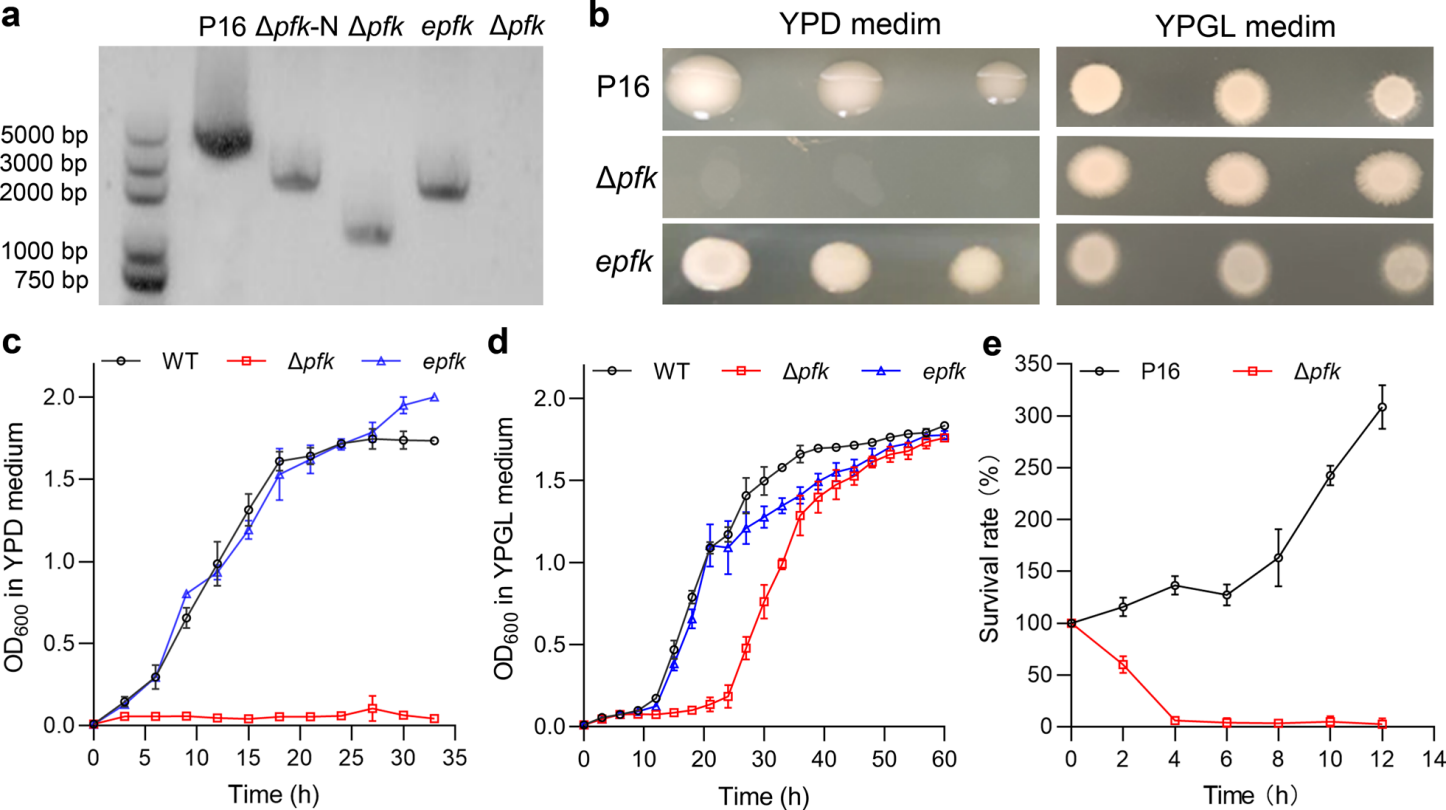
Growth phenotypes and growth curve of *A. melanogenum* P16, Δ*pfk* (P16 with *PFK* knocked out), *epfk* (Δ*pfk* with *PFK* complemented). **a** The genotypes verified by genomic PCR revealed the following: the fragment of the *PFK* knockout cassette was detectable in Δ*pfk*-N using the primes PFK-5F/PFK-3R, the fragment of the *PFK* knockout cassette without nourseothricin resistance gene was detectable in Δ*pfk* using the primes PFK-5F/PFK-3R, and the fragment of the *PFK* expressed cassette was detectable in *epfk* using the primes PFK-F/PFK-R. **b** The growth phenotype of P16, Δ*pfk*, and *epfk* on YPD (glucose) and YPGL (glycerol and lactate) media. **c** Time course of cell growth of P16, Δ*pfk*, and *epfk* in YPD medium within 33 h. **d** Time course of cell growth of P16, Δ*pfk*, and *epfk* in YPGL medium within 60 h. **e** The survival rate analysis of P16 and Δ*pfk* in YPD medium. Data are given as means ± SD, *n* = 3

In this study, we targeted the *PFK* gene in *A. melanogenum* P16, a remarkable non-conventional yeast known for its excellent pullulan-producing capability [24]. Unlike *S. cerevisiae*, *A. melanogenum* P16 possessed a single copy of *PFK* gene. After disrupting the *PFK* gene in *A. melanogenum* P16, the obtained mutant Δ*pfk-*N was further to eliminate nourseothricin resistance gene by transforming a plasmid of pAMCRE-1 with Cre recombinase gene, resulting in the final mutant Δ*pfk* (Table. S1) All the strains were confirmed by genomic PCR (Fig. 1a). As shown in Fig. 1b, the *PFK* mutant Δ*pfk* showed minimal growth impairment when utilizing glycerol and lactate as carbon sources compared to the wild-type strain P16. However, Δ*pfk* exhibited complete growth impairment in glucose (Fig. 1b), indicating the critical role of *PFK* in glucose metabolism and its significance as a stringent selectable marker. Growth curves further demonstrated that Δ*pfk* was unable to utilize glucose but exhibited similar growth levels in glycerol and lactate after 36 h, albeit with an initial growth delay, compared to P16 (Fig. 1c-d). Furthermore, this growth deficiency on glucose of Δ*pfk* was restored by the complementation of *PFK* in the strain *epfk* (Table S1 Fig. 1c). These results highlight the complete switch in growth phenotype on glucose in *Aureobasidium* spp. achieved through *PFK* knockout and complementation, thereby establishing *PFK* as a selectable marker associated with glucose deficiency and enabling the development of a novel glucose-dependent selection system.

To ensure the maintenance of all engineered strains harboring exogenous genes during fermentation processes, it is important to assess the impact of selectable marker gene loss on cell survival during fermentation. To simulate this scenario, Δ*pfk* cells pre-cultured on glycerol and lactate were transferred to a glucose medium, and their survival rate was analyzed. As shown in Fig. 1e, the analysis revealed a rapid decline in the survival rate of Δ*pfk* cells, with over 90% of yeast cells dying within 4 h. In contrast, the survival rate of P16 yeast cells continued to increase. These findings highlight the timely responsiveness and sensitivity of the glucose-dependent selection system, as the loss of the *PFK* gene results in the rapid death of yeast cells.

In addition, it is important to note that in this system, glucose, a readily available and cost-effective carbon source, can be utilized as a reliable and environmental-friendly selectable molecule, providing continuous selectable pressure in industrial fermentation. On the contrary, the high cost of antibiotics, particularly antifungal antibiotic, presents a significant challenge to their application in industrial production. For example, hygromycin B is priced at $104.21 per gram, while the cost of nourseothricin sulfate and bleomycin sulfate are $1528.53 and $1444.5 per gram, respectively. Furthermore, proper treatment of antibiotic fermentation waste is necessary to prevent irreversible ecological damage, incurring a significant economic cost of approximately $300 per ton [25]. The ecological risk of antibiotic resistance genes is another concern in the disposal of fermentation waste. Antibiotic resistance genes have the potential to transfer to humans through the food chain, posing a threat to human health. Reports indicate that the number of deaths caused by antibiotic-resistant infections worldwide is projected to rise from 700,000 in 2014 to a staggering 10 million annually by 2050, with the cumulative cost in terms of reduced healthcare and productivity reaching up to $100 trillion [26]. In contrast, the phosphofructokinase gene (*PFK*), being a widely distributed gene in organisms, does not pose a risk of gene contamination through horizontal gene transfer, making it a safe and environmentally friendly selectable marker.

Based on the above results, the *PFK* gene was chosen as the selectable marker for glucose-dependent selection system, and the strain Δ*pfk* was used as the chassis cell with glucose deficiency.

### Screening promoters of varying strength for the construction of the glucose-dependent selection system

**Fig. 2:**
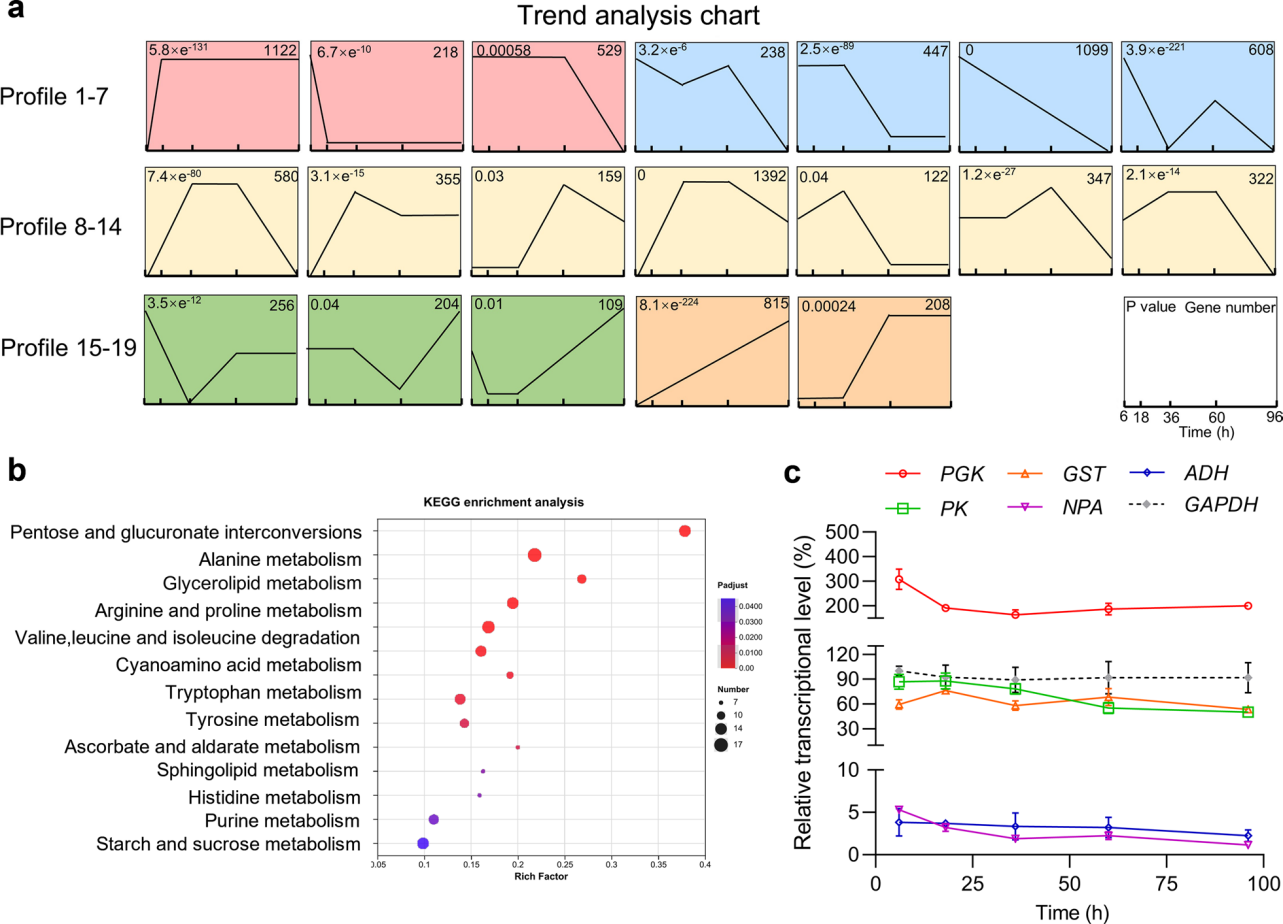
Transcriptome analysis of P16 and RT-qPCR analysis of genes. **a** Nineteen model profiles recognized by STEM, the number in the upper left-hand corner of the rectangle was the significance level *P*-value value, and the number in the upper right-hand corner was the number of genes. **b** KEGG pathway enrichment analysis of 1122 stably expressed genes. The horizontal axis represents the gene ratio, while the vertical axis represents the enriched pathway name. The color scale indicates different thresholds of the *P*-value, and the size of the dot indicates the number of genes corresponding to each pathway. **c** The relative transcriptional levels of *PGK*, *GST*, *ADH*, *PK*, *NPA* and *GAPDH* in P16. The values are the mean of three biological replicates, and error bars represent the standard deviations. Data are given as means ± SD, *n* = 3

**Fig. 3:**
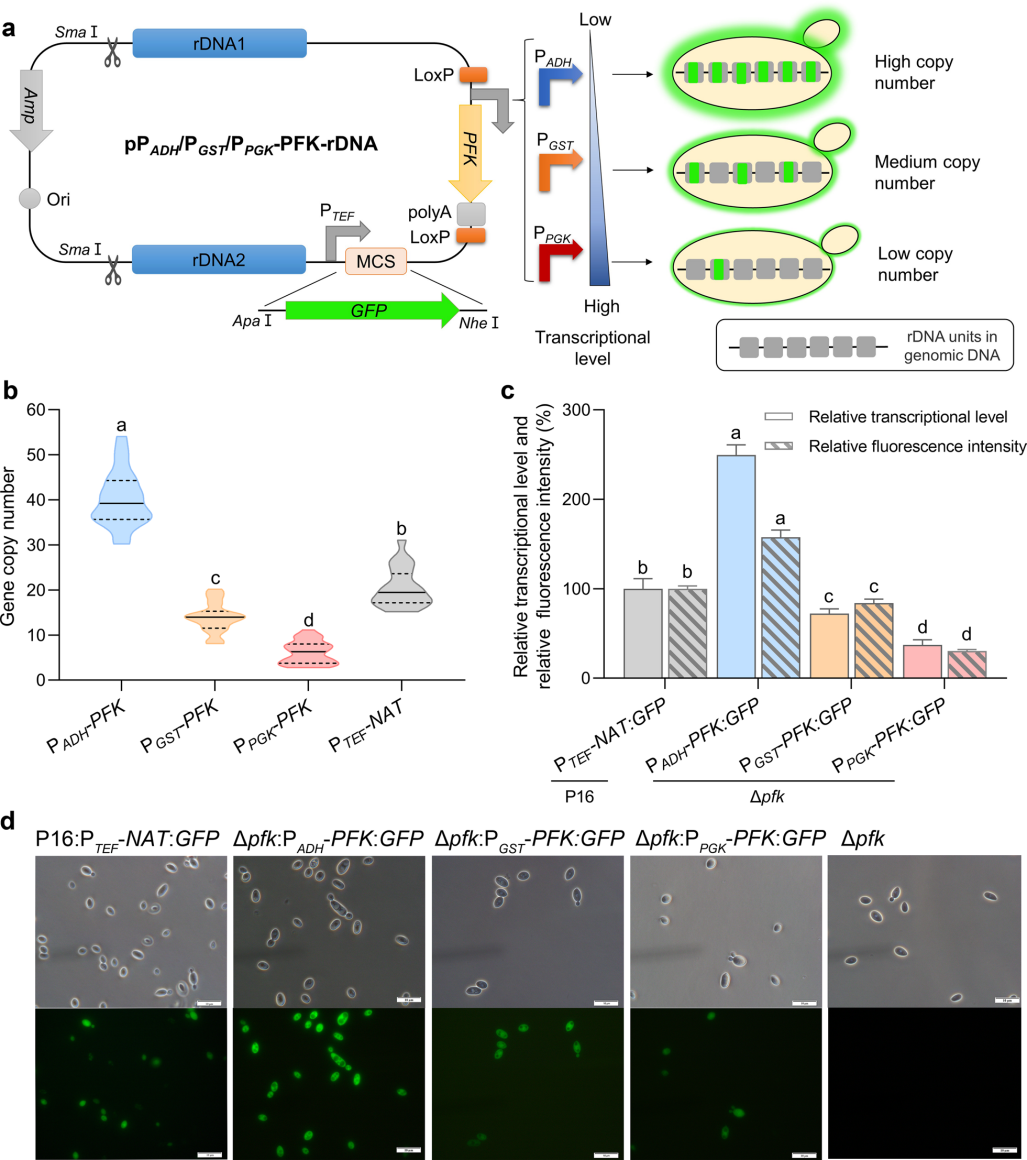
**a** Map of the pP_ADH_/P_*GST*_/P_*PGK*_-PFK-rDNA. **b** The gene copy number analysis of P16:P_*TEF*_-*NAT*:*GFP* and Δ*pfk* containing diverse promoters. **c** The relative fluorescence intensity and the relative transcriptional level analysis of Δ*pfk*:P_*ADH*_-*PFK*:*GFP,* Δ*pfk*:P_*GST*_-*PFK*:*GFP*, Δ*pfk*:P_*PGK*_-*PFK*:*GFP* and P16:P_*TEF*_-*NAT*:*GFP.* **d** Bright-field and fluorescence images of the Δ*pfk*:P_*ADH*_-*PFK*:*GFP,* Δ*pfk*:P_*GST*_-*PFK*:*GFP*, Δ*pfk*:P_*PGK*_-*PFK*:*GFP,* P16:P_*TEF*_-*NAT*:*GFP* and Δ*pfk*. Different letters in each group represent significant differences, Tukey HSD, *P* < 0.05. Data are given as means ± SD, *n* = 3

The strength of selection pressure is influenced by the expression levels of the selectable marker genes [27]. Promoters play a crucial role in regulating the initiation and intensity of transcription, thereby influencing gene expression at various levels [28]. Currently, the exploration of promoters in non-conventional yeasts is limited, mainly focusing on a few species such as *Yarrowia lipolytica* and *Pichia pastoris* [29–31]. To explore the specific promoters in *Aureobasidium* spp. that are adapted to the glucose-dependent selection system in this study, we conducted a temporal transcriptome analysis at five-time intervals during pullulan fermentation of *A. melanogenum* P16 (Fig. S1). After temporal variations analysis using the Short Time-series Expression Miner (STEM) program, a total of 17,915 genes were classified into 19 significantly different (*P* < 0.05) gene expression model profiles (Fig. 2a). To ensure stable and consistent selectable marker gene expression throughout the fermentation process, 1122 promoters of Profile 1 (Table S3) were selected as the candidate promoter database for further investigation. KEGG enrichment analysis revealed that these genes were enriched in glycometabolism, amino acid metabolism, and lipid metabolism (Fig. 2b), further indicating their constitutive expression patterns. From this set of candidates, six genes with distinct expression levels were selected, including genes encoding phosphoglycerate kinase (*PGK)*, pyruvate kinase (*PK)*, general substrate transporter (*GST)*, nascent polypeptide-associated complex α-subunit (*NPA)* and alcohol dehydrogenase (*ADH)*. The selection of these genes was corroborated by RT-qPCR. As shown in Fig. 3c, all tested genes demonstrated relatively stable transcription levels during the fermentation process. Furthermore, we categorized their transcription strength into three groups: *PGK* exhibited a transcription level approximately 200% of that of the reference gene *GAPDH*, while the analogs of *PK* and *GST*, and *NPA* and *ADH* were approximately 80% and 5%, respectively. Finally, we chose a diverse set of promoter modules based on these three groups, referred to P_*PGK*_, P_*GST*_ and P_*ADH*_, to drive the expression of the selectable marker gene *PFK* in our glucose-dependent selection system.

### The glucose-dependent selection system achieves a 100% positive rate of transformation

Based on the obtained promoter elements, a glucose-dependent selection system was constructed as shown in Fig. 3a. The new selectable marker gene *PFK* gene driven by the promoter of P_*PGK*_, P_*GST*_, or P_*ADH*_ replaced the nourseothricin selection element (P_*PGK*_-NAT) of the original expression plasmid pNAT-LoxP-rDNA for *Aureobasidium* spp. (Fig. S2), resulting in the plasmids of pP_*ADH*_-PFK-rDNA, pP_*GST*_-PFK-rDNA, or pP_*PGK*_-PFK-rDNA, respectively (Fig. 3a, Table S1). To assess the functionality of this glucose-dependent selection system, the reporter gene *GFP* was inserted into the multiple cloning sites (MCS) of these plasmids, resulting in the plasmids of pP_*ADH*_ / pP_*GST*_ / pP_*PGK*_ -PFK-rDNA-GFP (Table S1). Subsequently, these plasmids were linearized using *Sma*I and then transformed into the chassis cell Δ*pfk*. Transformation efficiency and positive rate were determined by screening the transformants on YPD plates and verifying them through genomic PCR. As a control, the original expression plasmid carrying *GFP* was transformed into the strain P16 and screened on YPD plates with nourseothricin.

As shown in Table 1, the positive rate of the transformants carrying *GFP* can reach 100% when using the glucose-dependent selection system with all three promoters. This result was consistent with the growth phenotype of Δ*pfk* on glucose, suggesting that the glucose-dependent selection system exhibited strong screening pressure when using glucose as sole carbon source, leading to a 100% positive rate. In contrast, the positive rate with the nourseothricin-dependent selection system was only 75% (Table 1), which might be attributed to variations in the ability of transformants to tolerate antibiotics. Hence, when utilizing a genetic engineering system that relies on antibiotic resistance, including our original nourseothricin-dependent selection system for *Aureobasidium* spp., an additional screening round should be conducted with higher antibiotic concentrations [9, 14]. In addition, the result in Table 1 revealed that the transformation efficiency of the glucose-dependent selection system with the promoters P_*PGK*_, P_*GST*_ and P_*ADH*_ were 18.43, 12.67, and 6.67 positive cfu per 1.0 μg of added DNA fragments, respectively, which were lower than that of the nourseothricin-dependent selection system. This means that the glucose-dependent selection system possesses a significantly higher positivity rate but sacrificing the transformation efficiency.

**Table 1:** Transformation efficiency and positive rate of the glucose-dependent selection system

Strains	cfu/μg DNA	Positive rate %
P16: P_*TEF*_-*NAT*:*GFP*	30.78 ± 4.53^a^	(75 ± 5.5)
Δ*pfk*: P_*ADH*_-*PFK*:*GFP*	6.67 ± 1.27^d^	100
Δ*pfk*: P_*GST*_- *PFK*:*GFP*	12.67 ± 2.88^c^	100
Δ*pfk*: P_*PGK*_-*PFK*:*GFP*	18.43 ± 3.46^b^	100

Data are given as means ± SD, *n* = 3. Each plate had 10^4^ competent cells. Different letters in each group represent significant differences, Tukey HSD, *P* < 0.05

Achieving high positive rate is an essential and desirable criterion for genetic engineering systems, because it can significantly reduce the burden of subsequent transformant screening and improve overall efficiency. In a previous study, using the CRISPR-Cas9 system for multigene expression in *Issatchenkia orientalis*, the integration efficiency was only 80% [32]. In another study, utilizing the amino acid nutrient auxotroph for genetic manipulation in *Pichia pastoris*, the deletion efficiency of individual genes ranged from 4 to 88% [33]. In contrast, the glucose-dependent selection system in this study, which could eliminate false positives through rigorous screening, is a promising tool for genetic manipulation.

### The strength of the *PFK* gene promoters can modulate the chromosomal integration numbers of target genes

To investigate the relationship between the chromosomal integration number of target genes and the strength of the *PFK* gene promoters, twenty transformants from each transformation with a specific *PFK* promoter were selected, and the copy numbers of the *GFP* gene were analyzed. As shown in Fig. 3b, strains containing P_*ADH*_-*PFK* exhibited a copy number range of 30–54 copies for the *GFP* gene, with a median of 39. For strains containing P_*GST*_- *PFK*, the copy number ranged from 8 to 20 copies, with a median of 14. In the case of *PFK* driven by the strongest promoter P_*PGK*_, the copy number only varied between 2 and11 copies, with a median of 6. These results indicate an inverse relationship between promoter strength and chromosomal integration numbers of target genes, while weaker promoter strength correlates with higher copy numbers. In addition, it is noteworthy that when using our original expression system with the nourseothricin selection element (P_*PGK*_-NAT), the *GFP* copy number exhibited range of 15–31 copies, which was significantly lower than that of the glucose-dependent selection system with P_*ADH*_-*PFK* (Fig. 3b). Generally, a high copy number integration is crucial for achieving high-level expression of target genes. In a previous study, a multicopy plasmid integration system based on *Leu2* auxotrophic selection was constructed in *Pichia pastoris*, and the strains containing 20 integrated copies of the vector were successfully isolated [34]. In comparison, the glucose-dependent selection system with P_*ADH*_-*PFK* in this study can achieve higher copy numbers (54 copies, Fig. 3b), suggesting the potential application of this system for efficient expression of target genes.

To further confirm this relationship, each strain with a specific *PFK* gene promoter, named Δ*pfk*:P_*ADH*_-*PFK*:*GFP*, Δ*pfk*:P_*GST*_-*PFK*:*GFP*, and Δ*pfk*:P_*PGK*_-*PFK*:*GFP* with *GFP* chromosomal integration numbers of 39, 14, and 6, respectively (Table. S1), was subjected to analysis of fluorescence intensity, *GFP* transcriptional level and fluorescence microscopy. The control strain transformed with the original expression plasmid carrying *GFP* and nourseothricin resistance gene, named P16:P_*TEF*_-*NAT*:*GFP* with a *GFP* chromosomal integration number of 19, was also included. As shown in Fig. 3c and d, the strain Δ*pfk*:P_*ADH*_-*PFK*:*GFP* exhibited the highest fluorescence intensity and *GFP* transcriptional level, followed by the strains Δ*pfk*:P_*GST*_-*PFK*:*GFP* and Δ*pfk*:P_*PGK*_-*PFK*:*GFP*. This sequential decrease in *GFP* expression was consistent with the trend of their *GFP* gene chromosomal integration numbers, further confirming that the strength of the *PFK* gene promoters were negatively correlated with the chromosomal integration numbers and expression levels of target genes. Therefore, based on the candidate promoter database in this study, the expression level of *PFK* can be finely tuned by selecting appropriate promoters, thereby realizing the customization of the chromosomal integration number and expression level of target genes in *Aureobasidium* spp.

The chromosomal integration of heterologous biosynthetic pathways is indispensable for constructing robust and high-performing cell factories. To achieve commercially viable production of target compounds, the expression levels of the metabolic genes must be fine-tuned to allow achieve a balanced metabolic flux, ensuring efficient conversion of substrates into the desired product [35]. Therefore, it is important to develop effective synthetic biology tools capable of customizing the gene chromosomal integration number. To date, the methods of customizing gene chromosomal integration number have primarily involved increasing screening pressure or modifying the sequence length of the selectable marker promoter [36, 37]. For instance, a modified version of post transformational vector amplification, known as Liquid PTVA, involves gradually increasing the concentration of antibiotics in the liquid medium to obtain strains with different copy numbers ranging from 1 to 18 [36]. However, this method still relies on the use of antibiotics, and overall time cost is 12 days. Another approach involves using the truncated *URA3* promoter (P_*URA*_) to regulate the expression of resistance genes in *S. cerevisiae*, resulting in the copy number of target gene within the range of 5 to 35 [37]. In this study, through utilizing P_*PGK*_, P_*GST*_ or P_*ADH*_ as the *PFK* promoter, the glucose-dependent selection system was able to achieve target gene chromosomal integration numbers ranging from 2 to 54, resulting in an 8.3-fold difference in expression levels (Fig. 3). Moreover, the candidate database consisting of 1122 promoters will provide additional opportunities to finely tune the expression levels of target genes within the system, facilitating the desired gene copy number range and providing the necessary range of expression levels for synthetic biology applications.

### The chromosomal integration numbers of target genes remain stable during successive inoculation and fermentation process

The rDNA gene, responsible for encoding the RNA components of ribosomes, is highly repetitive with typically 100–1000 copies. These gene are arranged in large stretches of tandem repeats, forming loci that are highly susceptible to copy loss due to intrachromatid homologous recombination between copies [38]. Moreover, since only a subset of rDNA copies is essential for normal cellular function, the silencing of unnecessary rDNA copies can result in DNA damage and copy number variation [38]. Previous studies have reported a significant decrease in the number of target genes integrated in rDNA after 48–72 h of incubation without screening pressure [39]. Therefore, ensuring the stability of the glucose-dependent selection system is an essential requirement for industrial fermentation application.

**Fig. 4:**
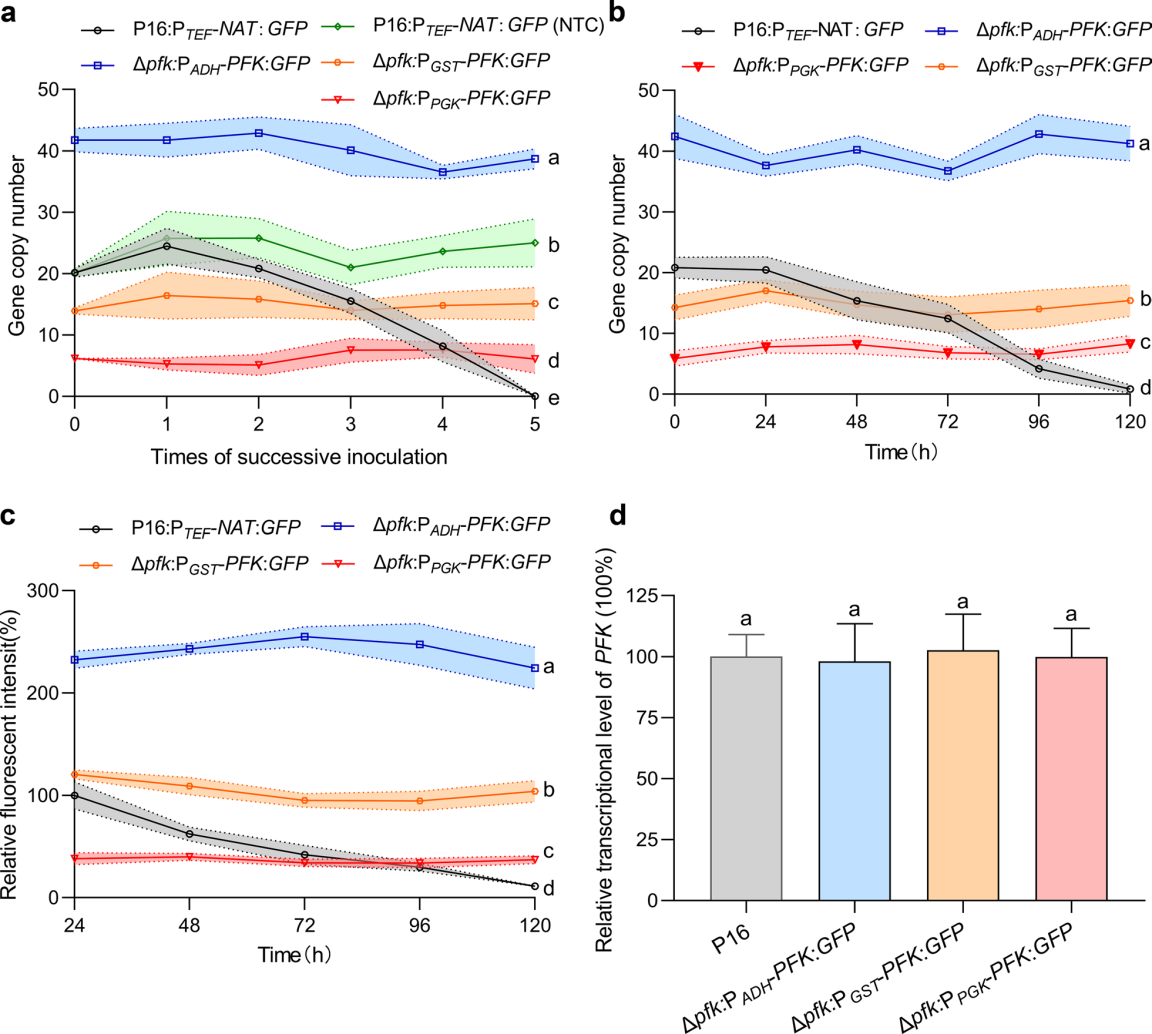
Stability of the system during successive inoculation and fermentation process. **a** The gene copy number analysis of *GFP* in Δ*pfk*:P_*ADH*_-*PFK*:*GFP,* Δ*pfk*:P_*GST*_-*PFK*:*GFP*, Δ*pfk*:P_*PGK*_-*PFK*:*GFP*, and P16:P_*TEF*_-*NAT*:*GFP* during five successive inoculations. **b** The gene copy number analysis of *GFP* in Δ*pfk*:P_*ADH*_-*PFK*:*GFP,* Δ*pfk*:P_*GST*_-*PFK*:*GFP*, Δ*pfk*:P_*PGK*_-*PFK*:*GFP*, and P16:P_*TEF*_-*NAT*:*GFP* during fermentation process. **c** The relative fluorescence intensity analysis of Δ*pfk*:P_*ADH*_-*PFK*:*GFP,* Δ*pfk*:P_*GST*_-*PFK*:*GFP*, Δ*pfk*:P_*PGK*_-*PFK*:*GFP*, and P16:P_*TEF*_-*NAT*:*GFP* during fermentation. **d** The *PFK* relative transcript levels of Δ*pfk*:P_*ADH*_-*PFK*:*GFP,* Δ*pfk*:P_*GST*_-*PFK*:*GFP*, Δ*pfk*:P_*PGK*_-*PFK*:*GFP*, and P16:P_*TEF*_-*NAT*:*GFP*. Different letters in each group represent significant differences, Tukey HSD, *P* < 0.05. Data are given as means ± SD, *n* = 3

Successive inoculation is a common practice in industrial fermentation processes, such as strain activation and preservation, preparation of seed liquid, and the amplification of multi-stage fermentation. To evaluate the passaging stability of target gene in glucose-dependent selection system, the strains Δ*pfk*:P_*ADH*_-*PFK*:*GFP*, Δ*pfk*:P_*GST*_-*PFK*:*GFP* and Δ*pfk*:P_*PGK*_-*PFK*:*GFP* (Table S1) were subjected to five successive inoculations in YPD medium. The strains P16:P_*TEF*_-*NAT*:*GFP* cultured in YPD medium with and without 100 μg/mL nourseothricin were used as controls. As indicated in Fig. 4a, the *GFP* copy numbers of all the strains constructed using glucose-dependent selection system showed excellent stability, which was consist with the observation about the strain P16:P_*TEF*_-*NAT*:*GFP* cultured with nourseothricin. In contrast, the *GFP* integrated into chromosomes in the strain P16:P_*TEF*_-*NAT*:*GFP* was almost lost after five successive inoculations without nourseothricin (Fig. 4a). These results suggest that using the glucose-dependent selection system for strains construction, maintaining stable chromosomal integration numbers of target genes during successive inoculation can be accomplished by simply cultivating the strains on the most commonly used medium containing glucose. Furthermore, this antibiotic-free system will simplify the process of successive inoculation reduce, the costs associated with antibiotics, and avoid complex post-treatment process of wastewater containing antibiotics, particularly in large-scale multi-stage fermentations.

In addition to the stability of successive inoculation, the consistent performance of fermentation throughout the fermentation process is a vital criterion for engineered strains utilized in industrial fermentation. As shown in Fig. 4b, the copy numbers of the *GFP* gene remained stable at 40–45 in Δ*pfk*:P_*ADH*_-*PFK*:*GFP*, 14–17 in Δ*pfk*:P_*GST*_-*PFK*:*GFP*, and 6–9 in Δ*pfk*:P_*PGK*_-*PFK*:*GFP* throughout the fermentation process. However, in the case of P16:P_*TEF*_-*NAT*:*GFP*, there was a gradual decrease, leading to complete loss at 120 h. These results were consistent with the relative fluorescence intensity that the relative fluorescence intensities of Δ*pfk*:P_*ADH*_-*PFK*:*GFP,* Δ*pfk*:P_*GST*_-*PFK*:*GFP*, and Δ*pfk*:P_*PGK*_-*PFK*:*GFP* remained stable throughout the fermentation process, while P16:P_*TEF*_*-NAT*:*GFP* continuously decreased (Fig. 4c). These results suggest that the glucose-dependent selection system was capable of maintaining stable copy numbers of target genes during fermentation. This may be attributed to the need for maintaining high transcriptional levels of *PFK*, as it plays a crucial role in glucose metabolism, as well as pullulan synthesis. As expected in Fig. 4d, though driven by different promoters with different strength, the expression of *PFK* in P16, Δ*pfk*:P_*ADH*_-*PFK*:*GFP,* Δ*pfk*:P_*GST*_-*PFK*:*GFP*, and Δ*pfk*:P_*PGK*_-*PFK*:*GFP* exhibited comparable transcriptional levels. This highlights the advantage of using key genes involved in glucose metabolism as selection markers, as the significant metabolic flux requirements render strains devoid of copy numbers for these genes less competitive during the fermentation process.

Industrial fermentation aims to achieve efficient and cost-effective production processes. In order to fulfill nutritional requirements in fermentation media, complex nitrogen sources like yeast extract, as well as inexpensive crude nitrogen sources such as corn steep liquor, soybean meal, and cottonseed flour, are commonly utilized [40]. However, the various nutritional components of these sources in fermentation media, including nucleic acids, amino acids, vitamins, and other cofactors, do not provide effective selection pressure on the engineered strains obtained through auxotrophic selection, leading a risk of destabilizing of chromosomal integration numbers of target genes during fermentation process. In contrast, based on the system constructed in this study, glucose, one of the most used carbon sources in industrial fermentation, can maintain a constant and rigorous screening pressure during successive inoculation and fermentation process (Fig. 4). Therefore, the glucose-dependent selection system is a promising tool to construct engineered strains for industrial fermentation.

### Glucose-dependent selection system has no significant influence on pullulan fermentation

**Fig. 5:**
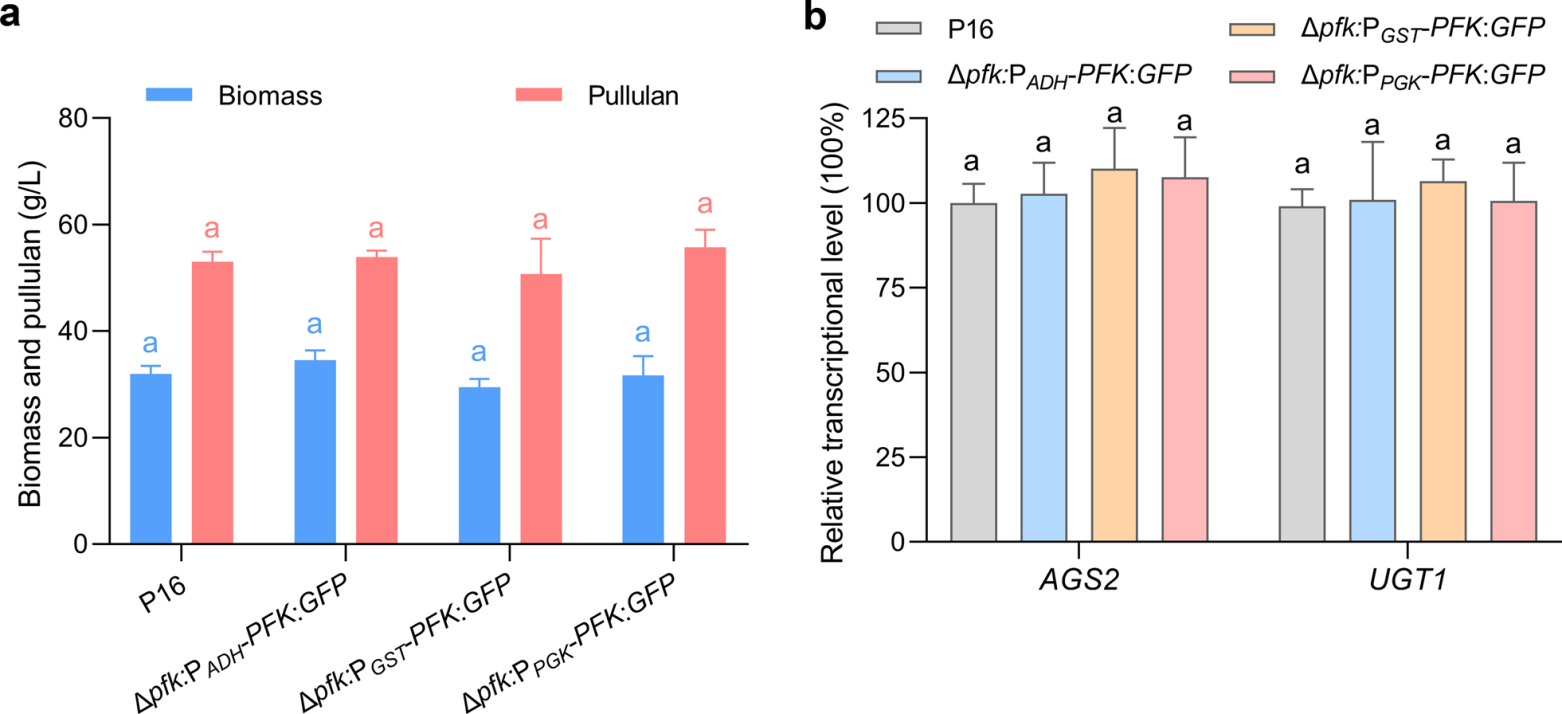
**a** Pullulan yield and biomass of Δ*pfk*:P_*ADH*_-*PFK*:*GFP,* Δ*pfk*:P_*GST*_-*PFK*:*GFP*, Δ*pfk*:P_*PGK*_-*PFK*:*GFP*, and P16 was determinate after fermentation. **b** The relative transcript levels of *AGS2* and *UGT1* in Δ*pfk*:P_*ADH*_-*PFK*:*GFP,* Δ*pfk*:P_*GST*_-*PFK*:*GFP*, Δ*pfk*:P_*PGK*_-*PFK*:*GFP*, and P16. Different letters in each group represent significant differences, Tukey HSD, *P* < 0.05. Data are given as means ± SD, *n* = 3

As mentioned above, the glucose-dependent selection system could successfully restore the glucose metabolism in strain Δ*pfk* (Fig. 1b and c). However, considering that pullulan synthesis necessitates a significant provision of glucose precursors and energy [41], the pullulan production of Δ*pfk*:P_*ADH*_-*PFK*:*GFP,* Δ*pfk*:P_*GST*_-*PFK*:*GFP*, and Δ*pfk*:P_*PGK*_-*PFK*:*GFP* still required evaluation. As indicated in Fig. 5a, the pullulan titer and cell dry weight of these strains showed no significant differences compared to the wild-type strain P16. Furthermore, the two key genes involved in pullulan synthesis, including *AGS2* responsible for pullulan synthesis and *UGT1* responsible for UDP-glucose synthesis [41], showed comparable transcriptional levels among three engineered strains and P16 (Fig. 5b). These results indicate that the glucose-dependent selection system successfully satisfied the carbon flux required for cell growth and pullulan synthesis, which consistent with the observation that the expression of *PFK* in engineered strains showed comparable transcriptional levels to that of the wild-type P16 (Fig. 4d)*.* For auxotrophic selection system, it is reported that the most amino acid auxotrophs of *S. cerevisiae* showed reduced cell growth and fatty acid production when supplemented with commonly used concentrations of amino acids, and these observed side effects could be remedied by the application of higher supplement [21]. Nevertheless, employing elevated supplementation levels is economically impractical for industrial fermentation processes. In addition, a leucine-auxotroph *Y. lipolytica* strain was unable to restore the biomass equivalent to that of a prototrophic strain even after complementing the marker gene [42]. In contrast, the glucose-dependent selection system has no significant influence on growth and product biosynthesis of engineered strains, showcasing potential for application in industrial fermentation, especially in the fermentation polysaccharide, organic acids, and lipids that rely heavily on glucose.

### Universality of the *PFK* gene as a glucose-deficient related selectable marker

**Fig. 6:**
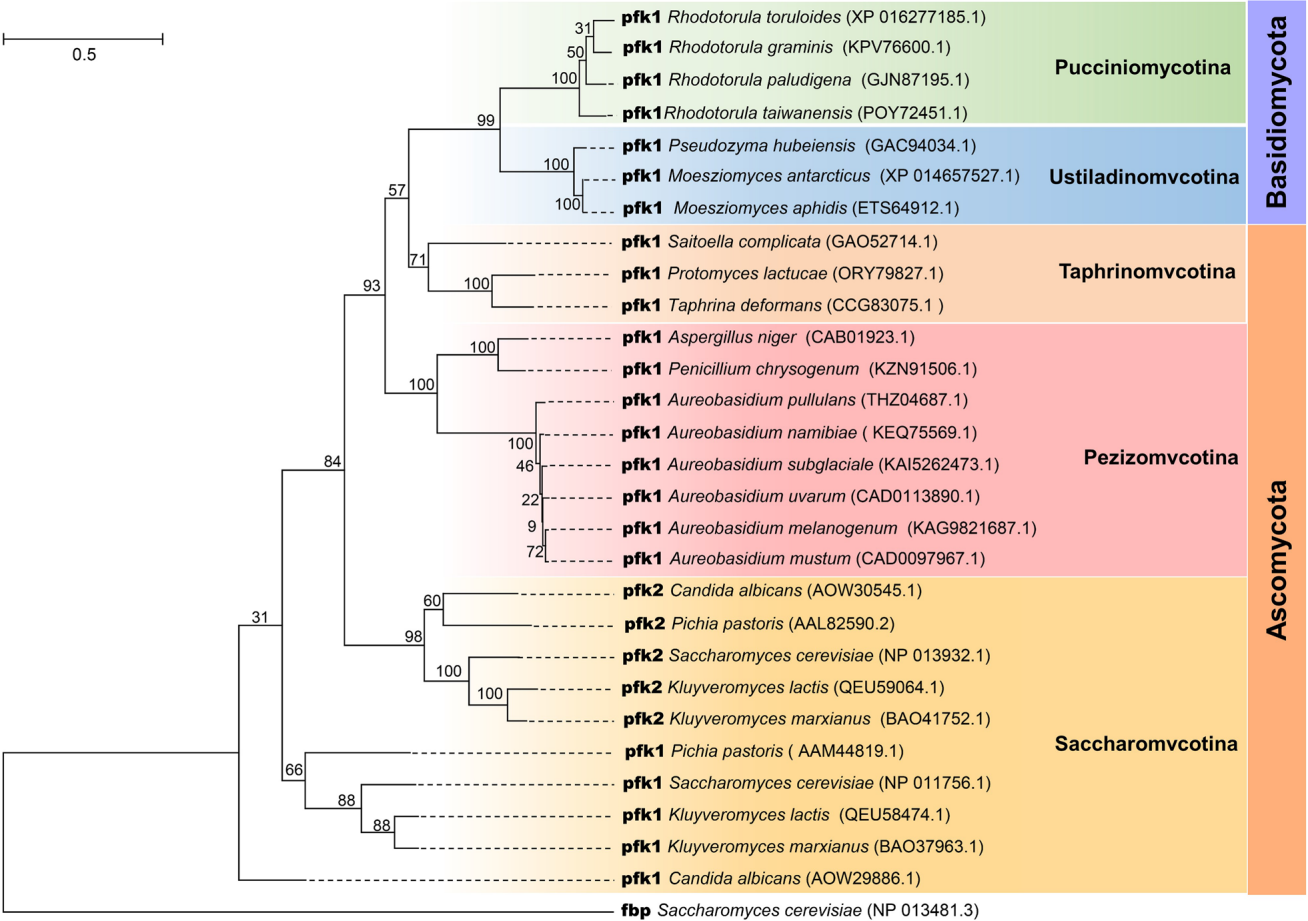
The phylogenetical tree of the pfk amino acid sequences from phylum basidiomycota and ascomycota, the amino acid sequence of fbp in S*. cerevisiae* was used as an outgroup

Based on the above results, the *PFK* gene, serving as a glucose-deficient related selectable marker show significantly advantages over antibiotics resistance genes and auxotrophic markers, including rigorous and rapid screening with 100% positive rate, stability across successive inoculations and fermentation processes, and no adverse effects on cell growth and metabolism. To provide references for utilizing this gene as a selection marker in other fungi, the phylogenetic tree of phosphofructokinase from the phyla of Basidiomycota and Ascomycota was constructed. As shown in Fig. 6, the fungi from subphyla Pucciniomycotina, Ustilaginomycotina, Taphrinomycotina, and Pezizomycotina only possess one phosphofructokinase (pfk1), while two phosphofructokinase isoforms (pfk1 and pfk2) were found in Saccharomycotina. Phosphofructokinase is a multisubunit enzyme that exists as a heterooctamers α4β4 in *S. cerevisiae*, where *PFK1* and *PFK2* encode the α-subunit and the β-subunit, respectively [22]. In strains solely *PFK1*, pfk consists of single subunits, forming a homotetramer or homooctamer [43]. These findings indicate that in strains harboring a single *PFK* gene, the Δ*pfk* chassis cell can be achieved by disrupting one gene. However, in strains containing two *PFK* genes, both genes need to be disrupted. As indicated in Fig. 6, pfk1 are conserved across various strains of *Aureobasidium* spp. Previous studies have demonstrated that these strains are capable of efficiently producing various high-value products from glucose. For instance, *A. melanogenum* TN3-1 can produce over 110 g/L of pullulan from 140 g/L of glucose [9], while *A. pullulans var. aubasidani* DH177 can accumulate 32.3 g/L fumaric acid from 120 g/L glucose [6]. Additionally, *A. melanogenum* 9-1 can produce 40 g/L of liamocins form 130 g/L glucose [11]. This suggests that that the system developed in this study can be widely applied within *Aureobasidium* spp. Furthermore, this system also has promising applications in several other non-conventional yeasts shown in Fig. 6 with a single *PFK* gene, which have the potential to serve as biorefinery cell factories utilizing glucose as a carbon resource. Notably, *Rhodotorula paludigena* P4R5 has demonstrated the ability to accumulate 23.5 g/L polyol esters of fatty acids from 140 g/L glucose [44]. *Moesziomyces aphidis* XM01 can accumulate 53.9% intercellular lipid from 80 g/L glucose [45].

In addition to glucose, fructose is also a common carbon source widespread in nature [40]. It is worth noting that phosphofructokinase is also a critical metabolic enzyme for fructose, because after the action of hexokinase, fructose 6-phosphate, generated from fructose, enters glycolysis through the same pathway as glucose [46]. As expected, the strain Δ*pfk* abolished growth in fructose (Fig. S3). Therefore, in addition to glucose, the *PFK* gene could also sever as a reliable growth-deficient related selectable marker in the fermentation system utilizing fructose as the carbon source, along with certain saccharides producing end-products of glucose and fructose, such as starch, cellulose, sucrose, molasses, and inulin.

## Conclusions

At present, there is a critical need to explore alternative selection systems that are not dependent on antibiotics and auxotrophs, but can be tailored for large-scale industrial fermentations. In this study, we developed a novel glucose-dependent selection system in the high pullulan-producing strain *A. melanogenum* P16. This system enabled a 100% positive rate of transformation and stable multilocus chromosomal integration of target genes. It is noteworthy that through utilizing P_*PGK*_, P_*GST*_ or P_*ADH*_ as the *PFK* promoter, the glucose-dependent selection system was able to achieve target gene chromosomal integration numbers ranging from 2 to 54, resulting in an 8.3-fold difference in expression levels. Moreover, the candidate promoter database established in this study allows for precise customization of chromosomal integration number of target genes, providing the necessary range of expression levels for synthetic biology applications. Not only that, in addition to *A. melanogenum*, the glucose-deficient related selectable marker *PFK* has universal application potential in other non-conventional yeasts. Therefore, this work paves a promising way for the development of a new genetic manipulation tool for non-conventional yeasts, particularly tailored for industrial fermentation applications.

## Materials and methods

### Strains, media and plasmids

Strains and plasmids used in this study are listed in Table S1. *A. melanogenum* P16 and *epfk* were cultured in YPD medium (20 g/L glucose, 10 g/L yeast extraction, and 20 g/L peptone), while Δ*pfk* and Δ*pfk-NAT* was cultured in YPGL medium (20 g/L glycerol, 20 g/L lactate, 10 g/L yeast extract, and 20 g/L peptone). The pullulan production broth was composed of 120 g/L glucose, 0.2 g/L yeast extract, 0.2 g/L (NH_4_)_2_SO_4_, 7 g/L KH_2_PO_4_, 2.5 g/L Na_2_HPO_4_·12H_2_O, 1.5 g/L MgSO_4_·7H_2_O, 0.02 g/L MnSO_4_, 0.02 g/L ZnSO_4_, 0.15 g/L FeCl_3_, and 0.15 g/L CaCl_2_ [24].

### Knockout and complementation of the *PFK* gene

For *PFK* gene knockout, the *PFK*-5′arm and the *PFK*-3′arm were amplified from the *A. melanogenum* P16 genome using the primers PFK-5F/PFK-5R and PFK-3F/PFK-3R, respectively (Table. S2). *PFK*-5’ arm and *PFK*-3′ arm were then digested with *BamH*I/*EcoR*I and *Sph*I/*Pst*I, respectively, and ligated into the vector fl4a-NAT-LoxP to obtain fl4a-NAT-LoxP-PFK (Table. S2). The linear DNA fragment was amplified from the fl4a-NAT-LoxP-PFK using the primers PFK-5F/PFK-3R (Table. S2), and transformed into the strain P16 according to a spheroplast transformation protocol [6]. The positive transformants were screened on the YPGL plates with 100 μg/mL nourseothricin and then were verified by PCR analysis using the primers PFK-5F/PFK-3R to obtain Δ*pfk*-N*.* Then, the plasmid pAMCRE-1 with self-replicating DNA sequence (ARS) and Cre recombinase gene (Table. S1) was used to remove the *NAT* gene in Δ*pfk-NAT* according to our previous method [6]. The final strain verified by PCR analysis using the primers PFK-5F/PFK-3R was named Δ*pfk.*

For *PFK* gene complementation, the *PFK* gene was amplified by PCR from *A. melanogenum* P16 genomic DNA using the primers PFK-F/PFK-R (Table. S2) and ligated into the *Pst*I/*Spe*I site of the vector pHPT-LoxP-rDNA to obtain the vector pHPT-LoxP-rDNA-PFK (Table. S2). Subsequently, the plasmid was linearized by *Sma*I digestion and transformed into the Δ*pfk* strain. The positive transformants were screened on the YPGL plates with 100 μg/mL hygromycin B and then were verified by PCR analysis using the primers PFK-F/PFK-R to obtain the complement strain *epfk*.

### Yeast growth assays

P16, *epfk*, and Δ*pfk* were grown in YPD and YPGL media (initial OD_600_ = 0.01) and sampled at 3 h intervals to measure OD_600_ generate growth curves. For the spot assay, 50 μL of the YPGL overnight culture of these strains was diluted in water to an OD_600_ = 0.2, and then 2 μL of the diluted samples were spotted on solid YPD and YPGL media, followed by incubation for 2 days. For the cell survival rate assay, P16 and Δ*pfk* were cultured in YPGL medium for 12 h. Subsequently, 50 μL of the culture was transferred to 5 mL of YPD and sampled at 2-h intervals using the Cell Counting Kit-8 (Beyotime, China) for measurement.

### RNA sequencing

Total RNA of three biological replicates was extracted at 6 h, 18 h, 36 h, 60 h and 96 h during pullulan fermentation of *A. melanogenum* P16 (Fig. S1) using the Fungal RNA Kit (Omega, China). RNA concentration and purity were assessed using the Nanodrop 2000. RNA libraries were generated using the TruSeqTM RNA sample preparation kit (Illumina, USA), and sequenced on the Illumina Novaseq 6000 (2 × 150 pair-end). The clean data were blasted to the reference genome of the *A. melanogenum* P16 genome sequence (GenBank No. GCA_019915885.1), and the mapped data (reads) were obtained for subsequent analysis.

### Temporal gene expression profiles and KEGG enrichment analysis

The Short Time-series Expression Miner (STEM) program was used to analyze differentially expressed of genes and identify temporal expression profiles [47]. Five sampling time points (6 h, 18 h, 36 h, 60 h and 96 h) were designed according to the profile of pullulan titer (Fig. S1). Gene expression levels that met the 1.5-fold change criterion at any time point were used, and STEM profiles were clustered, with all parameters set to the default value. Temporal expression profiles that showed statistically significant variation from the time series were corrected using a false discovery rate (FDR) calculation performed on 1000 randomly selected permutations.

The DAVID database with Kyoto Encyclopedia of Genes and Genomes (KEGG) was used to pathway enrichment study of the functionally related gene groups in time series with *P* < 0.05 [48].

### Construction of a glucose-dependent selection system

The *PFK* gene and the promoter of *ADH* gene (P_*ADH*_), *GST* gene (P_*GST*_), and *PGK* gene (P_*PGK*_) were amplified from *A. melanogenum* P16 genome using primers PFK-F/PFK-R, ADH-F/ADH-R, GST-F/GST-R and PGK-F/PGK-R, respectively (Table S2). The *PFK* gene with the promoters of P_*PGK*_, P_*GST*_, and P_*ADH*_ was digested with *BamH*I/*Spe*I, and replaced the original antibiotic selection element (P_*PGK*_-NAT) in the plasmid pNAT-LoxP-rDNA (Fig. S1) to obtain the plasmids pP_*ADH*_-PFK-rDNA, pP_*GST*_-PFK-rDNA and pP_*PGK*_-PFK-rDNA (Table. S1).

### Fluorescent reporter gene expression and assessment of transformation efficiency

The green fluorescent protein gene (*GFP*) was cloned into the *Apa*I/*Xba*I site of the plasmids pP_*ADH*_-PFK-rDNA, pP_*GST*_-PFK-rDNA and pP_*PGK*_-PFK-rDNA, resulting in the plasmids of pP_*ADH*_-PFK-rDNA-GFP, pP_*GST*_- PFK-rDNA-GFP and pP_*PGK*_-PFK-rDNA-GFP (Table S1). Subsequently, these plasmids were linearized by *SmaI* digestion and transformed into the Δ*pfk* strain, followed by screening on YPD plates. As a control, the *GFP* gene was cloned into *Kpn*I/*Sca*I site of pNAT-LoxP-rDNA, resulting in the plasmid pNAT-LoxP-rDNA-GFP (Table S1). The obtained plasmid was linearized by *SmaI* digestion and transformed into P16., followed by screening on the YPD plates with 100 μg/mL nourseothricin.

Following transformation, all transformants underwent verification via genomic PCR using the primers GFP-F/GFP-R (Table. S2). The transformation efficiency was defined as the positive cfu numbers per 1.0 μg of the linear DNA fragments added to the mixture. The positive rate was defined as the percentage of transformants containing the *GFP* fragment among all transformants. Twenty positive transformants from each transformation were subjected to the *GFP* gene copy numbers analysis according to the method described below.

### Measurement of the stability of glucose-dependent selection systems

To assess the stability during the successive inoculations, the strains Δ*pfk*:P_*ADH*_-*PFK*:*GFP,* Δ*pfk*:P_*GST*_-*PFK*:*GFP* and Δ*pfk*:P_*PGK*_-*PFK*:*GFP* were inoculated in YPD medium, while P16:P_*TEF*_-*NAT*:*GFP* was inoculated in YPD medium with and without 100 μg/mL nourseothricin. The copy numbers of the *GFP* of these strains in each generation were measured according to the method described below.

To assess the stability during the pullulan fermentation, the strains Δ*pfk*:P_*ADH*_-*PFK*:*GFP,* Δ*pfk*:P_*GST*_-*PFK*:*GFP*, Δ*pfk*:P_*PGK*_-*PFK*:*GFP* and P16:P_*TEF*_-*NAT*:*GFP* were inoculated in pullulan production broth. Samples were collected at 24-h intervals to quantify the copy number of the *GFP*, the relative fluorescent intensity of yeast cells, and the relative transcript level of *PFK*, following the method described below.

### Microscopy and fluorescence intensity analysis

The obtained strains harboring *GFP* expression fragments were observed under blue light (488 nm) and white light using a 100 × oil immersion objective of an Olympus U-LH 100 HG fluorescence microscope.

The fluorescence intensity was measured using Varioskan^™^ LUX Multimode Microplate Reader (Thermo Fisher, USA) with an excitation wavelength of 480 nm and emission wavelength of 520 nm according to our previous method [49]. The yeast cells were resuspended in the sterile PBS and diluted until the OD_600nm_ value of the suspension reached 0.8. To standardize measurements and correct for background fluorescence, the fluorescence intensity of the Δ*pfk* strain was established as the control and set at 0%, while the fluorescence intensity of P16:P_*TEF*_-*NAT*:*GFP* was defined as 100%.

### RT-qPCR

Purification of total RNA, quantification of synthesized cDNAs by qPCR, and data analysis were carried out following the protocol as detailed in [49]. The β-actin gene was used as an internal reference. The primers used for RT-qPCR are listed in Table S2.

### Gene copy number analysis

The gene copy numbers of *GFP* in different yeast strains were analyzed by Quantitative Real-time PCR using genomic DNA as templates according to a previous study [49]. The glyceraldehyde-3-phosphate dehydrogenase gene (*GAPDH*) was used as a reference gene. A tenfold series (10^3^–10^8^ copies) of linearized plasmids containing *GFP* and the endogenous gene (*GAPDH*), were used as templates to establish standard curves associating Ct values with copy numbers. The Ct values of *GFP* and *GAPDH* in each strain were determined through RT-qPCR using genomic DNA as a template on a Rotor-Gene Q Real-time PCR Cycler (QIAGEN Hilden, Germany). The relative copy number of *GFP* was calculated based on the gene copy number ratio of *GFP* to *GAPDH*. The primers used for gene copy number analysis are listed in Table S2.

### Pullulan purification and quantification

The pullulan purification and quantitative determination were conducted following our previously reported method [24]. Briefly, the fermentation broth underwent centrifugation at 14,000 × *g* and 4 ℃ for 10 min to remove cells. Subsequently, two volumes of ethanol were added into the supernatant to precipitate pullulan. The resulting precipitate was dissolved in deionized water, and the ethanol precipitation step was repeated. The obtained pullulan was lyophilized and weighed.

### Phylogenetic tree construction

The ATP-dependent 6-phosphofructokinase (pfk) sequences of *A. melanogenum* and other fungi from the phyla of Basidiomycota and Ascomycota were utilized to construct a phylogenetic using MEGA11 with the maximum likelihood method. The fructose-1,6-bisphosphatase (fbp) from *S. cerevisiae* was used as an outgroup.

### Supplementary Information

Additional file 1: Fig S1. Time course of pullulan production and cell growth by *A. melanogenum* P16 during the 10-liter fermentation. Fig S2. Nourseothricin-dependent screening expression vector pNAT-Loxp-rDNA. Fig S3. The growth phenotype of Δ*pfk* on YPGL (glycerol and lactate) and YPF (fructose) media. Table S1. Yeast strains and plasmids used in this study. Table S2. Primers used in this study. Table S3. Gene expression model of 1122 genes from Profile 1.

## Data Availability

All data generated or analyzed during this study are included in this published article and its supplementary information files.
