# Supplementary material for: Customizable and stable multilocus chromosomal integration: a novel glucose-dependent selection system in Aureobasidium spp
Source: Biotechnol Biofuels Bioprod. 2024 Jun 17;17:81. doi: 10.1186/s13068-024-02531-3 (PMC11181563; doi:10.1186/s13068-024-02531-3)
Supplement: Supplementary file 1 — Additional file 1: Fig S1. Time course of pullulan production and cell growth by A. melanogenum P16 during the 10-liter fermentation. Fig S2. Nourseothricin-dependent screening expression vector pNAT-Loxp-rDNA. Fig S3. The growth phenotype of Δpfk on YPGL (glycerol and lactate) and YPF (fructose) media. Table S1. Yeast strains and plasmids used in this study. Table S2. Primers used in this study. Table S3. Gene expression model of 1122 genes from Profile 1. [file 13068_2024_2531_MOESM1_ESM.docx]

**Additional File Information**

**Customizable and stable multilocus** **chromosomal integration: A novel** **glucose-****dependent selection system in *Aureobasidium* spp.**

Shuo Zhang ^a^, Tao Ma ^a^, Fu-Hui Zheng ^a^, Muhammad Aslam ^c^, Yu-Jie Wang ^a^, Zhen-Ming Chi ^a, b^, Guang-Lei Liu ^a, b,^ *

**^a^** MOE Key Laboratory of Evolution and Marine Biodiversity, College of Marine Life Science, Ocean University of China, Yushan Road, No. 5, Qingdao, Shandong 266003, China.

**^b^** Laboratory for Marine Biology and Biotechnology, Qingdao Marine Science and Technology Center, No.1 Wenhai Road, 266237 Qingdao, China.

^c^ Faculty of Basic Sciences, Bolan University of Medical and Health Sciences, Quetta, 87600, Pakistan.

*Corresponding author: Guang-Lei Liu

E-mail: liugl@ouc.edu.cn

Tel and Fax: 0086-532-82032266


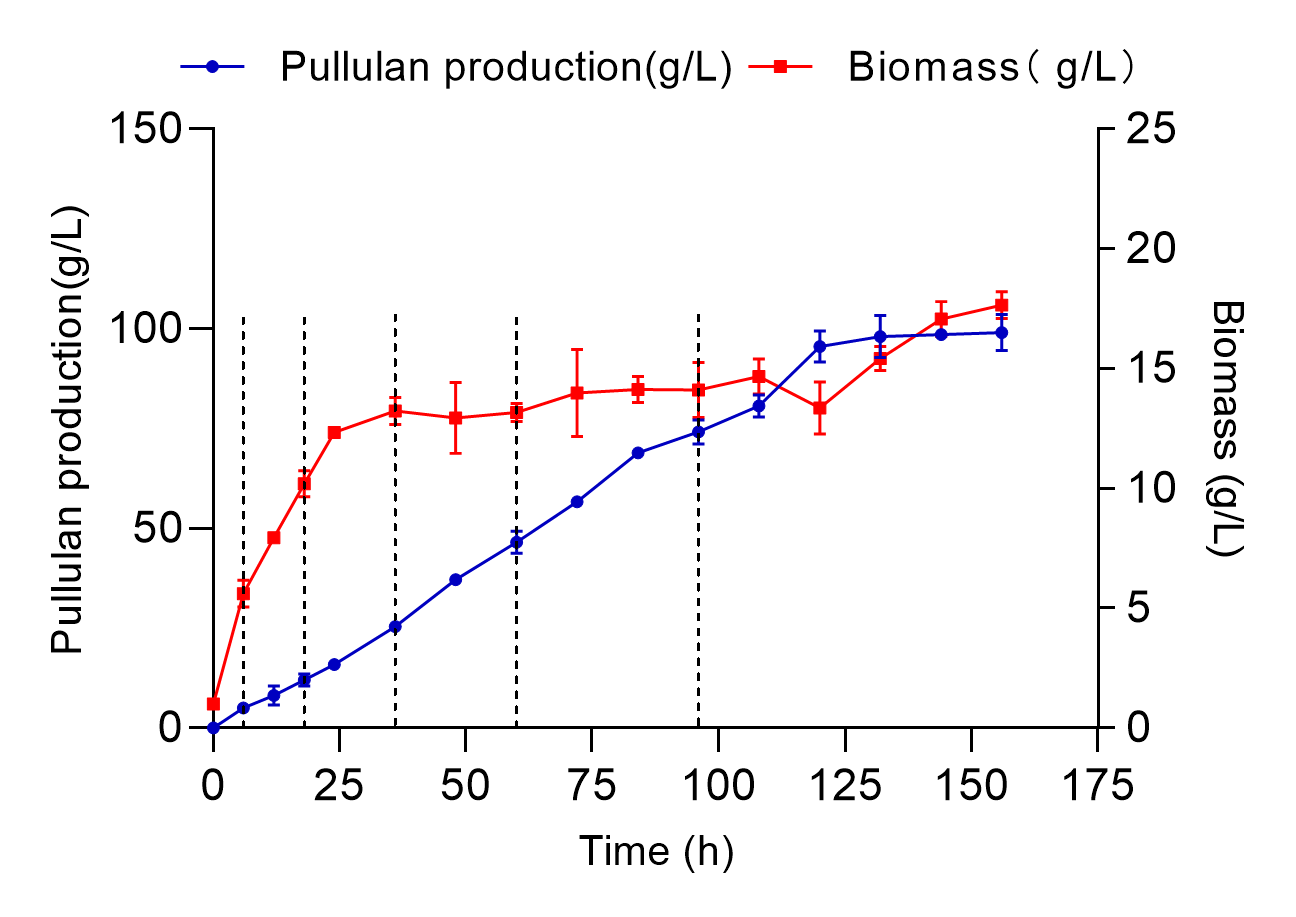


**Additional file 1: Fig. S1** Time course of pullulan production and cell growth by *A. melanogenum* P16 during the 10-liter fermentation. The time intervals for transcriptome analysis were indicated by the dotted lines (6h, 18 h, 36 h, 60 h, and 96 h).


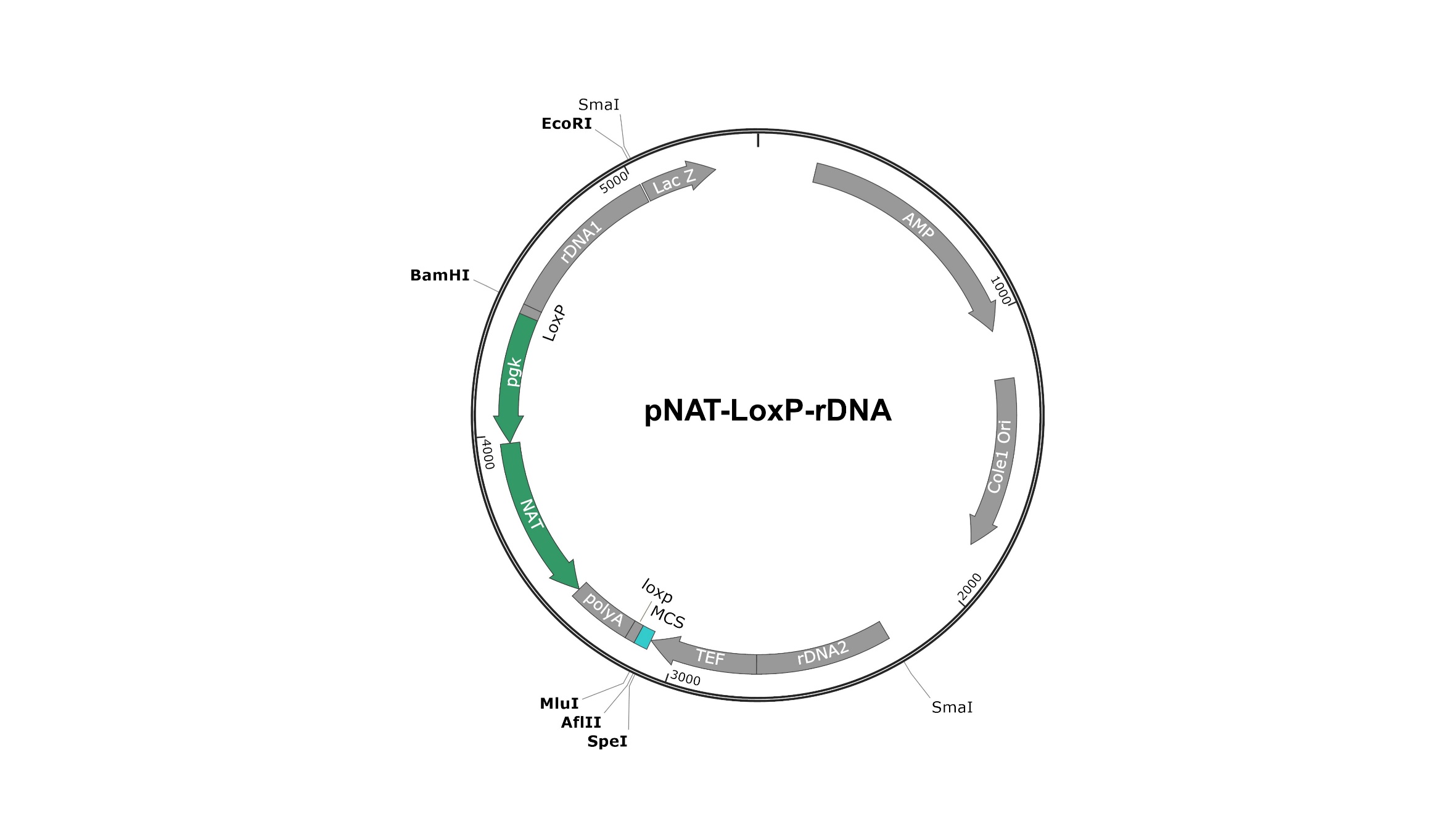
 **Additional file 1: Fig. S2** Nourseothricin-dependent screening expression vector pNAT-Loxp-rDNA.


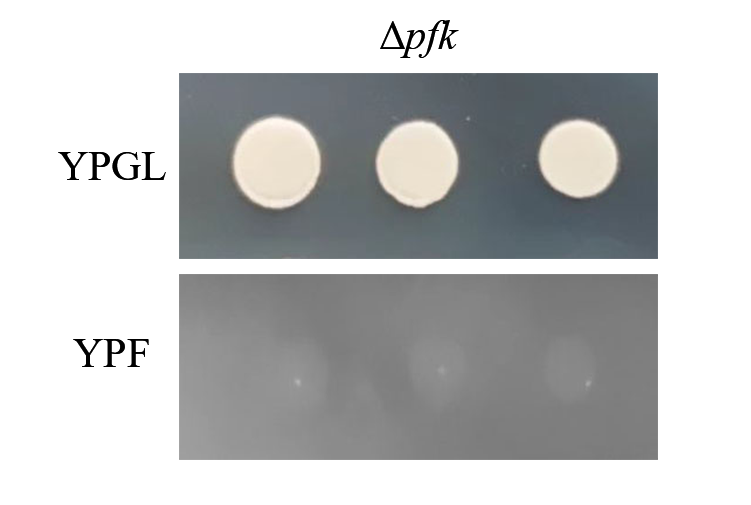


**Additional file 1: Fig. S3** The growth phenotype of Δ*pfk* on YPGL (glycerol and lactate) and YPF (fructose) media.

**Additional file 1: Table S1** Yeast strains and plasmids used in this study.

| Strains and plasmids | Characteristics | Source |
| --- | --- | --- |
| Strains |  |  |
| *Aureobasidium melanogenum* P16 | Wild-type | [23] |
| Δ*pfk-*N | *A. melanogenum* P16, *ΔPFK, NAT* | This study |
| Δ*pfk* | *A. melanogenum* P16, *ΔPFK* | This study |
| e*pfk* | Δ*pfk*, *HPT*, P*_PGK_-PFK* | This study |
| Δ*pfk*:P*_ADH_*-*PFK*:*GFP* | Δ*pfk*, P*_ADH_-PFK-*polyA*,* P*_PGK_-GFP* | This study |
| Δ*pfk*:P*_GST_*-*PFK*:*GFP* | Δ*pfk*, P*_GST_-PFK-*polyA*,* P*_PGK_-GFP* | This study |
| Δ*pfk*:P*_PGK_*-*PFK*:*GFP* | Δ*pfk*, P*_PGK_-PFK-*polyA*,* P*_PGK_-GFP* | This study |
| P16:P*_TEF_*- *NAT:GFP* | Δ*pfk*, P*_TEF_-HPT-*polyA*,* P*_PGK_-GFP* | This study |
| Plasmids |  |  |
| fl4a-NAT-LoxP | *NAT* cassette | [6] |
| pHPT-LoxP-rDNA | *HPT* cassette | [6] |
| pAMCRE-1 | *Shble* cassette | [6] |
| fl4a-NAT-LoxP-PFK | pfk5arm -*NAT* cassette-pfk3arm | This study |
| pHPT-LoxP-rDNA *-PFK* | rDNA-HPT cassette-P*_PGK_-PFK*-rDNA | This study |
| pNAT-LoxP- rDNA-GFP | rDNA-NAT cassette-P*_PGK_-GFP*-rDNA | This study |
| pP*_ADH_*-PFK-rDNA-GFP | rDNA-P*_ADH_* -*PFK-*polyA*-*P*_PGK_-GFP*-rDNA | This study |
| pP*_GST_*- PFK-rDNA*-GFP* | rDNA-P*_GST_*-*PFK-*polyA*-*P*_PGK_-GFP*-rDNA | This study |
| pP*_PGK_*- PFK-rDNA-GFP | rDNA-P*_PGK_*-*PFK-*polyA*-*P*_PGK_-GFP*-rDNA | This study |
| pNAT-LoxP-rDNA | rDNA-*NAT* cassette-rDNA | [6] |
| pP*_ADH_*-PFK-rDNA | rDNA-P*_ADH_* -*PFK-*polyA-rDNA | This study |
| pP*_GST_*-PFK-rDNA | rDNA-P*_GST_*-*PFK-*polyA-rDNA | This study |
| pP*_PGK_*-PFK-rDNA | rDNA-P*_PGK_*-*PFK-*polyA*-*rDNA | This study |

**Additional file 1: Table S2** Primers used in this study.

| Primer | Sequence | Use |
| --- | --- | --- |
| PFK-5F | 5’- ATGCATGCCATAGACGGGCTGGTGATAG -3’ | Amplification of PFK-5arm |
| PFK-5R | 5’- CACTGCAGTACGAATCTGAGGAGGGTTG-3’ |  |
| PFK-3F | 5’- CGGGATCCGACTTTGCTCTGCGTTGG -3’ | Amplification of PFK-3arm |
| PFK-3R | 5’- CGGAATTCTCTCGCTGGCCTTGAACT -3’ |  |
| PFK-F | 5’- GGATCCCAGCCTAGGATGGCCCAGGTCAACCTTGCTCCCC-3’ | Amplification of PFK |
| PFK-R | 5’- ACTAGTCTAGGGCCCCTAGCTAGCTGCTCT -3’ |  |
| GFP-F | 5’- TTGGGCCCATGGTGAGCAAGGGCGAGG -3’ | Amplification of a GFP |
| GFP-R | 5’- CTCTAGATTACTTGTACAGCTCGTCCATGC -3’ |  |
| ADH-F | 5’CGGATCCATAACTTCGTATAATGTATGCTATACGAACGGTAATCTCAATCAAGGCATGA -3’ | Amplification of P_ADH_ |
| ADH-R | 5’- GCCTAGGTTGTAAATTGGTTTCTACTTGG -3’ |  |
| GST-F | 5’CGGATCCATAACTTCGTATAATGTATGCTATACGAACGGTATTGGCTATCCGTGTACGA -3’ | Amplification of P_GST_ |
| GST-R | 5’- GCCTAGGAGAATCGAGTTTATAAAGGATG -3’ |  |
| PGK-F | 5’CGGATCCTACCGTTCGTATAATGTATGCTATACGAAGTTATATTACCTTCATGCATCCAT -3’ | Amplification of P_PGK_ |
| PGK-R | 5’- GCCTAGG TGTGACTGAATTGGATGTGT -3’ |  |
| QPGK-F | 5’- GAGGGCAGCAGCAAGGAT -3’ | Amplification of a fragment encoding PGK for qPCR |
| QPGK-R | 5’- TGAGCACGGTGAGCGGTA -3’ |  |
| QPK-F | 5’- GAACTTCTCCCACGGCTCA -3’ | Amplification of a fragment encoding PK for qPCR |
| QPK-R | 5’- TCAAGGGCAATGGCAACG -3’ |  |
| QGST-F | 5’- TCGCTTTGCTGGGAGATG -3’ | Amplification of a fragment encoding GST for qPCR |
| QGST-R | 5’- CCAAGATGAAATGGTCGTT -3’ |  |
| QADH-F | 5’- GTTCATACGTCGGCAACC -3’ | Amplification of a fragment encoding ADH for qPCR |
| QADH-R | 5’- GTCCCTTCTCCATCATCTCA -3’ |  |
| QNPA-F | 5’- CCGCAGACCCAAGAACAT -3’ | Amplification of a fragment encoding NPA for qPCR |
| QNPA-R | 5’- GCCTTACCCTTGTCGGAGT -3’ |  |
| QGAPDH-F | 5’- TCACCACCACCGAGAAGG -3’ | Amplification of a fragment encoding GAPDH for qPCR |
| QGAPDH-R | 5’- AAGAGGCGTTGGACAGGA -3’ |  |
| β-actin-F | 5’- CATCAACCCCAAGTCCAACCG -3’ | Amplification of a fragment encoding β-actin for qPCR |
| β-actin-R | 5’- CACCATCACCGGAGTCGAGGA -3’ |  |
| QGFP-F | 5’- ACGTAAACGGCCACAAGTTCAG -3’ | Amplification of a fragment encoding GFP for qPCR |
| QGFP-R | 5’- GTAGGTCAGGGTGGTCACGAGG -3’ |  |

**Additional file 1: Table S3** Gene expression model of 1122 genes from Profile 1.

| Gene ID | Gene description | Relative gene expression levels on a log_2_ scale* | | | | |
| --- | --- | --- | --- | --- | --- | --- |
|  |  | 6h | 18h | 36h | 60h | 96h |
| A09000 | KEQ59083.1; phenylacetate 2-hydroxylase [ *Aureobasidium* *melanogenum* CBS 110374] | 0 | 4.64 | 5.48 | 4.17 | 5.2 |
| A09001 | KEQ59084.1; hypothetical protein M437DRAFT_57939 [ *Aureobasidium* *melanogenum* CBS 110374] | 0 | 1.54 | 1.6 | 1.21 | 1.88 |
| A09024 | KEQ59023.1; hypothetical protein M437DRAFT_69384 [ *Aureobasidium* *melanogenum* CBS 110374] | 0 | 1.19 | 1.03 | 1.41 | 1.16 |
| A09075 | KEQ58177.1; hypothetical protein M437DRAFT_88787 [ *Aureobasidium* *melanogenum* CBS 110374] | 0 | 1.61 | 1.95 | 2.12 | 2.07 |
| A09093 | XP_013339554.1; hypothetical protein AUEXF2481DRAFT_44470 [ *Aureobasidium* *subglaciale* EXF-2481] | 0 | 2.36 | 3.9 | 3.89 | 4.03 |
| A00012 | NA | 0 | 2.14 | 2.48 | 2.39 | 2.57 |
| A00023 | KEQ59569.1; hypothetical protein M437DRAFT_56688 [ *Aureobasidium* *melanogenum* CBS 110374] | 0 | 1.11 | 1.62 | 1.45 | 1.21 |
| A00054 | KEQ59643.1; putative Stabilin-2 [ *Aureobasidium* *melanogenum* CBS 110374] | 0 | 2.11 | 2.53 | 2.71 | 2.47 |
| A00061 | XP_013427145.1; ATP-dependent RNA helicase dbp7 [ *Aureobasidium* *namibiae* CBS 147.97] | 0 | 1.79 | 2.91 | 3.03 | 3 |
| A00083 | KEQ59673.1; Amidophosphoribosyltransferase [ *Aureobasidium* *melanogenum* CBS 110374] | 0 | 1.34 | 1.63 | 1.74 | 1.16 |
| A00096 | KEQ59688.1; hypothetical protein M437DRAFT_69004 [ *Aureobasidium* *melanogenum* CBS 110374] | 0 | 2.73 | 2.98 | 2.78 | 2.98 |
| A09103 | KEQ59144.1; GMC oxidoreductase [ *Aureobasidium* *melanogenum* CBS 110374] | 0 | 2.16 | 2.3 | 2.86 | 3.31 |
| A09119 | XP_013428616.1; putative pathogenesis associated protein Pep2 [ *Aureobasidium* *namibiae* CBS 147.97] | 0 | 1.95 | 1.51 | 2.22 | 1.94 |
| A09133 | XP_013346582.1; hypothetical protein AUEXF2481DRAFT_2085 [ *Aureobasidium* *subglaciale* EXF-2481] | 0 | 1.01 | 0.95 | 0.69 | 0.71 |
| A09152 | KEQ65976.1; hypothetical protein M437DRAFT_63509 [ *Aureobasidium* *melanogenum* CBS 110374] | 0 | 3.38 | 4.12 | 2.53 | 4.17 |
| A09156 | NA | 0 | 2.27 | 1.84 | 1.66 | 1.26 |
| A09181 | NA | 0 | 3.88 | 4.22 | 3.54 | 5.2 |
| A09184 | KEQ85643.1; hypothetical protein M438DRAFT_354688 [ *Aureobasidium* *pullulans* EXF-150] | 0 | 1.35 | 1.64 | 1.74 | 1.64 |
| A09197 | KEQ64063.1; asparagine synthase [ *Aureobasidium* *melanogenum* CBS 110374] | 0 | 1.06 | 1.8 | 2.07 | 1.9 |
| A09203 | KEQ85634.1; hypothetical protein M438DRAFT_344921 [ *Aureobasidium* *pullulans* EXF-150] | 0 | 1.98 | 1.48 | 1.1 | 0.92 |
| A09226 | XP_013427259.1; putative Stabilin-2 [ *Aureobasidium* *namibiae* CBS 147.97] | 0 | 1.51 | 2.12 | 2.06 | 1.65 |
| A09251 | XP_013427142.1; long-chain fatty acid transporter-like protein [ *Aureobasidium* *namibiae* CBS 147.97] | 0 | 0.93 | 1.2 | 1.2 | 1.23 |
| A09254 | KEQ59668.1; acetyl-CoA synthetase-like protein [ *Aureobasidium* *melanogenum* CBS 110374] | 0 | 1.08 | 1.41 | 1.52 | 1.37 |
| A09300 | XP_013430211.1; hypothetical protein M436DRAFT_40621 [ *Aureobasidium* *namibiae* CBS 147.97] | 0 | 2.29 | 1.6 | 2.52 | 2.09 |
| A09313 | XP_013344844.1; hypothetical protein AUEXF2481DRAFT_4104 [ *Aureobasidium* *subglaciale* EXF-2481] | 0 | 1.5 | 2.1 | 2.39 | 2.06 |
| A09323 | XP_013339925.1; hypothetical protein AUEXF2481DRAFT_33060 [ *Aureobasidium* *subglaciale* EXF-2481] | 0 | 1.5 | 1.73 | 2.14 | 2.07 |
| A09347 | KEQ85380.1; choline transport protein [ *Aureobasidium* *pullulans* EXF-150] | 0 | 2.14 | 1.88 | 2 | 2.31 |
| A09374 | XP_013339920.1; hypothetical protein AUEXF2481DRAFT_33010 [ *Aureobasidium* *subglaciale* EXF-2481] | 0 | 2.3 | 2.48 | 2.83 | 1.91 |
| A09388 | XP_013427231.1; hypothetical protein M436DRAFT_82113 [ *Aureobasidium* *namibiae* CBS 147.97] | 0 | 2.71 | 1.91 | 1.98 | 3.24 |
| A09392 | XP_013344863.1; hypothetical protein AUEXF2481DRAFT_4163 [ *Aureobasidium* *subglaciale* EXF-2481] | 0 | 1.56 | 1.88 | 1.99 | 1.32 |
| A09412 | XP_013430348.1; ARM repeat-containing protein [ *Aureobasidium* *namibiae* CBS 147.97] | 0 | 1.68 | 2.31 | 2.1 | 1.7 |
| A09420 | KEQ59591.1; GMP synthase subunit B [ *Aureobasidium* *melanogenum* CBS 110374] | 0 | 1.11 | 0.93 | 1.33 | 1.29 |
| A09439 | XP_013422934.1; putative beta-galactosidase B [ *Aureobasidium* *namibiae* CBS 147.97] | 0 | 3.01 | 3.49 | 3.22 | 2.93 |
| A09480 | XP_013422882.1; NAD(P)-binding protein [ *Aureobasidium* *namibiae* CBS 147.97] | 0 | 1.76 | 2 | 2.07 | 1.56 |
| A09485 | KEQ61984.1; hypothetical protein M437DRAFT_66924 [ *Aureobasidium* *melanogenum* CBS 110374] | 0 | 1.36 | 1.97 | 1.81 | 1.63 |
| A09499 | KEQ63691.1; hypothetical protein M437DRAFT_74286 [ *Aureobasidium* *melanogenum* CBS 110374] | 0 | 1.49 | 1.7 | 1.67 | 1.03 |
| A09539 | KEQ65836.1; amidase signature enzyme [ *Aureobasidium* *melanogenum* CBS 110374] | 0 | 2.7 | 3.82 | 3.35 | 3.58 |
| A09542 | KEQ65828.1; MFS general substrate transporter [ *Aureobasidium* *melanogenum* CBS 110374] | 0 | 2.5 | 3.81 | 3.48 | 2.92 |
| A09592 | KEQ78579.1; delta-1-pyrroline-5-carboxylate dehydrogenase 1 [ *Aureobasidium* *pullulans* EXF-150] | 0 | 2.38 | 2.54 | 2.32 | 2.71 |
| A09633 | XP_013422523.1; DUF1479-domain-containing protein [ *Aureobasidium* *namibiae* CBS 147.97] | 0 | 0.89 | 0.86 | 1.12 | 0.73 |
| A09636 | KEQ64419.1; hypothetical protein M437DRAFT_83354 [ *Aureobasidium* *melanogenum* CBS 110374] | 0 | 3.24 | 4.19 | 4.66 | 4.91 |
| A09637 | KEQ82461.1; hypothetical protein M438DRAFT_366966 [ *Aureobasidium* *pullulans* EXF-150] | 0 | 3.01 | 3.75 | 3.89 | 3.94 |
| A09646 | KEQ64327.1; hypothetical protein M437DRAFT_83264 [ *Aureobasidium* *melanogenum* CBS 110374] | 0 | 3.85 | 4.64 | 4.93 | 4.17 |
| A09650 | KEQ82473.1; hypothetical protein M438DRAFT_347244 [ *Aureobasidium* *pullulans* EXF-150] | 0 | 1.51 | 1.74 | 2.31 | 1.77 |
| A09659 | XP_013346075.1; hypothetical protein AUEXF2481DRAFT_602122 [ *Aureobasidium* *subglaciale* EXF-2481] | 0 | 1.64 | 1.36 | 1.93 | 1.31 |
| A09671 | KEQ64353.1; Clavaminate synthase-like protein [ *Aureobasidium* *melanogenum* CBS 110374] | 0 | 2.47 | 2.82 | 3.01 | 2.72 |
| A09673 | KEQ64355.1; hypothetical protein M437DRAFT_45069 [ *Aureobasidium* *melanogenum* CBS 110374] | 0 | 1.1 | 1.63 | 1.85 | 1.49 |
| A09677 | KEQ64359.1; endo-1,4-beta-glucanase precursor [ *Aureobasidium* *melanogenum* CBS 110374] | 0 | 0.8 | 1.53 | 1.49 | 1.47 |
| A09678 | KEQ64360.1; hypothetical protein M437DRAFT_83296 [ *Aureobasidium* *melanogenum* CBS 110374] | 0 | 3.05 | 1.44 | 1.78 | 1.77 |
| A09681 | KEQ64363.1; hypothetical protein M437DRAFT_83298 [ *Aureobasidium* *melanogenum* CBS 110374] | 0 | 2.55 | 3.22 | 3.27 | 3.77 |
| A09706 | KEQ64272.1; alcohol oxidase [ *Aureobasidium* *melanogenum* CBS 110374] | 0 | 1.16 | 0.7 | 1.14 | 0.76 |
| A09727 | KEQ64297.1; hypothetical protein M437DRAFT_44886 [ *Aureobasidium* *melanogenum* CBS 110374] | 0 | 1.97 | 1.97 | 2.82 | 2.89 |
| A09739 | KEQ64384.1; endo-polygalacturonase [ *Aureobasidium* *melanogenum* CBS 110374] | 0 | 0.96 | 1.2 | 0.88 | 1.22 |
| A09747 | XP_013424734.1; gluconate 5-dehydrogenase [ *Aureobasidium* *namibiae* CBS 147.97] | 0 | 1.31 | 0.8 | 1.42 | 1.04 |
| A09754 | KEQ64265.1; hypothetical protein M437DRAFT_44932 [ *Aureobasidium* *melanogenum* CBS 110374] | 0 | 1.14 | 1.16 | 0.96 | 0.85 |
| A09781 | KEQ64415.1; hypothetical protein M437DRAFT_83350 [ *Aureobasidium* *melanogenum* CBS 110374] | 0 | 1.41 | 1.56 | 1.89 | 1.7 |
| A09788 | KEQ64409.1; hypothetical protein M437DRAFT_83344 [ *Aureobasidium* *melanogenum* CBS 110374] | 0 | 2.08 | 1.71 | 1.43 | 1.26 |
| A09789 | XP_013345722.1; hypothetical protein AUEXF2481DRAFT_3326 [ *Aureobasidium* *subglaciale* EXF-2481] | 0 | 1.72 | 1.15 | 1.51 | 2.24 |
| A09815 | KEQ64309.1; Pre-rRNA-processing protein PNO1 [ *Aureobasidium* *melanogenum* CBS 110374] | 0 | 1.85 | 2.78 | 2.99 | 2.54 |
| A09819 | KEQ64236.1; phosphoglycerate mutase-like protein [ *Aureobasidium* *melanogenum* CBS 110374] | 0 | 1.22 | 1.19 | 0.78 | 1.63 |
| A09868 | KEQ64133.1; hypothetical protein M437DRAFT_73977 [ *Aureobasidium* *melanogenum* CBS 110374] | 0 | 1.49 | 0.97 | 1.26 | 1.98 |
| A09895 | KEQ64161.1; HAD-superfamily hydrolase [ *Aureobasidium* *melanogenum* CBS 110374] | 0 | 1.92 | 2.27 | 2.18 | 2.34 |
| A09923 | KEQ64262.1; TFIIB-domain-containing protein [ *Aureobasidium* *melanogenum* CBS 110374] | 0 | 0.57 | 0.76 | 1.02 | 0.93 |
| A09934 | KEQ64250.1; hypothetical protein M437DRAFT_83193 [ *Aureobasidium* *melanogenum* CBS 110374] | 0 | 2.13 | 2 | 2.13 | 1.63 |
| A09936 | XP_013424543.1; hypothetical protein M436DRAFT_53515 [ *Aureobasidium* *namibiae* CBS 147.97] | 0 | 1.32 | 0.89 | 1.4 | 1.32 |
| A09944 | KEQ64096.1; hypothetical protein M437DRAFT_44660 [ *Aureobasidium* *melanogenum* CBS 110374] | 0 | 1.43 | 0.66 | 0.73 | 0.94 |
| A09961 | KEQ64076.1; hypothetical protein M437DRAFT_83018 [ *Aureobasidium* *melanogenum* CBS 110374] | 0 | 1.44 | 0.91 | 1.38 | 1.81 |
| A09970 | KEQ64071.1; hypothetical protein M437DRAFT_45271 [ *Aureobasidium* *melanogenum* CBS 110374] | 0 | 2.29 | 2.45 | 2.68 | 1.81 |
| A09979 | KEQ64053.1; NAD(P)-binding protein [ *Aureobasidium* *melanogenum* CBS 110374] | 0 | 3.39 | 3.87 | 5.52 | 5.31 |
| A09996 | XP_013340041.1; hypothetical protein AUEXF2481DRAFT_43913 [ *Aureobasidium* *subglaciale* EXF-2481] | 0 | 3.09 | 3.58 | 3.7 | 3.75 |
| A09997 | XP_013424658.1; hypothetical protein M436DRAFT_66398 [ *Aureobasidium* *namibiae* CBS 147.97] | 0 | 1.56 | 0.63 | 0.93 | 0.91 |
| A10059 | XP_013343571.1; glycoside hydrolase family 31 protein [ *Aureobasidium* *subglaciale* EXF-2481] | 0 | 2.17 | 3.98 | 3.94 | 3.8 |
| A10080 | KEQ58200.1; hypothetical protein M437DRAFT_79194 [ *Aureobasidium* *melanogenum* CBS 110374] | 0 | 1.51 | 1.47 | 1.47 | 0.83 |
| A10112 | KEQ58150.1; NAD(P)-binding protein [ *Aureobasidium* *melanogenum* CBS 110374] | 0 | 1.61 | 1.54 | 2.24 | 2.28 |
| A10123 | KEQ58162.1; hypothetical protein M437DRAFT_60032 [ *Aureobasidium* *melanogenum* CBS 110374] | 0 | 2.9 | 3.05 | 2.88 | 2.28 |
| A10134 | KEQ58231.1; hypothetical protein M437DRAFT_59858 [ *Aureobasidium* *melanogenum* CBS 110374] | 0 | 2.39 | 2.69 | 2.99 | 2.79 |
| A10162 | NA | 0 | 1.87 | 2.85 | 3.56 | 3.04 |
| A10183 | KEQ59023.1; hypothetical protein M437DRAFT_69384 [ *Aureobasidium* *melanogenum* CBS 110374] | 0 | 1.48 | 1.23 | 1.94 | 1.79 |
| A10201 | KEQ86470.1; hypothetical protein M438DRAFT_395694 [ *Aureobasidium* *pullulans* EXF-150] | 0 | 1.26 | 1.63 | 1.13 | 1.51 |
| A10210 | XP_013429119.1; hypothetical protein M436DRAFT_43292 [ *Aureobasidium* *namibiae* CBS 147.97] | 0 | 1.04 | 1.65 | 1.45 | 1.26 |
| A10225 | XP_013428968.1; hypothetical protein M436DRAFT_42484 [ *Aureobasidium* *namibiae* CBS 147.97] | 0 | 2.84 | 1.23 | 1.93 | 1.84 |
| A10238 | OBW66764.1; Signal recognition particle protein [ *Aureobasidium* *pullulans*] | 0 | 2.31 | 3.18 | 3.11 | 3.39 |
| A10267 | KEQ58990.1; hypothetical protein M437DRAFT_87944 [ *Aureobasidium* *melanogenum* CBS 110374] | 0 | 1.62 | 1.04 | 1.17 | 1.69 |
| A10271 | KEQ58994.1; hypothetical protein M437DRAFT_57724 [ *Aureobasidium* *melanogenum* CBS 110374] | 0 | 1.9 | 2.16 | 2.18 | 2.56 |
| A10317 | EXK77188.1; hypothetical protein FOQG_18096 [Fusarium oxysporum f. sp. raphani 54005] | 0 | 1.91 | 1.85 | 2.19 | 2.52 |
| A10324 | KEQ79812.1; putative P450 monooxygenase [ *Aureobasidium* *pullulans* EXF-150] | 0 | 1.01 | 1.08 | 1.13 | 0.8 |
| A10331 | NA | 0 | 3.07 | 3.47 | 3.58 | 3.4 |
| A10334 | XP_013343447.1; hypothetical protein AUEXF2481DRAFT_29752 [ *Aureobasidium* *subglaciale* EXF-2481] | 0 | 3.24 | 4.57 | 4.05 | 3.25 |
| A10360 | KEQ61075.1; hypothetical protein M437DRAFT_76652 [ *Aureobasidium* *melanogenum* CBS 110374] | 0 | 1.08 | 0.9 | 1.06 | 1.01 |
| A10361 | KEQ61076.1; hypothetical protein M437DRAFT_67463 [ *Aureobasidium* *melanogenum* CBS 110374] | 0 | 1.87 | 1.94 | 2.05 | 2.04 |
| A10384 | XP_013343635.1; hypothetical protein AUEXF2481DRAFT_226298 [ *Aureobasidium* *subglaciale* EXF-2481] | 0 | 3.06 | 1.87 | 1.43 | 2.21 |
| A10416 | XP_013343481.1; hypothetical protein AUEXF2481DRAFT_40122 [ *Aureobasidium* *subglaciale* EXF-2481] | 0 | 1.98 | 2.23 | 2.3 | 1.97 |
| A10426 | OBW66188.1; DUF1348-domain-containing protein [ *Aureobasidium* *pullulans*] | 0 | 3.11 | 2.38 | 2.99 | 4.06 |
| A10431 | XP_013429227.1; sugar transporter [ *Aureobasidium* *namibiae* CBS 147.97] | 0 | 1.49 | 2.15 | 2.48 | 2.45 |
| A10441 | XP_013343649.1; hypothetical protein AUEXF2481DRAFT_228837 [ *Aureobasidium* *subglaciale* EXF-2481] | 0 | 1.48 | 1.59 | 2.02 | 1.92 |
| A10454 | NA | 0 | 1.14 | 1.54 | 1.53 | 1.61 |
| A10456 | OBW67471.1; Uncharacterized protein AUREO_024550 [ *Aureobasidium* *pullulans*] | 0 | 2.67 | 2.39 | 3.2 | 4.09 |
| A10458 | NA | 0 | 1.92 | 2.46 | 2.04 | 3 |
| A10510 | XP_013423745.1; hypothetical protein M436DRAFT_85294 [ *Aureobasidium* *namibiae* CBS 147.97] | 0 | 2.73 | 2.78 | 2.68 | 3.23 |
| A10536 | NA | 0 | 3.5 | 4.23 | 4.72 | 4.97 |
| A10557 | OBW67418.1; Molybdopterin binding oxidoreductase [ *Aureobasidium* *pullulans*] | 0 | 2.7 | 4.03 | 3.32 | 2.81 |
| A10580 | KEQ58601.1; hypothetical protein M437DRAFT_78838 [ *Aureobasidium* *melanogenum* CBS 110374] | 0 | 2.96 | 4.76 | 4.34 | 4.33 |
| A10595 | KEQ58609.1; hypothetical protein M437DRAFT_69766 [ *Aureobasidium* *melanogenum* CBS 110374] | 0 | 2.81 | 3.33 | 3.93 | 3.88 |
| A10649 | XP_018041582.1; pectin lyase-like protein, partial [Paraphaeosphaeria sporulosa] | 0 | 1.22 | 1.22 | 1.04 | 1.09 |
| A10688 | PNS16356.1; Ribonuclease H1 [Sphaceloma murrayae] | 0 | 5.45 | 4.44 | 4.42 | 5.09 |
| A10698 | XP_013432414.1; hypothetical protein M436DRAFT_77741 [ *Aureobasidium* *namibiae* CBS 147.97] | 0 | 0.81 | 0.84 | 1.01 | 0.77 |
| A10715 | KEQ67897.1; MFS general substrate transporter [ *Aureobasidium* *melanogenum* CBS 110374] | 0 | 2.44 | 3.4 | 3.33 | 2.83 |
| A10717 | XP_013344239.1; hypothetical protein AUEXF2481DRAFT_39721 [ *Aureobasidium* *subglaciale* EXF-2481] | 0 | 3.22 | 2.76 | 4.08 | 3.59 |
| A10750 | KEQ61758.1; Na+/H+ antiporter [ *Aureobasidium* *melanogenum* CBS 110374] | 0 | 1.17 | 1.3 | 1.09 | 0.83 |
| A10751 | KEQ61743.1; hypothetical protein M437DRAFT_51250 [ *Aureobasidium* *melanogenum* CBS 110374] | 0 | 1.33 | 1.94 | 1.96 | 2.01 |
| A10761 | XP_013344175.1; hypothetical protein AUEXF2481DRAFT_29261 [ *Aureobasidium* *subglaciale* EXF-2481] | 0 | 3.9 | 3.43 | 4.05 | 4.97 |
| A10781 | XP_013344100.1; hypothetical protein AUEXF2481DRAFT_4791 [ *Aureobasidium* *subglaciale* EXF-2481] | 0 | 0.98 | 1.01 | 1.08 | 0.99 |
| A10811 | KEQ61729.1; hypothetical protein M437DRAFT_67182 [ *Aureobasidium* *melanogenum* CBS 110374] | 0 | 3.74 | 4.01 | 4.52 | 4.51 |
| A10850 | KEQ60278.1; alpha/beta hydrolase [ *Aureobasidium* *melanogenum* CBS 110374] | 0 | 1.01 | 0.8 | 1.11 | 0.6 |
| A10881 | KEQ60266.1; glycoside hydrolase [ *Aureobasidium* *melanogenum* CBS 110374] | 0 | 1.9 | 1.74 | 2.25 | 1.74 |
| A10899 | OBW64545.1; Uncharacterized protein AUREO_053920 [ *Aureobasidium* *pullulans*] | 0 | 6.28 | 4.88 | 5.7 | 6.27 |
| A10900 | XP_013431824.1; uracil-5-carboxylate decarboxylase [ *Aureobasidium* *namibiae* CBS 147.97] | 0 | 4.08 | 2.17 | 3.31 | 3.31 |
| A10903 | KEQ60247.1; acetamidase [ *Aureobasidium* *melanogenum* CBS 110374] | 0 | 4.27 | 2.18 | 3.48 | 3.39 |
| A10905 | OBW65763.1; hypothetical protein AUREO_041650 [ *Aureobasidium* *pullulans*] | 0 | 1.84 | 1.69 | 1.73 | 2.19 |
| A10929 | XP_013342030.1; hypothetical protein AUEXF2481DRAFT_31234 [ *Aureobasidium* *subglaciale* EXF-2481] | 0 | 1.22 | 1.38 | 1.48 | 1.41 |
| A10934 | KEQ66980.1; hypothetical protein M437DRAFT_36985 [ *Aureobasidium* *melanogenum* CBS 110374] | 0 | 3.39 | 3.13 | 3.46 | 3.55 |
| A10944 | KEQ78319.1; hypothetical protein M438DRAFT_350501 [ *Aureobasidium* *pullulans* EXF-150] | 0 | 1.22 | 0.5 | 0.7 | 0.78 |
| A10956 | OBW64400.1; Uncharacterized protein AUREO_055330 [ *Aureobasidium* *pullulans*] | 0 | 1.11 | 1.37 | 1.52 | 1.33 |
| A11012 | OBW69162.1; Trafficking protein particle complex subunit 5 [ *Aureobasidium* *pullulans*] | 0 | 1.03 | 0.53 | 0.45 | 0.62 |
| A11089 | KEQ83376.1; hypothetical protein M438DRAFT_346324 [ *Aureobasidium* *pullulans* EXF-150] | 0 | 1.04 | 1.14 | 0.96 | 0.9 |
| A11099 | OBW69466.1; Sybindin-like protein [ *Aureobasidium* *pullulans*] | 0 | 0.73 | 0.79 | 0.91 | 1.18 |
| A11107 | OBW69177.1; Uncharacterized protein AUREO_007770 [ *Aureobasidium* *pullulans*] | 0 | 0.93 | 1.51 | 1.51 | 1.35 |
| A11110 | KEQ67066.1; hypothetical protein M437DRAFT_36266 [ *Aureobasidium* *melanogenum* CBS 110374] | 0 | 1.52 | 2.09 | 1.94 | 2 |
| A11150 | XP_013423487.1; Pentulose kinase [ *Aureobasidium* *namibiae* CBS 147.97] | 0 | 1.56 | 1.08 | 1.72 | 1.67 |
| A11155 | KEQ82770.1; hypothetical protein M438DRAFT_47233 [ *Aureobasidium* *pullulans* EXF-150] | 0 | 4.54 | 4.29 | 5.2 | 4.92 |
| A11159 | KEQ67405.1; hypothetical protein M437DRAFT_37325 [ *Aureobasidium* *melanogenum* CBS 110374] | 0 | 3.64 | 3.95 | 5.05 | 5.02 |
| A11160 | KEQ67406.1; hypothetical protein M437DRAFT_71222 [ *Aureobasidium* *melanogenum* CBS 110374] | 0 | 2.6 | 2.44 | 3.56 | 3.13 |
| A11164 | KEQ81056.1; L-amino acid oxidase-like protein LaoA [ *Aureobasidium* *pullulans* EXF-150] | 0 | 1.53 | 1.13 | 1.75 | 1.22 |
| A11179 | KEQ82746.1; uricase [ *Aureobasidium* *pullulans* EXF-150] | 0 | 1.36 | 1.7 | 1.9 | 1.8 |
| A11185 | KEQ67512.1; hypothetical protein M437DRAFT_61911 [ *Aureobasidium* *melanogenum* CBS 110374] | 0 | 3.18 | 3.99 | 4.86 | 5.2 |
| A11229 | OBW69813.1; Uncharacterized protein AUREO_001370 [ *Aureobasidium* *pullulans*] | 0 | 1.37 | 1.5 | 1.19 | 1.26 |
| A11242 | KEQ81059.1; hypothetical protein M438DRAFT_338433 [ *Aureobasidium* *pullulans* EXF-150] | 0 | 2.03 | 2.04 | 1.76 | 1.21 |
| A11287 | KEQ80133.1; hypothetical protein M438DRAFT_326939 [ *Aureobasidium* *pullulans* EXF-150] | 0 | 1.88 | 1.13 | 1.3 | 0.98 |
| A11319 | KEQ62592.1; hypothetical protein M437DRAFT_49709 [ *Aureobasidium* *melanogenum* CBS 110374] | 0 | 1.33 | 2.35 | 2.23 | 2.48 |
| A11351 | KEQ82823.1; hypothetical protein M438DRAFT_347058 [ *Aureobasidium* *pullulans* EXF-150] | 0 | 1.2 | 1.94 | 1.54 | 1.71 |
| A11355 | KEQ82828.1; beta subunit of 3-methylcrotonyl-CoA carboxylase [ *Aureobasidium* *pullulans* EXF-150] | 0 | 2.79 | 2.7 | 3.57 | 3.59 |
| A11356 | OBW65547.1; Uncharacterized protein AUREO_043910 [ *Aureobasidium* *pullulans*] | 0 | 2.81 | 2.38 | 3.9 | 3.87 |
| A11369 | KEQ82840.1; FAD/NAD(P)-binding domain-containing protein [ *Aureobasidium* *pullulans* EXF-150] | 0 | 1.11 | 0.96 | 0.65 | 0.76 |
| A11380 | XP_013432351.1; hypothetical protein M436DRAFT_77382 [ *Aureobasidium* *namibiae* CBS 147.97] | 0 | 2.59 | 3.41 | 3.86 | 3.75 |
| A11381 | KEQ67382.1; Aldo/keto reductase [ *Aureobasidium* *melanogenum* CBS 110374] | 0 | 1.53 | 0.77 | 0.74 | 0.85 |
| A11384 | KEQ67378.1; hypothetical protein M437DRAFT_37854 [ *Aureobasidium* *melanogenum* CBS 110374] | 0 | 2.52 | 1.73 | 3.03 | 3.07 |
| A11399 | XP_013342444.1; hypothetical protein AUEXF2481DRAFT_320101 [ *Aureobasidium* *subglaciale* EXF-2481] | 0 | 0.91 | 1.27 | 1.21 | 1 |
| A11402 | XP_013432207.1; TrkH-domain-containing protein [ *Aureobasidium* *namibiae* CBS 147.97] | 0 | 1.59 | 1.48 | 1.07 | 0.91 |
| A11443 | KEQ79449.1; NAD(P)-binding protein [ *Aureobasidium* *pullulans* EXF-150] | 0 | 1 | 1.5 | 1.32 | 1.11 |
| A11460 | KEQ67304.1; NAD(P)-binding protein [ *Aureobasidium* *melanogenum* CBS 110374] | 0 | 1.2 | 0.83 | 1 | 0.84 |
| A11463 | XP_013342295.1; hypothetical protein AUEXF2481DRAFT_30978 [ *Aureobasidium* *subglaciale* EXF-2481] | 0 | 2.99 | 2.9 | 2.17 | 2.19 |
| A11509 | XP_013431661.1; hypothetical protein M436DRAFT_35355 [ *Aureobasidium* *namibiae* CBS 147.97] | 0 | 1.51 | 1.35 | 1.22 | 1.19 |
| A11511 | OBW69924.1; Uncharacterized protein AUREO_000330 [ *Aureobasidium* *pullulans*] | 0 | 1.34 | 2 | 2.23 | 1.81 |
| A11525 | KEQ67236.1; hypothetical protein M437DRAFT_79890 [ *Aureobasidium* *melanogenum* CBS 110374] | 0 | 2.23 | 3.07 | 3.09 | 2.56 |
| A11537 | XP_013342332.1; hypothetical protein AUEXF2481DRAFT_41546 [ *Aureobasidium* *subglaciale* EXF-2481] | 0 | 1.62 | 2.46 | 2.56 | 2.36 |
| A11554 | KEQ67529.1; hypothetical protein M437DRAFT_25889, partial [ *Aureobasidium* *melanogenum* CBS 110374] | 0 | 2.66 | 1.65 | 1.85 | 2.87 |
| A11556 | KEQ67531.1; kinase-like protein [ *Aureobasidium* *melanogenum* CBS 110374] | 0 | 1.63 | 2.13 | 2.37 | 1.99 |
| A11570 | XP_013342271.1; hypothetical protein AUEXF2481DRAFT_30860 [ *Aureobasidium* *subglaciale* EXF-2481] | 0 | 1.59 | 1.42 | 1.51 | 1.59 |
| A11575 | KEQ81026.1; nitrate transporter [ *Aureobasidium* *pullulans* EXF-150] | 0 | 3.85 | 4.75 | 3.81 | 3.62 |
| A11599 | XP_013342316.1; hypothetical protein AUEXF2481DRAFT_41475 [ *Aureobasidium* *subglaciale* EXF-2481] | 0 | 2.36 | 2.44 | 3.42 | 3.63 |
| A11610 | KEQ67874.1; putative aldo-keto reductase [ *Aureobasidium* *melanogenum* CBS 110374] | 0 | 2.72 | 2.31 | 1.78 | 1.51 |
| A11626 | KEQ78610.1; small oligopeptide transporter-like protein [ *Aureobasidium* *pullulans* EXF-150] | 0 | 2.22 | 1.45 | 1.16 | 1.8 |
| A11632 | XP_013431857.1; putative alpha-galactosidase [ *Aureobasidium* *namibiae* CBS 147.97] | 0 | 1.96 | 2.16 | 1.63 | 2.89 |
| A11654 | XP_013344288.1; glycoside hydrolase family 10 protein [ *Aureobasidium* *subglaciale* EXF-2481] | 0 | 2.11 | 2.13 | 2.44 | 2.2 |
| A11665 | XP_013431923.1; alpha-amylase [ *Aureobasidium* *namibiae* CBS 147.97] | 0 | 0.69 | 1.28 | 1 | 1.35 |
| A11666 | ADN65120.1; extracellular glucoamylase protein [ *Aureobasidium* *pullulans*] | 0 | 0.86 | 0.81 | 1.15 | 1.13 |
| A11694 | XP_013432404.1; hypothetical protein M436DRAFT_77688 [ *Aureobasidium* *namibiae* CBS 147.97] | 0 | 1.75 | 2.09 | 2.42 | 2.73 |
| A11700 | XP_013344193.1; hypothetical protein AUEXF2481DRAFT_39469 [ *Aureobasidium* *subglaciale* EXF-2481] | 0 | 2.59 | 2.52 | 3.48 | 3.7 |
| A11731 | XP_013344335.1; hypothetical protein AUEXF2481DRAFT_680517 [ *Aureobasidium* *subglaciale* EXF-2481] | 0 | 2.08 | 1.76 | 1.29 | 2.19 |
| A11742 | KEQ67618.1; RNA polymerase I-specific transcription initiation factor RRN3 [ *Aureobasidium* *melanogenum* CBS 110374] | 0 | 2.88 | 3.8 | 3.07 | 2.27 |
| A11766 | OBW65758.1; Xylose isomerase-like protein [ *Aureobasidium* *pullulans*] | 0 | 2.5 | 1.96 | 2.74 | 2.42 |
| A11770 | KEQ67591.1; hypothetical protein M437DRAFT_80235 [ *Aureobasidium* *melanogenum* CBS 110374] | 0 | 1.13 | 1.62 | 1.45 | 1.15 |
| A11778 | XP_013344259.1; hypothetical protein AUEXF2481DRAFT_65049 [ *Aureobasidium* *subglaciale* EXF-2481] | 0 | 1.26 | 1.07 | 1.06 | 0.76 |
| A11780 | XP_013344205.1; hypothetical protein AUEXF2481DRAFT_39565 [ *Aureobasidium* *subglaciale* EXF-2481] | 0 | 1.44 | 1.89 | 2.53 | 2.21 |
| A11786 | XP_013339730.1; hypothetical protein AUEXF2481DRAFT_33208 [ *Aureobasidium* *subglaciale* EXF-2481] | 0 | 0.97 | 0.94 | 1.26 | 0.84 |
| A11797 | XP_013339761.1; hypothetical protein AUEXF2481DRAFT_70061 [ *Aureobasidium* *subglaciale* EXF-2481] | 0 | 2.43 | 2.21 | 2.47 | 2.49 |
| A11855 | KEQ60361.1; hypothetical protein M437DRAFT_68269 [ *Aureobasidium* *melanogenum* CBS 110374] | 0 | 0.73 | 1.09 | 1.08 | 1.17 |
| A11856 | XP_013339414.1; hypothetical protein AUEXF2481DRAFT_83553 [ *Aureobasidium* *subglaciale* EXF-2481] | 0 | 1.45 | 1.97 | 2.53 | 2.28 |
| A11868 | KEQ60345.1; hypothetical protein M437DRAFT_54682 [ *Aureobasidium* *melanogenum* CBS 110374] | 0 | 3.99 | 2.22 | 3.96 | 3.2 |
| A11872 | KEQ60340.1; hypothetical protein M437DRAFT_68251 [ *Aureobasidium* *melanogenum* CBS 110374] | 0 | 1.34 | 1.02 | 0.84 | 1.86 |
| A11878 | XP_013344082.1; hypothetical protein AUEXF2481DRAFT_4730 [ *Aureobasidium* *subglaciale* EXF-2481] | 0 | 1.38 | 1.25 | 1.09 | 0.87 |
| A11893 | KEQ88440.1; hypothetical protein M438DRAFT_84060 [ *Aureobasidium* *pullulans* EXF-150] | 0 | 0.93 | 0.9 | 1.18 | 1.02 |
| A11905 | KEQ65293.1; hypothetical protein M437DRAFT_73230 [ *Aureobasidium* *melanogenum* CBS 110374] | 0 | 2.03 | 1.5 | 1.42 | 1.2 |
| A11942 | KEQ65513.1; hypothetical protein M437DRAFT_64113 [ *Aureobasidium* *melanogenum* CBS 110374] | 0 | 1.89 | 2.05 | 2.52 | 2.63 |
| A11950 | NA | 0 | 3.52 | 3.78 | 4.34 | 4.83 |
| A11951 | KEQ65531.1; MFS general substrate transporter [ *Aureobasidium* *melanogenum* CBS 110374] | 0 | 3.24 | 4.29 | 4.6 | 4.95 |
| A11961 | KEQ65380.1; isochorismatase [ *Aureobasidium* *melanogenum* CBS 110374] | 0 | 2.32 | 1.28 | 1.63 | 1.79 |
| A11985 | KEQ60753.1; D123-domain-containing protein [ *Aureobasidium* *melanogenum* CBS 110374] | 0 | 2.11 | 1.65 | 1.82 | 2.57 |
| A11987 | KEQ60755.1; hypothetical protein M437DRAFT_53660 [ *Aureobasidium* *melanogenum* CBS 110374] | 0 | 1.31 | 1.63 | 1.25 | 1.24 |
| A11999 | KEQ60766.1; alpha/beta-hydrolase [ *Aureobasidium* *melanogenum* CBS 110374] | 0 | 1.46 | 2.01 | 1.83 | 1.45 |
| A12013 | KEQ62139.1; Creatinase/aminopeptidase [ *Aureobasidium* *melanogenum* CBS 110374] | 0 | 3.37 | 3.33 | 3.04 | 2.03 |
| A12017 | KEQ60781.1; hypothetical protein M437DRAFT_77139 [ *Aureobasidium* *melanogenum* CBS 110374] | 0 | 1.49 | 0.66 | 0.91 | 0.99 |
| A12029 | KEQ60790.1; Hydroxyisourate hydrolase [ *Aureobasidium* *melanogenum* CBS 110374] | 0 | 1.15 | 0.95 | 0.97 | 1.36 |
| A12031 | KEQ65481.1; hypothetical protein M437DRAFT_82443 [ *Aureobasidium* *melanogenum* CBS 110374] | 0 | 1.89 | 2.88 | 2.29 | 2.25 |
| A12056 | XP_013347429.1; hypothetical protein AUEXF2481DRAFT_36112 [ *Aureobasidium* *subglaciale* EXF-2481] | 0 | 3.18 | 2.94 | 2.83 | 2.49 |
| A12063 | KEQ86091.1; cytochrome P450 [ *Aureobasidium* *pullulans* EXF-150] | 0 | 2.08 | 1.77 | 2.06 | 2.06 |
| A12090 | KEQ62130.1; hypothetical protein M437DRAFT_29451, partial [ *Aureobasidium* *melanogenum* CBS 110374] | 0 | 1.61 | 0.88 | 1.5 | 1.13 |
| A12106 | KEQ65413.1; hypothetical protein M437DRAFT_42595 [ *Aureobasidium* *melanogenum* CBS 110374] | 0 | 2.14 | 2.12 | 1.54 | 1.97 |
| A12111 | KEQ65408.1; DUF1708-domain-containing protein [ *Aureobasidium* *melanogenum* CBS 110374] | 0 | 1 | 1.84 | 1.44 | 1.68 |
| A12113 | KEQ65407.1; hypothetical protein M437DRAFT_42342 [ *Aureobasidium* *melanogenum* CBS 110374] | 0 | 2.51 | 2.85 | 3.95 | 3.83 |
| A12119 | KEQ65400.1; amino acid transporter [ *Aureobasidium* *melanogenum* CBS 110374] | 0 | 2.71 | 3.41 | 2.88 | 3.17 |
| A12130 | KEQ57783.1; hypothetical protein M437DRAFT_89167 [ *Aureobasidium* *melanogenum* CBS 110374] | 0 | 1.71 | 2.2 | 2.31 | 2.13 |
| A12158 | XP_013431028.1; hypothetical protein M436DRAFT_60549 [ *Aureobasidium* *namibiae* CBS 147.97] | 0 | 1.79 | 2.23 | 2.68 | 2.93 |
| A12161 | KEQ60720.1; hypothetical protein M437DRAFT_77085 [ *Aureobasidium* *melanogenum* CBS 110374] | 0 | 1.66 | 1.28 | 1.73 | 1.18 |
| A12182 | KEQ60700.1; LamB/YcsF [ *Aureobasidium* *melanogenum* CBS 110374] | 0 | 3.44 | 1.58 | 2.47 | 2.12 |
| A12183 | KEQ60699.1; hypothetical protein M437DRAFT_67874 [ *Aureobasidium* *melanogenum* CBS 110374] | 0 | 2.62 | 1.36 | 2.2 | 2.01 |
| A12184 | KEQ60698.1; hypothetical protein M437DRAFT_86414 [ *Aureobasidium* *melanogenum* CBS 110374] | 0 | 3.73 | 2.99 | 2.66 | 2.73 |
| A12193 | KEQ60690.1; putative GABA permease [ *Aureobasidium* *melanogenum* CBS 110374] | 0 | 1.92 | 1.77 | 2.26 | 2.22 |
| A12230 | KEQ60832.1; putative sugar transporter [ *Aureobasidium* *melanogenum* CBS 110374] | 0 | 1.19 | 1.59 | 1.38 | 1.82 |
| A12241 | KEQ86007.1; hypothetical protein M438DRAFT_316185 [ *Aureobasidium* *pullulans* EXF-150] | 0 | 1.03 | 1.11 | 1.1 | 0.65 |
| A12250 | KEQ86018.1; WD40 repeat-like protein [ *Aureobasidium* *pullulans* EXF-150] | 0 | 2.24 | 3.1 | 3.06 | 2.36 |
| A12252 | XP_013431168.1; peroxisomal membrane protein pex16 [ *Aureobasidium* *namibiae* CBS 147.97] | 0 | 0.93 | 0.92 | 1.26 | 1.07 |
| A12287 | KEQ62215.1; hypothetical protein M437DRAFT_66645 [ *Aureobasidium* *melanogenum* CBS 110374] | 0 | 3.24 | 2.61 | 1.6 | 2.8 |
| A12321 | KEQ62314.1; hypothetical protein M437DRAFT_50325 [ *Aureobasidium* *melanogenum* CBS 110374] | 0 | 1.39 | 0.87 | 0.92 | 0.72 |
| A12323 | XP_013341351.1; hypothetical protein AUEXF2481DRAFT_42521 [ *Aureobasidium* *subglaciale* EXF-2481] | 0 | 1.81 | 1.81 | 1.61 | 1.67 |
| A12328 | KEQ62308.1; hypothetical protein M437DRAFT_50169 [ *Aureobasidium* *melanogenum* CBS 110374] | 0 | 2.23 | 3.08 | 2.93 | 2.48 |
| A12348 | KEQ60681.1; DNA/RNA polymerase [ *Aureobasidium* *melanogenum* CBS 110374] | 0 | 1.19 | 1.35 | 1.73 | 1.36 |
| A12355 | KEQ60673.1; hypothetical protein M437DRAFT_53749 [ *Aureobasidium* *melanogenum* CBS 110374] | 0 | 2.43 | 2.48 | 2.09 | 2.13 |
| A12386 | KEQ60643.1; peroxisomal membrane protein-like protein [ *Aureobasidium* *melanogenum* CBS 110374] | 0 | 1.32 | 0.95 | 1.64 | 1.81 |
| A12406 | XP_013431191.1; hypothetical protein M436DRAFT_69742 [ *Aureobasidium* *namibiae* CBS 147.97] | 0 | 2.09 | 2.05 | 2.53 | 3.19 |
| A12413 | KEQ60794.1; hypothetical protein M437DRAFT_53463 [ *Aureobasidium* *melanogenum* CBS 110374] | 0 | 4.93 | 5.77 | 5.32 | 3.47 |
| A12414 | KEQ60793.1; hypothetical protein M437DRAFT_53874 [ *Aureobasidium* *melanogenum* CBS 110374] | 0 | 3.43 | 3.58 | 3.94 | 2.88 |
| A12415 | KEQ60792.1; hypothetical protein M437DRAFT_53724 [ *Aureobasidium* *melanogenum* CBS 110374] | 0 | 2.85 | 3.36 | 3.39 | 2.58 |
| A12416 | KEQ86113.1; 4-aminobutyrate aminotransferase [ *Aureobasidium* *pullulans* EXF-150] | 0 | 2.66 | 2.41 | 2.58 | 1.96 |
| A12445 | KEQ62098.1; 4-oxalocrotonate tautomerase [ *Aureobasidium* *melanogenum* CBS 110374] | 0 | 5.47 | 4.28 | 4.49 | 3.95 |
| A12447 | KEQ62293.1; hypothetical protein M437DRAFT_66713 [ *Aureobasidium* *melanogenum* CBS 110374] | 0 | 2.82 | 3.22 | 3.85 | 4.31 |
| A12457 | KEQ62097.1; cytochrome P450 [ *Aureobasidium* *melanogenum* CBS 110374] | 0 | 3.61 | 3.6 | 3.44 | 3.6 |
| A12485 | KEQ62160.1; hypothetical protein M437DRAFT_85065 [ *Aureobasidium* *melanogenum* CBS 110374] | 0 | 2.49 | 3.05 | 3.24 | 2.67 |
| A12495 | KEQ62153.1; MFS general substrate transporter [ *Aureobasidium* *melanogenum* CBS 110374] | 0 | 2.39 | 3.18 | 3.16 | 2.88 |
| A12501 | XP_013430895.1; hypothetical protein M436DRAFT_38891 [ *Aureobasidium* *namibiae* CBS 147.97] | 0 | 1.03 | 1.02 | 1.36 | 1.14 |
| A12508 | KEQ65521.1; hypothetical protein M437DRAFT_42981 [ *Aureobasidium* *melanogenum* CBS 110374] | 0 | 1.21 | 1.31 | 1.59 | 1.71 |
| A12519 | XP_013431082.1; cytidine deaminase-like protein [ *Aureobasidium* *namibiae* CBS 147.97] | 0 | 1.71 | 2.13 | 2.18 | 2.14 |
| A12530 | XP_013347409.1; hypothetical protein AUEXF2481DRAFT_36022 [ *Aureobasidium* *subglaciale* EXF-2481] | 0 | 1.11 | 0.93 | 1.02 | 0.79 |
| A12599 | OBW68624.1; hypothetical protein AUREO_013190 [ *Aureobasidium* *pullulans*] | 0 | 1.61 | 1.8 | 1.35 | 1.68 |
| A12602 | KEQ65246.1; small oligopeptide transporter [ *Aureobasidium* *melanogenum* CBS 110374] | 0 | 3.21 | 3.28 | 2 | 2.02 |
| A12641 | NA | 0 | 1.43 | 0.94 | 1.27 | 0.85 |
| A12670 | KEQ65183.1; drug resistance protein [ *Aureobasidium* *melanogenum* CBS 110374] | 0 | 1.34 | 1.55 | 1.66 | 1.84 |
| A12702 | KFY07532.1; hypothetical protein V492_07057 [Pseudogymnoascus sp. VKM F-4246] | 0 | 1.92 | 2.08 | 2.15 | 1.41 |
| A12710 | KEQ65138.1; hypothetical protein M437DRAFT_82114 [ *Aureobasidium* *melanogenum* CBS 110374] | 0 | 1.63 | 1.6 | 1.46 | 0.88 |
| A12723 | XP_013347330.1; hypothetical protein AUEXF2481DRAFT_26151 [ *Aureobasidium* *subglaciale* EXF-2481] | 0 | 1.15 | 1.11 | 1.4 | 1.75 |
| A12727 | KEQ65099.1; hypothetical protein M437DRAFT_41977 [ *Aureobasidium* *melanogenum* CBS 110374] | 0 | 1 | 1.18 | 1.13 | 1.04 |
| A12744 | KEQ65034.1; NAD(P)-binding protein [ *Aureobasidium* *melanogenum* CBS 110374] | 0 | 0.98 | 1.07 | 1.2 | 0.8 |
| A12762 | KEQ65056.1; hypothetical protein M437DRAFT_63713 [ *Aureobasidium* *melanogenum* CBS 110374] | 0 | 3.79 | 2.72 | 4.13 | 2.92 |
| A12774 | KEQ65068.1; MFS general substrate transporter [ *Aureobasidium* *melanogenum* CBS 110374] | 0 | 1.13 | 1.43 | 1.48 | 1.66 |
| A12807 | KEQ65130.1; norsolorinic acid reductase [ *Aureobasidium* *melanogenum* CBS 110374] | 0 | 2.21 | 2.65 | 2.25 | 1.58 |
| A12808 | KEQ65108.1; hypothetical protein M437DRAFT_63758 [ *Aureobasidium* *melanogenum* CBS 110374] | 0 | 1.33 | 1.66 | 1.31 | 1.01 |
| A12816 | KEQ65110.1; NAD(P)-binding protein [ *Aureobasidium* *melanogenum* CBS 110374] | 0 | 0.88 | 1.14 | 1.03 | 0.8 |
| A12830 | XP_013431265.1; Arabinanase/levansucrase/invertase [ *Aureobasidium* *namibiae* CBS 147.97] | 0 | 3.69 | 3.66 | 3.21 | 3.79 |
| A12844 | KEQ65010.1; hypothetical protein M437DRAFT_42508 [ *Aureobasidium* *melanogenum* CBS 110374] | 0 | 1.71 | 1.77 | 1.39 | 1.69 |
| A12858 | XP_013347525.1; hypothetical protein AUEXF2481DRAFT_36590 [ *Aureobasidium* *subglaciale* EXF-2481] | 0 | 1.91 | 1.13 | 0.82 | 1.01 |
| A12873 | KEQ58762.1; amidase signature enzyme [ *Aureobasidium* *melanogenum* CBS 110374] | 0 | 2.33 | 1.85 | 1.81 | 1.66 |
| A12886 | KEQ58750.1; hypothetical protein M437DRAFT_78763 [ *Aureobasidium* *melanogenum* CBS 110374] | 0 | 0.86 | 1.16 | 1.44 | 1.14 |
| A12887 | XP_013349085.1; hypothetical protein AUEXF2481DRAFT_143886 [ *Aureobasidium* *subglaciale* EXF-2481] | 0 | 2.64 | 3.34 | 3.22 | 3.32 |
| A12889 | KEQ58747.1; hypothetical protein M437DRAFT_88284 [ *Aureobasidium* *melanogenum* CBS 110374] | 0 | 0.8 | 0.66 | 0.92 | 1.11 |
| A12908 | OBW67276.1; ATP-dependent metallopeptidase Hfl [ *Aureobasidium* *pullulans*] | 0 | 2.24 | 2.98 | 3.25 | 2.39 |
| A12912 | KEQ59846.1; putative iron-sulfur cluster assembly associated protein Nar1 [ *Aureobasidium* *melanogenum* CBS 110374] | 0 | 0.93 | 1.3 | 0.99 | 1.16 |
| A12936 | OCK94106.1; putative cytochrome P450 [Cenococcum geophilum 1.58] | 0 | 2.59 | 2.71 | 2.42 | 2.69 |
| A12943 | KEQ59876.1; hypothetical protein M437DRAFT_77964 [ *Aureobasidium* *melanogenum* CBS 110374] | 0 | 2.92 | 2.91 | 3.46 | 2.92 |
| A12949 | KEQ59882.1; tyrosine protein phosphatase 1 [ *Aureobasidium* *melanogenum* CBS 110374] | 0 | 2.01 | 2.42 | 2.49 | 2.11 |
| A12952 | XP_013349311.1; hypothetical protein AUEXF2481DRAFT_93779 [ *Aureobasidium* *subglaciale* EXF-2481] | 0 | 1.31 | 1.51 | 1.84 | 1.54 |
| A12957 | KEQ58813.1; RIO1-domain-containing protein [ *Aureobasidium* *melanogenum* CBS 110374] | 0 | 1.8 | 2.47 | 2.78 | 2.1 |
| A12970 | KEQ58800.1; S-adenosyl-L-methionine-dependent methyltransferase [ *Aureobasidium* *melanogenum* CBS 110374] | 0 | 1.46 | 1.6 | 1.48 | 1.77 |
| A13050 | KEQ59802.1; exoribonuclease family protein [ *Aureobasidium* *melanogenum* CBS 110374] | 0 | 1.17 | 1.07 | 1.07 | 1.19 |
| A13051 | KEQ59801.1; hypothetical protein M437DRAFT_12770, partial [ *Aureobasidium* *melanogenum* CBS 110374] | 0 | 1.38 | 1.51 | 1.54 | 1.92 |
| A13052 | XP_013349141.1; hypothetical protein AUEXF2481DRAFT_24933 [ *Aureobasidium* *subglaciale* EXF-2481] | 0 | 1.19 | 1.95 | 2.03 | 2.1 |
| A13053 | KEQ59799.1; ferric-chelate reductase [ *Aureobasidium* *melanogenum* CBS 110374] | 0 | 0.82 | 1.18 | 1.06 | 0.93 |
| A13070 | KEQ59784.1; p60 domain-containing protein [ *Aureobasidium* *melanogenum* CBS 110374] | 0 | 2.39 | 3.45 | 3.18 | 2.54 |
| A13088 | XP_013349370.1; hypothetical protein AUEXF2481DRAFT_165029 [ *Aureobasidium* *subglaciale* EXF-2481] | 0 | 0.64 | 0.93 | 1.02 | 0.95 |
| A13125 | KEQ58359.1; propionyl-CoA carboxylase [ *Aureobasidium* *melanogenum* CBS 110374] | 0 | 1.38 | 1.35 | 1.95 | 1.88 |
| A13187 | KEQ62674.1; hypothetical protein M437DRAFT_48932 [ *Aureobasidium* *melanogenum* CBS 110374] | 0 | 1.94 | 2.45 | 2.81 | 2.24 |
| A13203 | OBW67154.1; Uncharacterized protein AUREO_027710 [ *Aureobasidium* *pullulans*] | 0 | 1.35 | 1.77 | 1.3 | 1.7 |
| A13231 | KEQ62743.1; hypothetical protein M437DRAFT_65997 [ *Aureobasidium* *melanogenum* CBS 110374] | 0 | 1.88 | 1.99 | 2.22 | 2.31 |
| A13243 | KEQ62754.1; hypothetical protein M437DRAFT_66004 [ *Aureobasidium* *melanogenum* CBS 110374] | 0 | 1.17 | 1.53 | 1.43 | 1.22 |
| A13253 | XP_013348954.1; hypothetical protein AUEXF2481DRAFT_450 [ *Aureobasidium* *subglaciale* EXF-2481] | 0 | 0.89 | 0.73 | 1.17 | 1.04 |
| A13256 | KEQ62768.1; acid protease [ *Aureobasidium* *melanogenum* CBS 110374] | 0 | 0.82 | 1.06 | 0.87 | 0.72 |
| A13270 | KEQ62779.1; hypothetical protein M437DRAFT_48791 [ *Aureobasidium* *melanogenum* CBS 110374] | 0 | 0.78 | 0.91 | 1.04 | 0.91 |
| A13287 | KEQ62795.1; Xanthine/uracil permease [ *Aureobasidium* *melanogenum* CBS 110374] | 0 | 3.83 | 4.34 | 4.02 | 4.43 |
| A13290 | AIZ77451.1; DNA-binding protein creA [ *Aureobasidium* *pullulans*] | 0 | 0.7 | 0.6 | 0.73 | 1.03 |
| A13293 | KEQ62803.1; MOSC-domain-containing protein [ *Aureobasidium* *melanogenum* CBS 110374] | 0 | 2.27 | 2.38 | 2.71 | 2.26 |
| A13350 | XP_013348929.1; hypothetical protein AUEXF2481DRAFT_24751 [ *Aureobasidium* *subglaciale* EXF-2481] | 0 | 1 | 0.98 | 0.98 | 1.01 |
| A13351 | KEQ62857.1; glucose 1-dehydrogenase [ *Aureobasidium* *melanogenum* CBS 110374] | 0 | 2.67 | 2.56 | 2.56 | 2.23 |
| A13352 | KEQ62858.1; AAT-domain-containing protein [ *Aureobasidium* *melanogenum* CBS 110374] | 0 | 4.98 | 4.25 | 4.27 | 4.08 |
| A13369 | OQV11204.1; hypothetical protein CLAIMM_15073 [Cladophialophora immunda] | 0 | 1.71 | 1.44 | 1.73 | 1.42 |
| A13377 | KEQ62882.1; citrate synthase [ *Aureobasidium* *melanogenum* CBS 110374] | 0 | 2.59 | 1.99 | 3.28 | 3.17 |
| A13389 | KEQ62894.1; putative FRE ferric reductase-like transmembrane component [ *Aureobasidium* *melanogenum* CBS 110374] | 0 | 3.32 | 3.37 | 4.06 | 4.62 |
| A13487 | KEQ62908.1; phosphatidylethanolamine N-methyltransferase [ *Aureobasidium* *melanogenum* CBS 110374] | 0 | 3.11 | 3.19 | 3.62 | 3.44 |
| A13511 | KEQ60030.1; putative uridine/cytidine kinase [ *Aureobasidium* *melanogenum* CBS 110374] | 0 | 0.98 | 0.88 | 1.09 | 1.02 |
| A13520 | KEQ60038.1; hypothetical protein M437DRAFT_55701 [ *Aureobasidium* *melanogenum* CBS 110374] | 0 | 2.21 | 2.64 | 1.9 | 2.57 |
| A13522 | KEQ60040.1; hypothetical protein M437DRAFT_55449 [ *Aureobasidium* *melanogenum* CBS 110374] | 0 | 2.61 | 1.54 | 2.56 | 2.66 |
| A13544 | NA | 0 | 2.01 | 2.79 | 2.34 | 2.12 |
| A13559 | KEQ60005.1; general substrate transporter [ *Aureobasidium* *melanogenum* CBS 110374] | 0 | 1.16 | 1.74 | 1.6 | 1.97 |
| A13560 | KEQ60006.1; hypothetical protein M437DRAFT_87188 [ *Aureobasidium* *melanogenum* CBS 110374] | 0 | 1.71 | 1.19 | 1.68 | 1.91 |
| A13582 | KEQ59951.1; AAT family amino acid transporter [ *Aureobasidium* *melanogenum* CBS 110374] | 0 | 6.97 | 6.24 | 7.71 | 8.7 |
| A13593 | KEQ59960.1; hypothetical protein M437DRAFT_77727 [ *Aureobasidium* *melanogenum* CBS 110374] | 0 | 1.45 | 1.51 | 1.91 | 1.51 |
| A13599 | KEQ59967.1; DUF917-domain-containing protein [ *Aureobasidium* *melanogenum* CBS 110374] | 0 | 2.51 | 1.45 | 2.01 | 1.26 |
| A13608 | KEQ59930.1; heme peroxidase [ *Aureobasidium* *melanogenum* CBS 110374] | 0 | 2.22 | 1.42 | 2.32 | 3.12 |
| A13614 | KEQ81728.1; hypothetical protein M438DRAFT_337700 [ *Aureobasidium* *pullulans* EXF-150] | 0 | 3.14 | 2.03 | 3.37 | 3.69 |
| A13702 | OBW65016.1; Cutinase [ *Aureobasidium* *pullulans*] | 0 | 0.73 | 1.3 | 1.23 | 1.18 |
| A13744 | KEQ62443.1; hypothetical protein M437DRAFT_66311 [ *Aureobasidium* *melanogenum* CBS 110374] | 0 | 1.53 | 1.98 | 1.74 | 1.36 |
| A13769 | KEQ58773.1; ammonium transporter [ *Aureobasidium* *melanogenum* CBS 110374] | 0 | 2.69 | 2.85 | 2.99 | 3.16 |
| A13781 | KEQ62345.1; putative MFS transporter [ *Aureobasidium* *melanogenum* CBS 110374] | 0 | 1.04 | 0.86 | 0.97 | 0.86 |
| A13782 | KEQ62344.1; hypothetical protein M437DRAFT_84687 [ *Aureobasidium* *melanogenum* CBS 110374] | 0 | 2.39 | 2.36 | 2.93 | 2.86 |
| A13786 | XP_013429732.1; hypothetical protein M436DRAFT_70752 [ *Aureobasidium* *namibiae* CBS 147.97] | 0 | 1.1 | 0.86 | 1.31 | 1.09 |
| A13800 | XP_013427810.1; MFS sugar transporter-like protein [ *Aureobasidium* *namibiae* CBS 147.97] | 0 | 1.71 | 1.83 | 2.17 | 2.01 |
| A13838 | KEQ62272.1; 3-dehydroshikimate dehydratase [ *Aureobasidium* *melanogenum* CBS 110374] | 0 | 2.44 | 1.81 | 2.54 | 2.53 |
| A13842 | XP_013430925.1; hypothetical protein M436DRAFT_39042 [ *Aureobasidium* *namibiae* CBS 147.97] | 0 | 1.47 | 1.41 | 1.83 | 1.4 |
| A13852 | KEQ62289.1; Sodium/hydrogen exchanger [ *Aureobasidium* *melanogenum* CBS 110374] | 0 | 1.41 | 1.37 | 1.15 | 0.79 |
| A13855 | KEQ85918.1; hypothetical protein M438DRAFT_187012 [ *Aureobasidium* *pullulans* EXF-150] | 0 | 2.88 | 2.62 | 3.36 | 3.55 |
| A13868 | KEQ62231.1; kinase-like protein [ *Aureobasidium* *melanogenum* CBS 110374] | 0 | 2.61 | 3.18 | 3.81 | 4.14 |
| A13874 | XP_013341127.1; hypothetical protein AUEXF2481DRAFT_31908 [ *Aureobasidium* *subglaciale* EXF-2481] | 0 | 1.66 | 2.47 | 2.49 | 2.42 |
| A13875 | KEQ62221.1; hypothetical protein M437DRAFT_50182 [ *Aureobasidium* *melanogenum* CBS 110374] | 0 | 1.35 | 0.67 | 0.97 | 0.91 |
| A13902 | KEQ85956.1; hypothetical protein M438DRAFT_333894 [ *Aureobasidium* *pullulans* EXF-150] | 0 | 1.59 | 1.45 | 1.5 | 1.42 |
| A13913 | KEQ85794.1; hypothetical protein M438DRAFT_344240 [ *Aureobasidium* *pullulans* EXF-150] | 0 | 1.61 | 0.99 | 0.68 | 0.82 |
| A13923 | KEQ86038.1; alcohol oxidase [ *Aureobasidium* *pullulans* EXF-150] | 0 | 1.26 | 1.88 | 1.94 | 1.73 |
| A13929 | XP_013430648.1; hypothetical protein M436DRAFT_37493 [ *Aureobasidium* *namibiae* CBS 147.97] | 0 | 1 | 1.2 | 1 | 0.83 |
| A13988 | XP_013341255.1; hypothetical protein AUEXF2481DRAFT_415234 [ *Aureobasidium* *subglaciale* EXF-2481] | 0 | 1.26 | 1.45 | 1.6 | 1.62 |
| A13996 | KEQ57783.1; hypothetical protein M437DRAFT_89167 [ *Aureobasidium* *melanogenum* CBS 110374] | 0 | 1.87 | 2.79 | 2.07 | 2.82 |
| A14030 | XP_013341144.1; hypothetical protein AUEXF2481DRAFT_31998 [ *Aureobasidium* *subglaciale* EXF-2481] | 0 | 2.25 | 2.61 | 2.79 | 3.1 |
| A14043 | XP_013341196.1; hypothetical protein AUEXF2481DRAFT_68290 [ *Aureobasidium* *subglaciale* EXF-2481] | 0 | 2.4 | 2.54 | 2.85 | 3.21 |
| A14049 | KEQ57802.1; hypothetical protein M437DRAFT_60892 [ *Aureobasidium* *melanogenum* CBS 110374] | 0 | 1.16 | 1.06 | 1.59 | 1.34 |
| A14082 | KEQ65505.1; WD40 repeat-like protein [ *Aureobasidium* *melanogenum* CBS 110374] | 0 | 1.22 | 0.74 | 1.3 | 1.16 |
| A14097 | KEQ65531.1; MFS general substrate transporter [ *Aureobasidium* *melanogenum* CBS 110374] | 0 | 0.98 | 0.81 | 0.93 | 1.3 |
| A14131 | KEQ86166.1; DUF1708-domain-containing protein [ *Aureobasidium* *pullulans* EXF-150] | 0 | 1.54 | 2.19 | 2.09 | 2.19 |
| A14154 | XP_013430754.1; hypothetical protein M436DRAFT_38122 [ *Aureobasidium* *namibiae* CBS 147.97] | 0 | 2.48 | 2.26 | 2.6 | 2.64 |
| A14160 | KEQ86113.1; 4-aminobutyrate aminotransferase [ *Aureobasidium* *pullulans* EXF-150] | 0 | 1.67 | 1.55 | 1.91 | 1.31 |
| A14194 | KEQ86075.1; NAD(P)-binding protein [ *Aureobasidium* *pullulans*] | 0 | 1.26 | 1.43 | 1.21 | 0.91 |
| A14198 | KEQ65472.1; hypothetical protein M437DRAFT_73390 [ *Aureobasidium* *melanogenum* CBS 110374] | 0 | 3.76 | 4.67 | 5.05 | 4.34 |
| A14199 | XP_013431209.1; putative L-fucose-proton symporter [ *Aureobasidium* *namibiae* CBS 147.97] | 0 | 1.86 | 2.7 | 2.28 | 2.65 |
| A14203 | KEQ65479.1; methionine aminopeptidase [ *Aureobasidium* *melanogenum* CBS 110374] | 0 | 1.31 | 1.4 | 1.13 | 1.29 |
| A14207 | XP_013431394.1; alpha-amylase [ *Aureobasidium* *namibiae* CBS 147.97] | 0 | 1.14 | 1.57 | 1.56 | 1.38 |
| A14208 | XP_013347289.1; hypothetical protein AUEXF2481DRAFT_25994 [ *Aureobasidium* *subglaciale* EXF-2481] | 0 | 0.59 | 1.09 | 0.91 | 0.9 |
| A14217 | XP_013431065.1; hypothetical protein M436DRAFT_60753 [ *Aureobasidium* *namibiae* CBS 147.97] | 0 | 1.21 | 1.02 | 0.97 | 1.89 |
| A14224 | KEQ86143.1; hypothetical protein M438DRAFT_391097 [ *Aureobasidium* *pullulans* EXF-150] | 0 | 1.22 | 1.28 | 1.54 | 1.32 |
| A14226 | XP_013431047.1; hypothetical protein M436DRAFT_60645 [ *Aureobasidium* *namibiae* CBS 147.97] | 0 | 2.94 | 1.57 | 2.29 | 3.28 |
| A14261 | KEQ78905.1; hypothetical protein M438DRAFT_378559 [ *Aureobasidium* *pullulans* EXF-150] | 0 | 0.92 | 1.1 | 1.44 | 1.33 |
| A14263 | KEQ60753.1; D123-domain-containing protein [ *Aureobasidium* *melanogenum* CBS 110374] | 0 | 2.76 | 2.76 | 2.91 | 3.33 |
| A14264 | KEQ78908.1; hypothetical protein M438DRAFT_155761 [ *Aureobasidium* *pullulans* EXF-150] | 0 | 1.58 | 1.4 | 1.4 | 1.72 |
| A14265 | XP_013431191.1; hypothetical protein M436DRAFT_69742 [ *Aureobasidium* *namibiae* CBS 147.97] | 0 | 2.16 | 1.94 | 2.37 | 2.73 |
| A14275 | KEQ78919.1; hypothetical protein M438DRAFT_369865 [ *Aureobasidium* *pullulans* EXF-150] | 0 | 1.2 | 1.26 | 1.76 | 1.65 |
| A14281 | XP_013347321.1; hypothetical protein AUEXF2481DRAFT_26106 [ *Aureobasidium* *subglaciale* EXF-2481] | 0 | 1.91 | 1.6 | 1.68 | 1.61 |
| A14282 | KEQ60813.1; hypothetical protein M437DRAFT_77172 [ *Aureobasidium* *melanogenum* CBS 110374] | 0 | 2.15 | 2.13 | 2.43 | 1.86 |
| A14303 | XP_013431082.1; cytidine deaminase-like protein [ *Aureobasidium* *namibiae* CBS 147.97] | 0 | 1.93 | 2.35 | 2.33 | 2.03 |
| A14306 | OBW66996.1; Glycosyltransferase family 71 protein [ *Aureobasidium* *pullulans*] | 0 | 1.51 | 1.56 | 1.29 | 1.67 |
| A14317 | XP_013430761.1; hypothetical protein M436DRAFT_38158 [ *Aureobasidium* *namibiae* CBS 147.97] | 0 | 1.18 | 1.84 | 1.81 | 2.12 |
| A14320 | XP_013430827.1; hypothetical protein M436DRAFT_38510 [ *Aureobasidium* *namibiae* CBS 147.97] | 0 | 1.93 | 2.14 | 2.14 | 2.31 |
| A14332 | XP_013347330.1; hypothetical protein AUEXF2481DRAFT_26151 [ *Aureobasidium* *subglaciale* EXF-2481] | 0 | 0.8 | 0.95 | 1.11 | 1.3 |
| A14358 | KEQ65301.1; hypothetical protein M437DRAFT_82273 [ *Aureobasidium* *melanogenum* CBS 110374] | 0 | 2.77 | 3.26 | 4.03 | 2.97 |
| A14374 | KEQ65287.1; hypothetical protein M437DRAFT_42271 [ *Aureobasidium* *melanogenum* CBS 110374] | 0 | 2.55 | 3.75 | 3.39 | 2.81 |
| A14425 | XP_013347211.1; hypothetical protein AUEXF2481DRAFT_1844 [ *Aureobasidium* *subglaciale* EXF-2481] | 0 | 2.06 | 2.63 | 2.81 | 2.3 |
| A14451 | KEQ65209.1; hypothetical protein M437DRAFT_73146 [ *Aureobasidium* *melanogenum* CBS 110374] | 0 | 1.41 | 1.5 | 1.48 | 1 |
| A14460 | XP_013430787.1; hypothetical protein M436DRAFT_38292 [ *Aureobasidium* *namibiae* CBS 147.97] | 0 | 1.16 | 1.13 | 0.95 | 1.1 |
| A14507 | XP_013422807.1; hypothetical protein M436DRAFT_57912 [ *Aureobasidium* *namibiae* CBS 147.97] | 0 | 1.6 | 1.47 | 1.99 | 1.96 |
| A14521 | KEQ65052.1; retrograde regulation protein 2 [ *Aureobasidium* *melanogenum* CBS 110374] | 0 | 1.3 | 1.27 | 1.92 | 1.93 |
| A14537 | NA | 0 | 1.46 | 2.06 | 1.81 | 1.78 |
| A14546 | XP_013347368.1; hypothetical protein AUEXF2481DRAFT_26353 [ *Aureobasidium* *subglaciale* EXF-2481] | 0 | 2.19 | 2.05 | 2.62 | 2.49 |
| A14559 | OBW68968.1; Uncharacterized protein AUREO_009530 [ *Aureobasidium* *pullulans*] | 0 | 1.45 | 1.87 | 2.04 | 1.67 |
| A14568 | KEQ82337.1; Aldo/keto reductase [ *Aureobasidium* *pullulans* EXF-150] | 0 | 1.11 | 1.2 | 1.42 | 1.61 |
| A14578 | KEQ65113.1; UDP-glucose 4-epimerase [ *Aureobasidium* *melanogenum* CBS 110374] | 0 | 1.03 | 1.72 | 1.53 | 1.57 |
| A14618 | KEQ84979.1; alcohol oxidase [ *Aureobasidium* *pullulans* EXF-150] | 0 | 2.32 | 2.77 | 2.51 | 2.6 |
| A14632 | OBW64763.1; Uncharacterized protein AUREO_051690 [ *Aureobasidium* *pullulans*] | 0 | 1.47 | 1.76 | 1.35 | 1.02 |
| A14638 | XP_013340962.1; hypothetical protein AUEXF2481DRAFT_32156 [ *Aureobasidium* *subglaciale* EXF-2481] | 0 | 2.4 | 2.93 | 3.11 | 2.38 |
| A14639 | XP_013340939.1; glycosyltransferase family 2 protein [ *Aureobasidium* *subglaciale* EXF-2481] | 0 | 2.05 | 2.6 | 2.31 | 1.71 |
| A14640 | XP_013341036.1; hypothetical protein AUEXF2481DRAFT_82287 [ *Aureobasidium* *subglaciale* EXF-2481] | 0 | 1.74 | 1.95 | 1.87 | 1.92 |
| A14674 | KEQ62120.1; thiol methyltransferase [ *Aureobasidium* *melanogenum* CBS 110374] | 0 | 1.12 | 1.62 | 1.99 | 1.85 |
| A14699 | KEQ63648.1; heme peroxidase [ *Aureobasidium* *melanogenum* CBS 110374] | 0 | 1.52 | 1.24 | 1.2 | 0.89 |
| A14703 | OBW67245.1; Uncharacterized protein AUREO_026850 [ *Aureobasidium* *pullulans*] | 0 | 1.3 | 1.33 | 1.05 | 0.69 |
| A14704 | KEQ63643.1; proline oxidase Put1 [ *Aureobasidium* *melanogenum* CBS 110374] | 0 | 5.63 | 4.63 | 4.31 | 4.18 |
| A14773 | KEQ63402.1; hypothetical protein M437DRAFT_46847 [ *Aureobasidium* *melanogenum* CBS 110374] | 0 | 1.25 | 1.72 | 2.09 | 1.72 |
| A14815 | XP_013424761.1; hypothetical protein M436DRAFT_12673, partial [ *Aureobasidium* *namibiae* CBS 147.97] | 0 | 1.41 | 1.6 | 1.7 | 1.6 |
| A14816 | KEQ63443.1; AAA-domain-containing protein [ *Aureobasidium* *melanogenum* CBS 110374] | 0 | 2.7 | 2.17 | 3.1 | 2.72 |
| A14832 | OBW68768.1; hypothetical protein AUREO_011590 [ *Aureobasidium* *pullulans*] | 0 | 1.02 | 1.18 | 1.49 | 1.41 |
| A14838 | KEQ63222.1; hypothetical protein M437DRAFT_75105 [ *Aureobasidium* *melanogenum* CBS 110374] | 0 | 1.65 | 1.62 | 1.52 | 1.57 |
| A14839 | KEQ63223.1; putative high affinity copper protein [ *Aureobasidium* *melanogenum* CBS 110374] | 0 | 2.54 | 2.97 | 2.71 | 2.52 |
| A14856 | KEQ63242.1; aldehyde dehydrogenase [ *Aureobasidium* *melanogenum* CBS 110374] | 0 | 0.93 | 0.71 | 1.13 | 1.15 |
| A14864 | KEQ63250.1; MFS transporter [ *Aureobasidium* *melanogenum* CBS 110374] | 0 | 3.7 | 2.2 | 2.25 | 1.77 |
| A14870 | KEQ63255.1; beta-lactamase/transpeptidase-like protein [ *Aureobasidium* *melanogenum* CBS 110374] | 0 | 2.29 | 2.33 | 2.41 | 2.91 |
| A14916 | NA | 0 | 1.44 | 1.46 | 0.87 | 1.5 |
| A14959 | XP_013425485.1; DUF453-domain-containing protein [ *Aureobasidium* *namibiae* CBS 147.97] | 0 | 2.27 | 2.02 | 1.92 | 2.52 |
| A14967 | KEQ63541.1; MFS general substrate transporter [ *Aureobasidium* *melanogenum* CBS 110374] | 0 | 1.8 | 1.87 | 1.21 | 1.17 |
| A14968 | KEQ63542.1; glutaminase A [ *Aureobasidium* *melanogenum* CBS 110374] | 0 | 2.71 | 2.97 | 3.44 | 3.09 |
| A14984 | KEQ63362.1; hypothetical protein M437DRAFT_83760 [ *Aureobasidium* *melanogenum* CBS 110374] | 0 | 1.71 | 1.9 | 1.48 | 1.97 |
| A14993 | KEQ61293.1; hypothetical protein M437DRAFT_51844 [ *Aureobasidium* *melanogenum* CBS 110374] | 0 | 1.97 | 1.19 | 1.58 | 2.85 |
| A15004 | KEQ79955.1; alpha/beta-hydrolase [ *Aureobasidium* *pullulans* EXF-150] | 0 | 1.16 | 1.57 | 1.24 | 1.09 |
| A15028 | KEQ61381.1; retinal dehydrogenase 2 [ *Aureobasidium* *melanogenum* CBS 110374] | 0 | 2.52 | 1.56 | 2.28 | 2.22 |
| A15057 | KEQ87275.1; hypothetical protein M438DRAFT_268618 [ *Aureobasidium* *pullulans* EXF-150] | 0 | 0.97 | 1.46 | 1.03 | 1.3 |
| A15081 | KEQ61433.1; hypothetical protein M437DRAFT_52266 [ *Aureobasidium* *melanogenum* CBS 110374] | 0 | 1.85 | 1.46 | 1.88 | 2.29 |
| A15094 | KEQ61448.1; P-loop containing nucleoside triphosphate hydrolase protein [ *Aureobasidium* *melanogenum* CBS 110374] | 0 | 2.67 | 1.69 | 1.26 | 2.18 |
| A15130 | KEQ61480.1; MFS phospholipid transporter Git1 [ *Aureobasidium* *melanogenum* CBS 110374] | 0 | 1.41 | 1.26 | 1.43 | 1.29 |
| A15143 | KEQ61305.1; hypothetical protein M437DRAFT_67260 [ *Aureobasidium* *melanogenum* CBS 110374] | 0 | 2.06 | 2.3 | 1.96 | 1.47 |
| A15154 | KEQ61318.1; putative sugar transporter [ *Aureobasidium* *melanogenum* CBS 110374] | 0 | 1.09 | 1.18 | 0.82 | 0.65 |
| A15159 | KEQ61324.1; fructosyl amino acid oxidase [ *Aureobasidium* *melanogenum* CBS 110374] | 0 | 1.26 | 1.32 | 1.45 | 1.34 |
| A15162 | KEQ61328.1; alpha/beta-hydrolase [ *Aureobasidium* *melanogenum* CBS 110374] | 0 | 4.41 | 3.56 | 3.87 | 3.56 |
| A15163 | OBW66611.1; Uncharacterized protein AUREO_033230 [ *Aureobasidium* *pullulans*] | 0 | 2.05 | 1.86 | 1.8 | 1.91 |
| A15200 | XP_013342710.1; hypothetical protein AUEXF2481DRAFT_98895 [ *Aureobasidium* *subglaciale* EXF-2481] | 0 | 1.22 | 1.14 | 1.09 | 1.26 |
| A15205 | KEQ80022.1; aquaporin-like protein [ *Aureobasidium* *pullulans*] | 0 | 1.55 | 2.05 | 1.83 | 1.97 |
| A15221 | KEQ63450.1; hypothetical protein M437DRAFT_65423 [ *Aureobasidium* *melanogenum* CBS 110374] | 0 | 0.82 | 0.82 | 1.14 | 1.01 |
| A15224 | KEQ60088.1; hypothetical protein M437DRAFT_77527 [ *Aureobasidium* *melanogenum* CBS 110374] | 0 | 1 | 1.02 | 0.97 | 0.86 |
| A15241 | KEQ60104.1; Thiamin diphosphate-binding protein [ *Aureobasidium* *melanogenum* CBS 110374] | 0 | 1.56 | 1.51 | 2.21 | 1.79 |
| A15259 | KEQ60119.1; putative ABC transporter [ *Aureobasidium* *melanogenum* CBS 110374] | 0 | 2.09 | 1.63 | 2.03 | 2.9 |
| A15263 | KEQ60123.1; hypothetical protein M437DRAFT_54931 [ *Aureobasidium* *melanogenum* CBS 110374] | 0 | 4.75 | 4.25 | 4.73 | 4.46 |
| A15280 | KEQ60139.1; galactose oxidase [ *Aureobasidium* *melanogenum* CBS 110374] | 0 | 0.96 | 1.06 | 0.69 | 0.68 |
| A15313 | KEQ60173.1; sorbitol dehydrogenase-like protein [ *Aureobasidium* *melanogenum* CBS 110374] | 0 | 2.4 | 2.24 | 3.41 | 3.08 |
| A15328 | KEQ59254.1; hypothetical protein M437DRAFT_57306 [ *Aureobasidium* *melanogenum* CBS 110374] | 0 | 0.94 | 0.99 | 1.03 | 1.1 |
| A15346 | KEQ59274.1; hypothetical protein M437DRAFT_78399 [ *Aureobasidium* *melanogenum* CBS 110374] | 0 | 1.24 | 1.44 | 1.48 | 1.45 |
| A15348 | KEQ59277.1; acyl-CoA dehydrogenase NM domain-like protein [ *Aureobasidium* *melanogenum* CBS 110374] | 0 | 1.08 | 0.82 | 1.4 | 1.2 |
| A15365 | KEQ59294.1; OPT family small oligopeptide transporter [ *Aureobasidium* *melanogenum* CBS 110374] | 0 | 3.12 | 2.93 | 2.84 | 3.33 |
| A15379 | KEQ59308.1; hypothetical protein M437DRAFT_57525 [ *Aureobasidium* *melanogenum* CBS 110374] | 0 | 2.87 | 3.23 | 2.87 | 2.6 |
| A15392 | KEQ87057.1; hypothetical protein M438DRAFT_313947 [ *Aureobasidium* *pullulans* EXF-150] | 0 | 2.44 | 3.84 | 4.52 | 4.18 |
| A15400 | KEQ60182.1; alpha/beta-hydrolase [ *Aureobasidium* *melanogenum* CBS 110374] | 0 | 1.58 | 1.65 | 2.04 | 1.31 |
| A15429 | KEQ60216.1; hypothetical protein M437DRAFT_68486 [ *Aureobasidium* *melanogenum* CBS 110374] | 0 | 2.12 | 2.79 | 2.81 | 3.57 |
| A15437 | KEQ60207.1; hypothetical protein M437DRAFT_55236 [ *Aureobasidium* *melanogenum* CBS 110374] | 0 | 1.11 | 1.4 | 1.6 | 1.38 |
| A15440 | KEQ60204.1; hypothetical protein M437DRAFT_55038, partial [ *Aureobasidium* *melanogenum* CBS 110374] | 0 | 1.61 | 2.21 | 2.44 | 2.25 |
| A15448 | XP_013346677.1; hypothetical protein AUEXF2481DRAFT_2494 [ *Aureobasidium* *subglaciale* EXF-2481] | 0 | 1.45 | 2.17 | 2.5 | 2.28 |
| A15450 | KEQ59241.1; hypothetical protein M437DRAFT_57313 [ *Aureobasidium* *melanogenum* CBS 110374] | 0 | 4.15 | 3.45 | 2.57 | 3.2 |
| A15451 | KEQ59240.1; inosine monophosphate dehydrogenase [ *Aureobasidium* *melanogenum* CBS 110374] | 0 | 3.52 | 3.04 | 2.8 | 3.07 |
| A15452 | XP_013346678.1; hypothetical protein AUEXF2481DRAFT_2499 [ *Aureobasidium* *subglaciale* EXF-2481] | 0 | 3.5 | 3.04 | 2.91 | 3.55 |
| A15453 | XP_013347099.1; hypothetical protein AUEXF2481DRAFT_494178 [ *Aureobasidium* *subglaciale* EXF-2481] | 0 | 2.57 | 2.54 | 2.52 | 2.47 |
| A15463 | KEQ59228.1; amino acid transporter [ *Aureobasidium* *melanogenum* CBS 110374] | 0 | 0.85 | 0.82 | 1.22 | 1.21 |
| A15464 | KEQ59227.1; hypothetical protein M437DRAFT_78365 [ *Aureobasidium* *melanogenum* CBS 110374] | 0 | 1.95 | 2.24 | 2.01 | 1.88 |
| A15469 | KEQ59224.1; amino acid permease [ *Aureobasidium* *melanogenum* CBS 110374] | 0 | 1.29 | 1.19 | 0.69 | 1.07 |
| A15470 | KEQ59223.1; 2-haloalkanoic acid dehalogenase [ *Aureobasidium* *melanogenum* CBS 110374] | 0 | 4.31 | 3.47 | 3.95 | 3.83 |
| A15490 | KEQ59205.1; general substrate transporter [ *Aureobasidium* *melanogenum* CBS 110374] | 0 | 1.44 | 2.21 | 2.31 | 2.49 |
| A15513 | KEQ59178.1; MFS general substrate transporter [ *Aureobasidium* *melanogenum* CBS 110374] | 0 | 1.63 | 1.93 | 1.6 | 1.32 |
| A15552 | XP_013422596.1; hypothetical protein M436DRAFT_58527 [ *Aureobasidium* *namibiae* CBS 147.97] | 0 | 1.13 | 1.29 | 1.17 | 1.61 |
| A15589 | XP_013348570.1; hypothetical protein AUEXF2481DRAFT_116296 [ *Aureobasidium* *subglaciale* EXF-2481] | 0 | 2.84 | 3.41 | 3.51 | 2.65 |
| A15597 | XP_013429738.1; cytochrome P450 [ *Aureobasidium* *namibiae* CBS 147.97] | 0 | 0.93 | 1.2 | 0.75 | 1.02 |
| A15606 | KEQ81791.1; general substrate transporter [ *Aureobasidium* *pullulans* EXF-150] | 0 | 1.08 | 1.38 | 1.24 | 1.75 |
| A15629 | XP_013348641.1; hypothetical protein AUEXF2481DRAFT_24500 [ *Aureobasidium* *subglaciale* EXF-2481] | 0 | 1.05 | 0.83 | 0.75 | 0.54 |
| A15658 | KEQ79329.1; hypothetical protein M438DRAFT_378332 [ *Aureobasidium* *pullulans*] | 0 | 1.55 | 1.91 | 2.47 | 2.52 |
| A15669 | XP_013340702.1; hypothetical protein AUEXF2481DRAFT_68962 [ *Aureobasidium* *subglaciale* EXF-2481] | 0 | 1.3 | 1.38 | 1.63 | 1.71 |
| A15687 | KEQ60033.1; MFS general substrate transporter [ *Aureobasidium* *melanogenum* CBS 110374] | 0 | 1.24 | 1.15 | 1.13 | 0.84 |
| A15691 | KEQ79279.1; hypothetical protein M438DRAFT_306465 [ *Aureobasidium* *pullulans*] | 0 | 3.11 | 3 | 2.44 | 3.23 |
| A15713 | OBW68792.1; Kinesin-like protein [ *Aureobasidium* *pullulans*] | 0 | 1.5 | 1.76 | 2.09 | 1.71 |
| A15720 | KEQ62984.1; rRNA processing protein Ipi1 [ *Aureobasidium* *melanogenum* CBS 110374] | 0 | 0.92 | 1.18 | 1.31 | 1.09 |
| A15757 | NA | 0 | 2.6 | 2.9 | 3.31 | 3.49 |
| A15759 | KEQ62942.1; NAD(P)-binding protein [ *Aureobasidium* *melanogenum* CBS 110374] | 0 | 1.43 | 1.63 | 2.04 | 1.63 |
| A15772 | KEQ62929.1; hypothetical protein M437DRAFT_32569, partial [ *Aureobasidium* *melanogenum* CBS 110374] | 0 | 2.65 | 2.44 | 1.86 | 2.27 |
| A15782 | XP_013348773.1; hypothetical protein AUEXF2481DRAFT_24621 [ *Aureobasidium* *subglaciale* EXF-2481] | 0 | 2.69 | 2.96 | 3.35 | 3.39 |
| A15789 | XP_013424379.1; hypothetical protein M436DRAFT_66578 [ *Aureobasidium* *namibiae* CBS 147.97] | 0 | 2.99 | 3.8 | 3.93 | 4.37 |
| A15791 | OBW68123.1; Clavaminate synthase-like protein [ *Aureobasidium* *pullulans*] | 0 | 2.54 | 2.07 | 2.3 | 2 |
| A15796 | KEQ88049.1; hypothetical protein M438DRAFT_313189 [ *Aureobasidium* *pullulans* EXF-150] | 0 | 2.92 | 3.13 | 2.65 | 2.16 |
| A15801 | OBW68114.1; Fimbrin [ *Aureobasidium* *pullulans*] | 0 | 1.69 | 1.26 | 1.75 | 1.27 |
| A15806 | KEQ62894.1; putative FRE ferric reductase-like transmembrane component [ *Aureobasidium* *melanogenum* CBS 110374] | 0 | 1.47 | 1.53 | 1.41 | 1.7 |
| A15866 | XP_013348965.1; hypothetical protein AUEXF2481DRAFT_34666 [ *Aureobasidium* *subglaciale* EXF-2481] | 0 | 1.81 | 2.4 | 2.51 | 2.23 |
| A15897 | KEQ87965.1; hypothetical protein M438DRAFT_313007 [ *Aureobasidium* *pullulans* EXF-150] | 0 | 1.62 | 1.65 | 1.35 | 1.29 |
| A15933 | KEQ87821.1; aorsin [ *Aureobasidium* *pullulans* EXF-150] | 0 | 1.29 | 0.69 | 0.81 | 0.95 |
| A15972 | KEQ87867.1; hypothetical protein M438DRAFT_290801, partial [ *Aureobasidium* *pullulans* EXF-150] | 0 | 1.12 | 1.59 | 1.43 | 1.19 |
| A15973 | XP_013349141.1; hypothetical protein AUEXF2481DRAFT_24933 [ *Aureobasidium* *subglaciale* EXF-2481] | 0 | 1.58 | 2 | 1.51 | 1.43 |
| A15974 | KEQ87869.1; hypothetical protein M438DRAFT_266211 [ *Aureobasidium* *pullulans* EXF-150] | 0 | 0.85 | 1.04 | 0.68 | 0.8 |
| A15975 | XP_013349138.1; hypothetical protein AUEXF2481DRAFT_146828 [ *Aureobasidium* *subglaciale* EXF-2481] | 0 | 1.91 | 2.59 | 2.64 | 2.88 |
| A15983 | OBW65016.1; Cutinase [ *Aureobasidium* *pullulans*] | 0 | 1.04 | 1.54 | 1.27 | 1.09 |
| A15990 | XP_013349120.1; hypothetical protein AUEXF2481DRAFT_24917 [ *Aureobasidium* *subglaciale* EXF-2481] | 0 | 3.14 | 1.98 | 1.52 | 1.88 |
| A16001 | XP_013348984.1; hypothetical protein AUEXF2481DRAFT_484 [ *Aureobasidium* *subglaciale* EXF-2481] | 0 | 2.31 | 3.26 | 2.6 | 2.32 |
| A16015 | XP_013349085.1; hypothetical protein AUEXF2481DRAFT_143886 [ *Aureobasidium* *subglaciale* EXF-2481] | 0 | 3.91 | 4.17 | 4.15 | 4.11 |
| A16025 | KEQ62833.1; hypothetical protein M437DRAFT_84527 [ *Aureobasidium* *melanogenum* CBS 110374] | 0 | 2.67 | 3.03 | 3.84 | 3.38 |
| A16044 | XP_013424450.1; hypothetical protein M436DRAFT_75529 [ *Aureobasidium* *namibiae* CBS 147.97] | 0 | 1.41 | 1 | 1.47 | 1.37 |
| A16051 | XP_013424296.1; hypothetical protein M436DRAFT_54130 [ *Aureobasidium* *namibiae* CBS 147.97] | 0 | 1.79 | 1.98 | 1.74 | 2.16 |
| A16068 | XP_013349360.1; hypothetical protein AUEXF2481DRAFT_25146 [ *Aureobasidium* *subglaciale* EXF-2481] | 0 | 2.61 | 2.18 | 2.47 | 2.28 |
| A16110 | XP_013349054.1; hypothetical protein AUEXF2481DRAFT_142532 [ *Aureobasidium* *subglaciale* EXF-2481] | 0 | 1.1 | 0.83 | 0.74 | 1.41 |
| A16155 | KEQ59723.1; hypothetical protein M437DRAFT_55892 [ *Aureobasidium* *melanogenum* CBS 110374] | 0 | 0.83 | 1.45 | 1.47 | 1.35 |
| A16162 | OBW68262.1; Mitochondrial Rho GTPase [ *Aureobasidium* *pullulans*] | 0 | 1.05 | 1.31 | 1.56 | 1.23 |
| A16184 | XP_013425799.1; natural resistance-associated macrophage protein [ *Aureobasidium* *namibiae* CBS 147.97] | 0 | 0.91 | 1.5 | 1.56 | 1.58 |
| A16196 | XP_013349247.1; hypothetical protein AUEXF2481DRAFT_93726 [ *Aureobasidium* *subglaciale* EXF-2481] | 0 | 1.05 | 0.72 | 0.81 | 0.77 |
| A16199 | KEQ87467.1; hypothetical protein M438DRAFT_371883 [ *Aureobasidium* *pullulans* EXF-150] | 0 | 2.04 | 3.01 | 3.16 | 2.57 |
| A16222 | KEQ58714.1; hypothetical protein M437DRAFT_58626 [ *Aureobasidium* *melanogenum* CBS 110374] | 0 | 1.18 | 0.93 | 0.9 | 1.07 |
| A16226 | NA | 0 | 2.98 | 3.66 | 3.61 | 2.77 |
| A16245 | KEQ85992.1; glycoside hydrolase [ *Aureobasidium* *pullulans* EXF-150] | 0 | 1.3 | 1.89 | 1.76 | 1.54 |
| A16246 | KEQ58359.1; propionyl-CoA carboxylase [ *Aureobasidium* *melanogenum* CBS 110374] | 0 | 0.86 | 1.48 | 1.39 | 1.58 |
| A16252 | OBW67155.1; Uncharacterized protein AUREO_027720 [ *Aureobasidium* *pullulans*] | 0 | 1.3 | 1.35 | 1.31 | 1.13 |
| A16253 | OBW67154.1; Uncharacterized protein AUREO_027710 [ *Aureobasidium* *pullulans*] | 0 | 2.17 | 3.37 | 3.68 | 3.81 |
| A16263 | KEQ58335.1; 5-methyltetrahydropteroyltriglutamate-homocysteine methyltransferase [ *Aureobasidium* *melanogenum* CBS 110374] | 0 | 1.35 | 0.77 | 1.08 | 1.18 |
| A16264 | XP_013423123.1; 3-phytase [ *Aureobasidium* *namibiae* CBS 147.97] | 0 | 1.04 | 2.14 | 1.62 | 2.02 |
| A16276 | KEQ58693.1; hypothetical protein M437DRAFT_88232 [ *Aureobasidium* *melanogenum* CBS 110374] | 0 | 4.44 | 3.49 | 3.4 | 4.36 |
| A16284 | XP_013348999.1; hypothetical protein AUEXF2481DRAFT_75567 [ *Aureobasidium* *subglaciale* EXF-2481] | 0 | 1.86 | 1.55 | 1.86 | 1.79 |
| A16286 | KEQ87380.1; PLP-dependent transferase [ *Aureobasidium* *pullulans* EXF-150] | 0 | 2.57 | 3.97 | 3.32 | 2.97 |
| A16304 | KEQ78581.1; MFS monocarboxylate transporter-like protein [ *Aureobasidium* *pullulans* EXF-150] | 0 | 1.72 | 1.63 | 1.43 | 1.07 |
| A16317 | XP_013429595.1; FAD/NAD(P)-binding domain-containing protein [ *Aureobasidium* *namibiae* CBS 147.97] | 0 | 2.28 | 1.94 | 1.66 | 2.99 |
| A16362 | KEQ81826.1; hypothetical protein M438DRAFT_399088 [ *Aureobasidium* *pullulans* EXF-150] | 0 | 2.97 | 3.83 | 3.63 | 3.5 |
| A16404 | ODM23038.1; hypothetical protein SI65_00627 [Aspergillus cristatus] | 0 | 1.51 | 2.2 | 2.62 | 2.13 |
| A16426 | OBW68243.1; Uncharacterized protein AUREO_016950 [ *Aureobasidium* *pullulans*] | 0 | 1.91 | 2.01 | 2.77 | 2.64 |
| A16428 | KEQ62993.1; nitrate reductase [ *Aureobasidium* *melanogenum* CBS 110374] | 0 | 2.61 | 3.38 | 2.82 | 2.23 |
| A16429 | KEQ62994.1; nitrite reductase [ *Aureobasidium* *melanogenum* CBS 110374] | 0 | 2.15 | 2.59 | 2.12 | 1.63 |
| A16437 | KEQ83589.1; mate family transporter [ *Aureobasidium* *pullulans* EXF-150] | 0 | 2.94 | 2.01 | 1.52 | 1.68 |
| A16438 | OBW67569.1; Serine/threonine-protein phosphatase 2A activator [ *Aureobasidium* *pullulans*] | 0 | 2.89 | 1.68 | 1.45 | 1.56 |
| A16441 | OBW67574.1; DUF1692-domain-containing protein [ *Aureobasidium* *pullulans*] | 0 | 1.7 | 1.13 | 1.77 | 1.13 |
| A16454 | XP_013341604.1; hypothetical protein AUEXF2481DRAFT_42443 [ *Aureobasidium* *subglaciale* EXF-2481] | 0 | 1.95 | 2.15 | 2.48 | 2.56 |
| A16467 | XP_013425520.1; hypothetical protein M436DRAFT_51489 [ *Aureobasidium* *namibiae* CBS 147.97] | 0 | 1.23 | 1.18 | 1.16 | 0.84 |
| A16477 | XP_013338811.1; hypothetical protein AUEXF2481DRAFT_575541 [ *Aureobasidium* *subglaciale* EXF-2481] | 0 | 1.03 | 1.03 | 1.46 | 1.39 |
| A16478 | KEQ83519.1; hypothetical protein M438DRAFT_397865 [ *Aureobasidium* *pullulans* EXF-150] | 0 | 1.6 | 2.22 | 2.04 | 1.83 |
| A16502 | KEQ60595.1; hypothetical protein M437DRAFT_54002 [ *Aureobasidium* *melanogenum* CBS 110374] | 0 | 1.56 | 2.4 | 2.15 | 1.98 |
| A16530 | XP_013346428.1; hypothetical protein AUEXF2481DRAFT_63320 [ *Aureobasidium* *subglaciale* EXF-2481] | 0 | 1.19 | 1.1 | 1.79 | 1.71 |
| A16550 | XP_013346547.1; hypothetical protein AUEXF2481DRAFT_703924 [ *Aureobasidium* *subglaciale* EXF-2481] | 0 | 1.38 | 1.26 | 1.27 | 1.02 |
| A16561 | OBW63900.1; Uncharacterized protein AUREO_060330 [ *Aureobasidium* *pullulans*] | 0 | 1.78 | 1.8 | 1.87 | 1.18 |
| A16571 | KEQ83438.1; putative efflux pump antibiotic resistance protein [ *Aureobasidium* *pullulans* EXF-150] | 0 | 2.29 | 1.9 | 2 | 2.17 |
| A16592 | XP_013346400.1; hypothetical protein AUEXF2481DRAFT_62981 [ *Aureobasidium* *subglaciale* EXF-2481] | 0 | 1.25 | 1.46 | 1.32 | 1.19 |
| A16618 | XP_013425681.1; hypothetical protein M436DRAFT_83661 [ *Aureobasidium* *namibiae* CBS 147.97] | 0 | 1.7 | 2.94 | 2.7 | 2.59 |
| A16645 | KEQ63201.1; FAD/NAD(P)-binding domain-containing protein [ *Aureobasidium* *melanogenum* CBS 110374] | 0 | 2.32 | 2.17 | 3.09 | 3.32 |
| A16647 | XP_013423854.1; S-adenosyl-L-methionine-dependent methyltransferase [ *Aureobasidium* *namibiae* CBS 147.97] | 0 | 1.09 | 0.9 | 0.91 | 0.97 |
| A16653 | KEQ63193.1; purine-cytosine permease [ *Aureobasidium* *melanogenum* CBS 110374] | 0 | 2.09 | 2.04 | 1.82 | 2.14 |
| A16658 | XP_013346264.1; hypothetical protein AUEXF2481DRAFT_27241 [ *Aureobasidium* *subglaciale* EXF-2481] | 0 | 2.04 | 2.07 | 2.3 | 1.96 |
| A16665 | OBW69153.1; Uncharacterized protein AUREO_007960 [ *Aureobasidium* *pullulans*] | 0 | 3.81 | 4.09 | 3.43 | 4.61 |
| A16666 | KEQ63032.1; hypothetical protein M437DRAFT_47970 [ *Aureobasidium* *melanogenum* CBS 110374] | 0 | 1.15 | 1.47 | 1.39 | 1.06 |
| A16675 | XP_013346384.1; hypothetical protein AUEXF2481DRAFT_46284 [ *Aureobasidium* *subglaciale* EXF-2481] | 0 | 2.88 | 3.73 | 3.53 | 3.41 |
| A16681 | KEQ83807.1; DEAD-domain-containing protein [ *Aureobasidium* *pullulans* EXF-150] | 0 | 1.07 | 1.62 | 1.51 | 1.38 |
| A16699 | XP_013424966.1; hypothetical protein M436DRAFT_74996 [ *Aureobasidium* *namibiae* CBS 147.97] | 0 | 1.84 | 1.67 | 1.42 | 1.73 |
| A16705 | KEQ83990.1; alpha/beta-hydrolase [ *Aureobasidium* *pullulans* EXF-150] | 0 | 2.94 | 3.79 | 4.11 | 3.26 |
| A16724 | OBW68509.1; 3-methylcrotonyl-CoA carboxylase subunit alpha [ *Aureobasidium* *pullulans*] | 0 | 1.81 | 2.45 | 2.74 | 2.37 |
| A16738 | KEQ88348.1; neutral amino acid permease [ *Aureobasidium* *pullulans* EXF-150] | 0 | 2.16 | 1.57 | 2.42 | 2.84 |
| A16761 | XP_013425726.1; P-loop containing nucleoside triphosphate hydrolase protein [ *Aureobasidium* *namibiae* CBS 147.97] | 0 | 1.85 | 1.73 | 1.6 | 1.31 |
| A16780 | XP_013425618.1; hypothetical protein M436DRAFT_65662 [ *Aureobasidium* *namibiae* CBS 147.97] | 0 | 2.16 | 2.78 | 2.98 | 3.22 |
| A16805 | NA | 0 | 1.27 | 0.94 | 0.78 | 1.79 |
| A16812 | KEQ83507.1; peptidase S10, serine carboxypeptidase [ *Aureobasidium* *pullulans* EXF-150] | 0 | 2.93 | 1.9 | 1.47 | 1.33 |
| A16823 | KEQ60542.1; hypothetical protein M437DRAFT_77256 [ *Aureobasidium* *melanogenum* CBS 110374] | 0 | 4.75 | 4.93 | 4.2 | 3.5 |
| A16828 | XP_013346253.1; hypothetical protein AUEXF2481DRAFT_27191 [ *Aureobasidium* *subglaciale* EXF-2481] | 0 | 0.94 | 1.65 | 1.59 | 1.73 |
| A16846 | XP_013424929.1; putative proline oxidase Put1 [ *Aureobasidium* *namibiae* CBS 147.97] | 0 | 4.24 | 2.79 | 2.39 | 2.33 |
| A16852 | XP_013428672.1; hypothetical protein M436DRAFT_62963 [ *Aureobasidium* *namibiae* CBS 147.97] | 0 | 1.81 | 2.12 | 1.84 | 1.26 |
| A16876 | KEQ84185.1; putative glutaminase [ *Aureobasidium* *pullulans* EXF-150] | 0 | 0.91 | 0.92 | 1.25 | 1.35 |
| A16887 | KEQ84067.1; S-adenosyl-L-methionine-dependent methyltransferase [ *Aureobasidium* *pullulans*] | 0 | 2.8 | 4.02 | 3.26 | 2.66 |
| A16912 | KEQ60550.1; hypothetical protein M437DRAFT_86638 [ *Aureobasidium* *melanogenum* CBS 110374] | 0 | 2.04 | 2.73 | 2.1 | 2.03 |
| A16915 | NA | 0 | 2.24 | 2.42 | 2 | 1.82 |
| A16917 | KEQ63398.1; F-box domain protein [ *Aureobasidium* *melanogenum* CBS 110374] | 0 | 1.11 | 1.3 | 1.49 | 1.13 |
| A16922 | XP_013424954.1; hypothetical protein M436DRAFT_74963 [ *Aureobasidium* *namibiae* CBS 147.97] | 0 | 1.3 | 1.46 | 1.5 | 1.05 |
| A16928 | XP_013424957.1; hypothetical protein M436DRAFT_74968 [ *Aureobasidium* *namibiae* CBS 147.97] | 0 | 1.21 | 2.18 | 2.09 | 2 |
| A16934 | XP_013341650.1; glycoside hydrolase family 28 protein [ *Aureobasidium* *subglaciale* EXF-2481] | 0 | 2 | 1.77 | 1.98 | 2.75 |
| A16958 | XP_013424803.1; hypothetical protein M436DRAFT_52738 [ *Aureobasidium* *namibiae* CBS 147.97] | 0 | 2.01 | 1.95 | 2.29 | 1.45 |
| A16988 | XP_013346507.1; hypothetical protein AUEXF2481DRAFT_105836 [ *Aureobasidium* *subglaciale* EXF-2481] | 0 | 2.84 | 2.84 | 3.79 | 4.39 |
| A16993 | OBW64165.1; putative iron-sulfur cluster assembly associated protein Nar1 [ *Aureobasidium* *pullulans*] | 0 | 2.26 | 3.57 | 3.51 | 3.32 |
| A17000 | XP_013424924.1; hypothetical protein M436DRAFT_66108 [ *Aureobasidium* *namibiae* CBS 147.97] | 0 | 1.33 | 1.48 | 1.72 | 1.78 |
| A17035 | KEQ84093.1; hypothetical protein M438DRAFT_274259 [ *Aureobasidium* *pullulans*] | 0 | 3.82 | 4.82 | 4.67 | 3.89 |
| A17043 | XP_013424810.1; hypothetical protein M436DRAFT_52788 [ *Aureobasidium* *namibiae* CBS 147.97] | 0 | 3.76 | 2.5 | 3.7 | 3.06 |
| A17047 | XP_013346427.1; hypothetical protein AUEXF2481DRAFT_63319 [ *Aureobasidium* *subglaciale* EXF-2481] | 0 | 1.46 | 1.03 | 1.67 | 1.75 |
| A17056 | KEQ60566.1; hypothetical protein M437DRAFT_86653 [ *Aureobasidium* *melanogenum* CBS 110374] | 0 | 1.88 | 3.07 | 2.97 | 3.21 |
| A17090 | XP_013341588.1; glycoside hydrolase family 16 protein [ *Aureobasidium* *subglaciale* EXF-2481] | 0 | 2.29 | 1.94 | 2.51 | 2.32 |
| A17119 | XP_013423885.1; putative multicopper oxidase, type 1 [ *Aureobasidium* *namibiae* CBS 147.97] | 0 | 2.8 | 2.95 | 3.44 | 4.04 |
| A17126 | KEQ64004.1; putative ABC transporter [ *Aureobasidium* *melanogenum* CBS 110374] | 0 | 2.58 | 2.37 | 2.5 | 3.39 |
| A17132 | KEQ57549.1; putative serine palmitoyltransferase 2 [ *Aureobasidium* *melanogenum* CBS 110374] | 0 | 0.87 | 0.88 | 1.02 | 1.09 |
| A17191 | KEQ64723.1; UAA transporter [ *Aureobasidium* *melanogenum* CBS 110374] | 0 | 1.87 | 2.66 | 3.05 | 2.43 |
| A17201 | KEQ64734.1; hypothetical protein M437DRAFT_82730 [ *Aureobasidium* *melanogenum* CBS 110374] | 0 | 1.02 | 1.51 | 1.65 | 1.58 |
| A17229 | KEQ64765.1; hypothetical protein M437DRAFT_73689 [ *Aureobasidium* *melanogenum* CBS 110374] | 0 | 1.36 | 1.12 | 1.63 | 1.55 |
| A17238 | KEQ64773.1; alpha/beta-hydrolase [ *Aureobasidium* *melanogenum* CBS 110374] | 0 | 1.65 | 1.14 | 1.05 | 0.94 |
| A17241 | KEQ64776.1; D-galacturonic acid reductase-like protein [ *Aureobasidium* *melanogenum* CBS 110374] | 0 | 2.27 | 1.64 | 2.5 | 2.02 |
| A17242 | KEQ64777.1; aldolase [ *Aureobasidium* *melanogenum* CBS 110374] | 0 | 2.96 | 2.68 | 3.55 | 3.58 |
| A17264 | KEQ60952.1; sphingosine-1-phosphate phosphohydrolase-like protein [ *Aureobasidium* *melanogenum* CBS 110374] | 0 | 0.84 | 0.72 | 1.05 | 0.87 |
| A17311 | KEQ84595.1; mitochondrial carrier [ *Aureobasidium* *pullulans* EXF-150] | 0 | 3.05 | 2.32 | 2.4 | 3.44 |
| A17314 | KEQ60899.1; MFS general substrate transporter [ *Aureobasidium* *melanogenum* CBS 110374] | 0 | 1.97 | 2.09 | 2.58 | 2.25 |
| A17336 | KEQ64807.1; FAD dependent oxidoreductase [ *Aureobasidium* *melanogenum* CBS 110374] | 0 | 3.27 | 3.71 | 3.4 | 3.52 |
| A17341 | KEQ64801.1; glycerol kinase [ *Aureobasidium* *melanogenum* CBS 110374] | 0 | 2.11 | 2.94 | 3.68 | 2.93 |
| A17362 | KEQ60977.1; glycoside hydrolase [ *Aureobasidium* *melanogenum* CBS 110374] | 0 | 2.7 | 3.97 | 3.58 | 3.13 |
| A17387 | KEQ64619.1; glycoside hydrolase [ *Aureobasidium* *melanogenum* CBS 110374] | 0 | 2.14 | 2.17 | 1.73 | 1.56 |
| A17415 | KEQ64587.1; alpha-1,2-mannosidase, putative subfamily [ *Aureobasidium* *melanogenum* CBS 110374] | 0 | 2.55 | 1.94 | 2.25 | 1.44 |
| A17477 | KEQ61001.1; phosphoglycerate mutase-like protein [ *Aureobasidium* *melanogenum* CBS 110374] | 0 | 1.12 | 1.31 | 1.16 | 0.98 |
| A17508 | KEQ64647.1; alpha-glucosidase [ *Aureobasidium* *melanogenum* CBS 110374] | 0 | 1.39 | 1.62 | 2.05 | 1.69 |
| A17515 | KEQ78513.1; hypothetical protein M438DRAFT_340591 [ *Aureobasidium* *pullulans* EXF-150] | 0 | 1.58 | 1.88 | 1.83 | 1.3 |
| A17523 | KEQ64666.1; succinyl-CoA synthetase-like protein [ *Aureobasidium* *melanogenum* CBS 110374] | 0 | 0.89 | 1.21 | 1.33 | 1.16 |
| A17546 | XP_013343010.1; hypothetical protein AUEXF2481DRAFT_271648 [ *Aureobasidium* *subglaciale* EXF-2481] | 0 | 3.3 | 4.42 | 4.13 | 3.07 |
| A17598 | KEQ64593.1; hypothetical protein M437DRAFT_43740 [ *Aureobasidium* *melanogenum* CBS 110374] | 0 | 4.16 | 2.58 | 3.38 | 3.48 |
| A17604 | OBW68374.1; Glycoside hydrolase [ *Aureobasidium* *pullulans*] | 0 | 3.27 | 2.76 | 2.67 | 3.88 |
| A17620 | KEQ64486.1; hypothetical protein M437DRAFT_64142 [ *Aureobasidium* *melanogenum* CBS 110374] | 0 | 2.55 | 2.55 | 2.76 | 2.36 |
| A17669 | KEQ63721.1; hypothetical protein M437DRAFT_26163, partial [ *Aureobasidium* *melanogenum* CBS 110374] | 0 | 1.69 | 1.6 | 0.81 | 1.1 |
| A17682 | KEQ63743.1; acid protease, partial [ *Aureobasidium* *melanogenum* CBS 110374] | 0 | 1.96 | 2.92 | 2.51 | 2.3 |
| A17686 | KEQ63746.1; hypothetical protein M437DRAFT_46090, partial [ *Aureobasidium* *melanogenum* CBS 110374] | 0 | 1.81 | 1.57 | 2.15 | 2.4 |
| A17743 | KEQ63814.1; hypothetical protein M437DRAFT_83526 [ *Aureobasidium* *melanogenum* CBS 110374] | 0 | 1.39 | 1.15 | 1.43 | 1.5 |
| A17752 | KEQ63821.1; WD40 repeat-like protein [ *Aureobasidium* *melanogenum* CBS 110374] | 0 | 0.93 | 1.54 | 1.55 | 1.68 |
| A17753 | KEQ63820.1; hypothetical protein M437DRAFT_65135 [ *Aureobasidium* *melanogenum* CBS 110374] | 0 | 1.4 | 1.64 | 1.98 | 1.97 |
| A17786 | XP_013344643.1; hypothetical protein AUEXF2481DRAFT_64715 [ *Aureobasidium* *subglaciale* EXF-2481] | 0 | 0.89 | 0.78 | 1.01 | 0.66 |
| A17788 | PPJ57381.1; hypothetical protein CBER1_01430 [Cercospora berteroae] | 0 | 1.14 | 1.74 | 1.88 | 2.04 |
| A17798 | XP_013344505.1; hypothetical protein AUEXF2481DRAFT_28829 [ *Aureobasidium* *subglaciale* EXF-2481] | 0 | 1.39 | 1.33 | 0.99 | 0.74 |
| A17832 | XP_007925583.1; hypothetical protein MYCFIDRAFT_133529 [Pseudocercospora fijiensis CIRAD86] | 0 | 1.08 | 1.44 | 1.34 | 1.81 |
| A17838 | KEQ63919.1; hypothetical protein M437DRAFT_65218 [ *Aureobasidium* *melanogenum* CBS 110374] | 0 | 5.01 | 6.75 | 5.96 | 4.92 |
| A17840 | KEQ63923.1; rhomboid-domain-containing protein [ *Aureobasidium* *melanogenum* CBS 110374] | 0 | 1.28 | 1.4 | 1.33 | 1.12 |
| A17880 | KEQ63963.1; cytochrome c peroxidase-like protein [ *Aureobasidium* *melanogenum* CBS 110374] | 0 | 1.73 | 1.9 | 2.12 | 1.31 |
| A17890 | PNS16672.1; hypothetical protein CAC42_4636 [Sphaceloma murrayae] | 0 | 1.12 | 1.21 | 0.95 | 0.87 |
| A17903 | XP_013427989.1; hypothetical protein M436DRAFT_45223 [ *Aureobasidium* *namibiae* CBS 147.97] | 0 | 1.25 | 0.88 | 0.74 | 0.67 |
| A17905 | OBW68550.1; Uncharacterized protein AUREO_014010 [ *Aureobasidium* *pullulans*] | 0 | 0.87 | 1.11 | 0.79 | 1.08 |
| A00116 | KEQ65550.1; hypothetical protein M437DRAFT_72450 [ *Aureobasidium* *melanogenum* CBS 110374] | 0 | 4.4 | 3.81 | 3.44 | 3.58 |
| A00133 | XP_013344863.1; hypothetical protein AUEXF2481DRAFT_4163 [ *Aureobasidium* *subglaciale* EXF-2481] | 0 | 1.67 | 1.52 | 1.59 | 2.13 |
| A00148 | KEQ65581.1; glycoside hydrolase family 61 protein [ *Aureobasidium* *melanogenum* CBS 110374] | 0 | 1.58 | 2.05 | 2.38 | 2.58 |
| A00156 | KEQ65591.1; putative MFS sugar transporter [ *Aureobasidium* *melanogenum* CBS 110374] | 0 | 1.86 | 2.63 | 2.42 | 2.84 |
| A00170 | KEQ85395.1; hypothetical protein M438DRAFT_364495 [ *Aureobasidium* *pullulans* EXF-150] | 0 | 1.37 | 0.93 | 1.41 | 0.92 |
| A00182 | KEQ85381.1; putative sugar transporter [ *Aureobasidium* *pullulans* EXF-150] | 0 | 1.7 | 2.19 | 2.16 | 2.09 |
| A00207 | KEQ65794.1; putative alpha-L-fucosidase [ *Aureobasidium* *melanogenum* CBS 110374] | 0 | 1.34 | 1.57 | 1.78 | 1.68 |
| A00225 | XP_013344954.1; hypothetical protein AUEXF2481DRAFT_38688 [ *Aureobasidium* *subglaciale* EXF-2481] | 0 | 3.23 | 4.48 | 4.3 | 3.53 |
| A00239 | KEQ65836.1; amidase signature enzyme [ *Aureobasidium* *melanogenum* CBS 110374] | 0 | 2.88 | 3.28 | 2.88 | 2.53 |
| A00249 | KEQ65823.1; DUF1793-domain-containing protein [ *Aureobasidium* *melanogenum* CBS 110374] | 0 | 1.99 | 1.88 | 2.12 | 1.38 |
| A00257 | KEQ59516.1; hypothetical protein M437DRAFT_56530 [ *Aureobasidium* *melanogenum* CBS 110374] | 0 | 0.86 | 1.36 | 1.08 | 1.18 |
| A00260 | KEQ65636.1; MFS general substrate transporter [ *Aureobasidium* *melanogenum* CBS 110374] | 0 | 2.97 | 1.83 | 2.14 | 1.84 |
| A00261 | XP_013344813.1; hypothetical protein AUEXF2481DRAFT_3955 [ *Aureobasidium* *subglaciale* EXF-2481] | 0 | 2.06 | 2.56 | 2.93 | 2.69 |
| A00267 | KEQ65629.1; hypothetical protein M437DRAFT_72524 [ *Aureobasidium* *melanogenum* CBS 110374] | 0 | 3.35 | 2.42 | 3.14 | 2.97 |
| A00296 | XP_013345982.1; glycoside hydrolase family 29 protein [ *Aureobasidium* *subglaciale* EXF-2481] | 0 | 1.3 | 1.3 | 1.25 | 1 |
| A00301 | KEQ59521.1; hypothetical protein M437DRAFT_56656 [ *Aureobasidium* *melanogenum* CBS 110374] | 0 | 0.98 | 1.21 | 1.2 | 0.93 |
| A00310 | KEQ65741.1; PGX, glycoside hydrolase family 28 protein [ *Aureobasidium* *melanogenum* CBS 110374] | 0 | 3.14 | 4.78 | 4.14 | 4.29 |
| A00334 | XP_008030211.1; hypothetical protein SETTUDRAFT_165387 [Exserohilum turcica Et28A] | 0 | 1.49 | 1.31 | 1.87 | 1.57 |
| A00346 | KEQ59525.1; hypothetical protein M437DRAFT_56470 [ *Aureobasidium* *melanogenum* CBS 110374] | 0 | 2.08 | 1.8 | 2.61 | 2.45 |
| A00354 | KEQ65699.1; hypothetical protein M437DRAFT_63249 [ *Aureobasidium* *melanogenum* CBS 110374] | 0 | 3.51 | 3.98 | 3.52 | 4.73 |
| A00360 | KEQ65694.1; hypothetical protein M437DRAFT_81547 [ *Aureobasidium* *melanogenum* CBS 110374] | 0 | 1.58 | 1.38 | 1.82 | 1.04 |
| A00426 | KEQ65848.1; hypothetical protein M437DRAFT_63391 [ *Aureobasidium* *melanogenum* CBS 110374] | 0 | 1.19 | 1.1 | 0.61 | 0.64 |
| A00439 | KEQ65864.1; hypothetical protein M437DRAFT_41125 [ *Aureobasidium* *melanogenum* CBS 110374] | 0 | 1.91 | 2.36 | 2.13 | 2.28 |
| A00442 | KEQ65866.1; Metallo-dependent phosphatase [ *Aureobasidium* *melanogenum* CBS 110374] | 0 | 2.01 | 2.66 | 2.56 | 2.43 |
| A00444 | KEQ65868.1; DUF336-domain-containing protein [ *Aureobasidium* *melanogenum* CBS 110374] | 0 | 1.27 | 1.8 | 1.43 | 1.84 |
| A00454 | KEQ65876.1; aquaporin-like protein [ *Aureobasidium* *melanogenum* CBS 110374] | 0 | 0.84 | 1.02 | 0.92 | 1.19 |
| A00486 | KEQ65905.1; hypothetical protein M437DRAFT_41243 [ *Aureobasidium* *melanogenum* CBS 110374] | 0 | 2.97 | 4.14 | 4.16 | 3.55 |
| A00552 | KEQ66096.1; hypothetical protein M437DRAFT_63610 [ *Aureobasidium* *melanogenum* CBS 110374] | 0 | 1.22 | 1.86 | 1.21 | 1.6 |
| A00557 | XP_013422969.1; glycoside hydrolase [ *Aureobasidium* *namibiae* CBS 147.97] | 0 | 2.49 | 3.07 | 2.38 | 2.81 |
| A00571 | OBW67782.1; Uncharacterized protein AUREO_021440 [ *Aureobasidium* *pullulans*] | 0 | 2.84 | 4.67 | 4.53 | 4 |
| A00598 | KEQ65997.1; putative RNA polymerase I and III transcription factor complex component Tbp [ *Aureobasidium* *melanogenum* CBS 110374] | 0 | 1.47 | 1.53 | 1.39 | 1.35 |
| A00613 | KEQ66020.1; hypothetical protein M437DRAFT_41354 [ *Aureobasidium* *melanogenum* CBS 110374] | 0 | 1.36 | 2.47 | 2.1 | 2.43 |
| A00649 | NA | 0 | 1.96 | 1.43 | 1.35 | 1.56 |
| A00666 | KEQ65976.1; hypothetical protein M437DRAFT_63509 [ *Aureobasidium* *melanogenum* CBS 110374] | 0 | 2.44 | 1.77 | 1.52 | 1.28 |
| A00676 | XP_013341397.1; hypothetical protein AUEXF2481DRAFT_49598, partial [ *Aureobasidium* *subglaciale* EXF-2481] | 0 | 0.8 | 1.13 | 0.99 | 0.84 |
| A00718 | KEQ79190.1; hypothetical protein M438DRAFT_284122, partial [ *Aureobasidium* *pullulans* EXF-150] | 0 | 1.77 | 2.38 | 1.68 | 2.14 |
| A00750 | KEQ59626.1; hypothetical protein M437DRAFT_56313 [ *Aureobasidium* *melanogenum* CBS 110374] | 0 | 2.79 | 2.35 | 3.04 | 2.81 |
| A00766 | XP_013422897.1; hypothetical protein M436DRAFT_57680 [ *Aureobasidium* *namibiae* CBS 147.97] | 0 | 2.19 | 1.7 | 1.74 | 2.18 |
| A00814 | XP_013347854.1; hypothetical protein AUEXF2481DRAFT_1014 [ *Aureobasidium* *subglaciale* EXF-2481] | 0 | 2.59 | 1.48 | 1.91 | 2.2 |
| A00822 | KEQ67933.1; hypothetical protein M437DRAFT_62297 [ *Aureobasidium* *melanogenum* CBS 110374] | 0 | 3.35 | 3.07 | 3.59 | 4.01 |
| A00873 | NA | 0 | 1.12 | 1.45 | 1.45 | 1.5 |
| A00891 | KEQ67761.1; galactose mutarotase-like protein [ *Aureobasidium* *melanogenum* CBS 110374] | 0 | 4.05 | 3.79 | 3.65 | 5.14 |
| A00896 | XP_013348327.1; hypothetical protein AUEXF2481DRAFT_85269 [ *Aureobasidium* *subglaciale* EXF-2481] | 0 | 1.93 | 2.21 | 1.71 | 1.88 |
| A00914 | KEQ68035.1; Thioesterase/thiol ester dehydrase-isomerase [ *Aureobasidium* *melanogenum* CBS 110374] | 0 | 1.39 | 1.52 | 1.13 | 1.08 |
| A00920 | OBW67354.1; RNA-binding domain-containing protein [ *Aureobasidium* *pullulans*] | 0 | 1.09 | 1.11 | 0.92 | 0.86 |
| A00921 | XP_013348276.1; glycoside hydrolase family 3 protein [ *Aureobasidium* *subglaciale* EXF-2481] | 0 | 2.36 | 2.76 | 2.99 | 3.78 |
| A00922 | XP_013348270.1; glycoside hydrolase family 3 protein [ *Aureobasidium* *subglaciale* EXF-2481] | 0 | 2.42 | 2.72 | 3.19 | 3.07 |
| A00929 | KEQ89815.1; peptidase C1B, bleomycin hydrolase [ *Aureobasidium* *pullulans* EXF-150] | 0 | 4.69 | 3.26 | 4.18 | 4.48 |
| A00935 | NA | 0 | 1.12 | 1.39 | 1.04 | 1.17 |
| A00948 | KEQ90057.1; hypothetical protein M438DRAFT_262211 [ *Aureobasidium* *pullulans* EXF-150] | 0 | 1.4 | 1.18 | 1.2 | 1.51 |
| A00951 | XP_013348393.1; hypothetical protein AUEXF2481DRAFT_179093 [ *Aureobasidium* *subglaciale* EXF-2481] | 0 | 1.72 | 1.34 | 1.36 | 1.3 |
| A00962 | XP_013348124.1; hypothetical protein AUEXF2481DRAFT_35377 [ *Aureobasidium* *subglaciale* EXF-2481] | 0 | 2.65 | 2.96 | 2.92 | 2.44 |
| A00981 | XP_013348378.1; hypothetical protein AUEXF2481DRAFT_176902 [ *Aureobasidium* *subglaciale* EXF-2481] | 0 | 0.93 | 1.17 | 0.88 | 0.74 |
| A01026 | XP_013347956.1; hypothetical protein AUEXF2481DRAFT_1411 [ *Aureobasidium* *subglaciale* EXF-2481] | 0 | 1.88 | 1.72 | 1.4 | 1.82 |
| A01056 | XP_013347982.1; hypothetical protein AUEXF2481DRAFT_25293 [ *Aureobasidium* *subglaciale* EXF-2481] | 0 | 0.97 | 1.11 | 1.29 | 1.22 |
| A01068 | KEQ61718.1; hypothetical protein M437DRAFT_85668 [ *Aureobasidium* *melanogenum* CBS 110374] | 0 | 3.5 | 4.24 | 4.46 | 4.26 |
| A01081 | XP_013348065.1; hypothetical protein AUEXF2481DRAFT_25797 [ *Aureobasidium* *subglaciale* EXF-2481] | 0 | 1.33 | 1.18 | 1.28 | 1.8 |
| A01099 | XP_013348489.1; hypothetical protein AUEXF2481DRAFT_696680 [ *Aureobasidium* *subglaciale* EXF-2481] | 0 | 1.92 | 1.81 | 1.44 | 1.29 |
| A01122 | XP_013348481.1; hypothetical protein AUEXF2481DRAFT_200763 [ *Aureobasidium* *subglaciale* EXF-2481] | 0 | 4.07 | 3.83 | 4.13 | 3.55 |
| A01148 | RBQ74183.1; hypothetical protein FVER14953_08370 [Fusarium verticillioides] | 0 | 2.14 | 1.92 | 2.61 | 2.9 |
| A01152 | KEQ85161.1; hypothetical protein M438DRAFT_365125 [ *Aureobasidium* *pullulans* EXF-150] | 0 | 0.84 | 0.75 | 0.76 | 1 |
| A01177 | KEQ82015.1; hypothetical protein M438DRAFT_347707 [ *Aureobasidium* *pullulans* EXF-150] | 0 | 2.35 | 2.39 | 2.9 | 2.67 |
| A01186 | KEQ59390.1; ATP-dependent RNA helicase CHL1 [ *Aureobasidium* *melanogenum* CBS 110374] | 0 | 1.02 | 1.22 | 1.43 | 1.32 |
| A01192 | OBW65734.1; Inositol monophosphatase [ *Aureobasidium* *pullulans*] | 0 | 2.69 | 2.36 | 2.36 | 1.67 |
| A01236 | KEQ79162.1; hypothetical protein M438DRAFT_340076 [ *Aureobasidium* *pullulans* EXF-150] | 0 | 4.75 | 5.47 | 5.99 | 5.07 |
| A01237 | KFZ03422.1; hypothetical protein V502_10960 [Pseudogymnoascus sp. VKM F-4520 FW-2644] | 0 | 3.1 | 1.93 | 2.89 | 3.56 |
| A01256 | XP_013339201.1; hypothetical protein AUEXF2481DRAFT_9179 [ *Aureobasidium* *subglaciale* EXF-2481] | 0 | 1.25 | 1.29 | 1.56 | 1.02 |
| A01267 | KEQ61523.1; hypothetical protein M437DRAFT_51340 [ *Aureobasidium* *melanogenum* CBS 110374] | 0 | 2 | 3.23 | 3.57 | 3.43 |
| A01284 | XP_013339236.1; hypothetical protein AUEXF2481DRAFT_48575 [ *Aureobasidium* *subglaciale* EXF-2481] | 0 | 1.72 | 2 | 2.06 | 1.39 |
| A01287 | KEQ83113.1; nucleotide sugar dehydrogenase [ *Aureobasidium* *pullulans* EXF-150] | 0 | 0.95 | 0.72 | 0.91 | 1.03 |
| A01288 | KEQ61553.1; TPT-domain-containing protein [ *Aureobasidium* *melanogenum* CBS 110374] | 0 | 0.95 | 1.31 | 1.13 | 1.66 |
| A01292 | KEQ83030.1; hypothetical protein M438DRAFT_346683 [ *Aureobasidium* *pullulans* EXF-150] | 0 | 1.59 | 1.09 | 0.94 | 1.14 |
| A01297 | OBW68041.1; PTPLA-domain-containing protein [ *Aureobasidium* *pullulans*] | 0 | 1.94 | 2.86 | 2.82 | 3.12 |
| A01321 | XP_013339830.1; hypothetical protein AUEXF2481DRAFT_33110 [ *Aureobasidium* *subglaciale* EXF-2481] | 0 | 1.63 | 1.44 | 1.93 | 1.09 |
| A01322 | KEQ83066.1; amino acid permease [ *Aureobasidium* *pullulans* EXF-150] | 0 | 1.69 | 1.87 | 1.89 | 1.34 |
| A01332 | XP_013426141.1; hypothetical protein M436DRAFT_49176 [ *Aureobasidium* *namibiae* CBS 147.97] | 0 | 1.01 | 1.65 | 1.46 | 1.36 |
| A01341 | KEQ83102.1; carboxylesterase family protein [ *Aureobasidium* *pullulans* EXF-150] | 0 | 1.13 | 1.05 | 1.56 | 1.36 |
| A01350 | KEQ58097.1; Arginase/deacetylase [ *Aureobasidium* *melanogenum* CBS 110374] | 0 | 1.43 | 1 | 1.21 | 1.1 |
| A01360 | KEQ83145.1; hypothetical protein M438DRAFT_366519 [ *Aureobasidium* *pullulans* EXF-150] | 0 | 1.7 | 1.67 | 1.48 | 1.16 |
| A01369 | OBW69239.1; General substrate transporter [ *Aureobasidium* *pullulans*] | 0 | 1.93 | 1.97 | 1.77 | 1.79 |
| A01371 | OBW69237.1; Uncharacterized protein AUREO_006730 [ *Aureobasidium* *pullulans*] | 0 | 1.12 | 0.78 | 1.16 | 1.47 |
| A01374 | XP_013347813.1; hypothetical protein AUEXF2481DRAFT_840 [ *Aureobasidium* *subglaciale* EXF-2481] | 0 | 0.95 | 0.95 | 1.37 | 1.19 |
| A01405 | KEQ58528.1; hypothetical protein M437DRAFT_59087 [ *Aureobasidium* *melanogenum* CBS 110374] | 0 | 1.92 | 1.65 | 1.87 | 2.53 |
| A01443 | KEQ89774.1; rRNA adenine dimethylase [ *Aureobasidium* *pullulans* EXF-150] | 0 | 3.09 | 4.32 | 4.28 | 3.6 |
| A01447 | XP_013348277.1; hypothetical protein AUEXF2481DRAFT_61752 [ *Aureobasidium* *subglaciale* EXF-2481] | 0 | 1.38 | 1.5 | 1.51 | 1.53 |
| A01486 | XP_013341783.1; hypothetical protein AUEXF2481DRAFT_31436 [ *Aureobasidium* *subglaciale* EXF-2481] | 0 | 2.1 | 3.27 | 3.52 | 2.95 |
| A01487 | KEQ58453.1; GroES-like protein [ *Aureobasidium* *melanogenum* CBS 110374] | 0 | 3.73 | 4.66 | 4.82 | 3.88 |
| A01502 | KEQ85066.1; hypothetical protein M438DRAFT_405375 [ *Aureobasidium* *pullulans* EXF-150] | 0 | 3.64 | 3.12 | 2.7 | 3.35 |
| A01508 | XP_013430074.1; Metallo-hydrolase/oxidoreductase [ *Aureobasidium* *namibiae* CBS 147.97] | 0 | 2.91 | 2.85 | 3.34 | 3.47 |
| A01509 | KEQ58425.1; hypothetical protein M437DRAFT_88574 [ *Aureobasidium* *melanogenum* CBS 110374] | 0 | 1.02 | 1.13 | 1.01 | 1.06 |
| A01531 | XP_013341916.1; hypothetical protein AUEXF2481DRAFT_338624 [ *Aureobasidium* *subglaciale* EXF-2481] | 0 | 3.75 | 3.86 | 4.18 | 2.91 |
| A01553 | KEQ58304.1; Di-copper centre-containing protein [ *Aureobasidium* *melanogenum* CBS 110374] | 0 | 3.7 | 4.4 | 4 | 4.73 |
| A01610 | KEQ58398.1; adenosine deaminase [ *Aureobasidium* *melanogenum* CBS 110374] | 0 | 3.19 | 3.83 | 4.01 | 3.01 |
| A01645 | XP_013430097.1; MFS general substrate transporter [ *Aureobasidium* *namibiae* CBS 147.97] | 0 | 3.34 | 4.01 | 4.21 | 3.16 |
| A01646 | XP_013341843.1; hypothetical protein AUEXF2481DRAFT_47634 [ *Aureobasidium* *subglaciale* EXF-2481] | 0 | 3 | 3.83 | 3.3 | 2.98 |
| A01654 | XP_013430197.1; ABC transporter-like protein [ *Aureobasidium* *namibiae* CBS 147.97] | 0 | 2.56 | 2.83 | 2.3 | 2.01 |
| A01668 | XP_013430006.1; MFS general substrate transporter [ *Aureobasidium* *namibiae* CBS 147.97] | 0 | 0.64 | 1.11 | 0.87 | 0.98 |
| A01695 | XP_013339278.1; hypothetical protein AUEXF2481DRAFT_9078 [ *Aureobasidium* *subglaciale* EXF-2481] | 0 | 3.42 | 2.48 | 2.1 | 3.22 |
| A01705 | OBW64938.1; Uncharacterized protein AUREO_049980 [ *Aureobasidium* *pullulans*] | 0 | 2.18 | 3.17 | 3.06 | 2.5 |
| A01742 | KEQ64936.1; Dehydroquinate synthase-like protein [ *Aureobasidium* *melanogenum* CBS 110374] | 0 | 2.31 | 2.27 | 2.54 | 1.51 |
| A01745 | XP_013430501.1; kinase-like protein [ *Aureobasidium* *namibiae* CBS 147.97] | 0 | 1.69 | 1.93 | 1.85 | 1.17 |
| A01749 | XP_013340875.1; glycoside hydrolase family 30 protein [ *Aureobasidium* *subglaciale* EXF-2481] | 0 | 2.24 | 1.65 | 2.47 | 3.03 |
| A01752 | XP_013430483.1; hypothetical protein M436DRAFT_79259 [ *Aureobasidium* *namibiae* CBS 147.97] | 0 | 1.38 | 2.11 | 2.03 | 1.8 |
| A01762 | KEQ81334.1; hypothetical protein M438DRAFT_408088 [ *Aureobasidium* *pullulans* EXF-150] | 0 | 2.09 | 1.94 | 2.46 | 1.99 |
| A01785 | KEQ58269.1; hypothetical protein M437DRAFT_88735 [ *Aureobasidium* *melanogenum* CBS 110374] | 0 | 2.57 | 1.71 | 1.32 | 2.16 |
| A01791 | XP_013341751.1; hypothetical protein AUEXF2481DRAFT_31269 [ *Aureobasidium* *subglaciale* EXF-2481] | 0 | 1.53 | 1.26 | 1.11 | 0.92 |
| A01792 | KEQ84858.1; hypothetical protein M438DRAFT_334709 [ *Aureobasidium* *pullulans* EXF-150] | 0 | 0.89 | 1.27 | 1.07 | 0.97 |
| A01796 | KEQ84861.1; hypothetical protein M438DRAFT_345090 [ *Aureobasidium* *pullulans* EXF-150] | 0 | 1.93 | 2.32 | 1.92 | 1.68 |
| A01821 | KEQ80781.1; AMP deaminase [ *Aureobasidium* *pullulans* EXF-150] | 0 | 0.92 | 1.06 | 1.26 | 0.97 |
| A01831 | OBW66636.1; Polysaccharide lyase family 4 protein [ *Aureobasidium* *pullulans*] | 0 | 3.52 | 2.76 | 4.12 | 4.31 |
| A01836 | XP_013429976.1; hypothetical protein M436DRAFT_39253 [ *Aureobasidium* *namibiae* CBS 147.97] | 0 | 0.91 | 1 | 0.6 | 1.24 |
| A01846 | XP_013430533.1; hypothetical protein M436DRAFT_79512 [ *Aureobasidium* *namibiae* CBS 147.97] | 0 | 1.75 | 2.36 | 1.74 | 3.2 |
| A01858 | KEQ61816.1; hypothetical protein M437DRAFT_50549 [ *Aureobasidium* *melanogenum* CBS 110374] | 0 | 3.03 | 2.16 | 2.81 | 3.14 |
| A01874 | KEQ61802.1; hypothetical protein M437DRAFT_50677 [ *Aureobasidium* *melanogenum* CBS 110374] | 0 | 0.81 | 0.83 | 0.86 | 1.01 |
| A01922 | XP_013343312.1; hypothetical protein AUEXF2481DRAFT_252097 [ *Aureobasidium* *subglaciale* EXF-2481] | 0 | 1.59 | 1.59 | 1.3 | 1.76 |
| A01940 | XP_013340053.1; carbohydrate-binding module family 1 protein [ *Aureobasidium* *subglaciale* EXF-2481] | 0 | 1.24 | 0.9 | 0.94 | 1 |
| A01963 | XP_013343316.1; hypothetical protein AUEXF2481DRAFT_252778 [ *Aureobasidium* *subglaciale* EXF-2481] | 0 | 3.17 | 2.91 | 3.14 | 3.07 |
| A01975 | KGO73238.1; ABC transporter, integral membrane type 1 [Penicillium italicum] | 0 | 1.77 | 2.09 | 1.72 | 1.42 |
| A02039 | KEQ61941.1; putative mitochondrial ornithine carrier protein AmcA/Ort1 [ *Aureobasidium* *melanogenum* CBS 110374] | 0 | 1.69 | 1.73 | 1.81 | 1.51 |
| A02084 | KEQ81294.1; hypothetical protein M438DRAFT_358014 [ *Aureobasidium* *pullulans* EXF-150] | 0 | 2.99 | 1.82 | 2.43 | 3.09 |
| A02104 | KEQ58608.1; hypothetical protein M437DRAFT_69765 [ *Aureobasidium* *melanogenum* CBS 110374] | 0 | 2.06 | 1.87 | 2.3 | 2.62 |
| A02106 | OBW65238.1; L domain-like protein [ *Aureobasidium* *pullulans*] | 0 | 3.18 | 3.38 | 2.69 | 2.37 |
| A02115 | OBW66249.1; NAD(P)-binding protein [ *Aureobasidium* *pullulans*] | 0 | 3.51 | 3.65 | 3.92 | 4.98 |
| A02117 | KEQ85182.1; hypothetical protein M438DRAFT_405468 [ *Aureobasidium* *pullulans* EXF-150] | 0 | 1.72 | 2.14 | 1.91 | 2.49 |
| A02121 | KEQ85177.1; membrane protein [ *Aureobasidium* *pullulans* EXF-150] | 0 | 1.16 | 0.48 | 0.75 | 0.63 |
| A02179 | KEQ64752.1; hypothetical protein M437DRAFT_82749 [ *Aureobasidium* *melanogenum* CBS 110374] | 0 | 1.71 | 2.79 | 2.73 | 2.46 |
| A02187 | XP_013428256.1; D-galacturonic acid reductase-like protein [ *Aureobasidium* *namibiae* CBS 147.97] | 0 | 1.7 | 1.47 | 2.12 | 1.74 |
| A02188 | KEQ80752.1; aldolase [ *Aureobasidium* *pullulans* EXF-150] | 0 | 1.34 | 0.96 | 1.57 | 1.17 |
| A02202 | XP_013428065.1; hypothetical protein M436DRAFT_45611 [ *Aureobasidium* *namibiae* CBS 147.97] | 0 | 0.95 | 0.73 | 1.13 | 1.19 |
| A02210 | OBW68717.1; Maleylacetoacetate isomerase [ *Aureobasidium* *pullulans*] | 0 | 2.02 | 1.35 | 2.17 | 2.69 |
| A02211 | XP_013428194.1; hypothetical protein M436DRAFT_72202 [ *Aureobasidium* *namibiae* CBS 147.97] | 0 | 1.93 | 1.79 | 2.21 | 2.34 |
| A02213 | KEQ60899.1; MFS general substrate transporter [ *Aureobasidium* *melanogenum* CBS 110374] | 0 | 1.42 | 1.53 | 1.74 | 2 |
| A02220 | NA | 0 | 2.36 | 3.46 | 4.03 | 3.83 |
| A02232 | KEQ64810.1; hypothetical protein M437DRAFT_82803 [ *Aureobasidium* *melanogenum* CBS 110374] | 0 | 1.87 | 1.02 | 1.12 | 1.66 |
| A02235 | KEQ84297.1; FAD dependent oxidoreductase [ *Aureobasidium* *pullulans* EXF-150] | 0 | 3.06 | 2.93 | 3.36 | 3.45 |
| A02272 | NA | 0 | 2.69 | 2.83 | 1.83 | 2.45 |
| A02278 | XP_013428285.1; hypothetical protein M436DRAFT_81298 [ *Aureobasidium* *namibiae* CBS 147.97] | 0 | 3.08 | 4.4 | 4.89 | 4.5 |
| A02303 | XP_013342778.1; hypothetical protein AUEXF2481DRAFT_5907 [ *Aureobasidium* *subglaciale* EXF-2481] | 0 | 1 | 1.11 | 0.95 | 0.73 |
| A02328 | KEQ61001.1; phosphoglycerate mutase-like protein [ *Aureobasidium* *melanogenum* CBS 110374] | 0 | 1.56 | 1.61 | 1.89 | 2.11 |
| A02336 | XP_013342800.1; hypothetical protein AUEXF2481DRAFT_6001 [ *Aureobasidium* *subglaciale* EXF-2481] | 0 | 1.42 | 1.94 | 1.75 | 1.48 |
| A02337 | KEQ59515.1; hypothetical protein M437DRAFT_68852 [ *Aureobasidium* *melanogenum* CBS 110374] | 0 | 2.03 | 0.93 | 1.79 | 1.21 |
| A02346 | XP_013343010.1; hypothetical protein AUEXF2481DRAFT_271648 [ *Aureobasidium* *subglaciale* EXF-2481] | 0 | 1.31 | 2.04 | 2.44 | 2.14 |
| A02361 | XP_013428071.1; hypothetical protein M436DRAFT_45648, partial [ *Aureobasidium* *namibiae* CBS 147.97] | 0 | 1.17 | 0.76 | 0.94 | 0.57 |
| A02401 | XP_013342926.1; glycoside hydrolase family 12 protein [ *Aureobasidium* *subglaciale* EXF-2481] | 0 | 1.46 | 1.71 | 2.09 | 2.25 |
| A02414 | KEQ78508.1; short-chain specific acyl-CoA dehydrogenase mitochondrial precursor [ *Aureobasidium* *pullulans* EXF-150] | 0 | 2.02 | 2.94 | 3.07 | 3.21 |
| A02438 | KEQ64517.1; putative zinc-binding alcohol dehydrogenase [ *Aureobasidium* *melanogenum* CBS 110374] | 0 | 1.49 | 1.79 | 1.62 | 1.18 |
| A02445 | XP_013340416.1; hypothetical protein AUEXF2481DRAFT_69296 [ *Aureobasidium* *subglaciale* EXF-2481] | 0 | 1.07 | 1.28 | 0.96 | 0.97 |
| A02456 | PNS16356.1; Ribonuclease H1 [Sphaceloma murrayae] | 0 | 7.53 | 5.48 | 6.95 | 7.59 |
| A02460 | KEQ79735.1; hypothetical protein M438DRAFT_339518 [ *Aureobasidium* *pullulans* EXF-150] | 0 | 1.85 | 1.56 | 1.73 | 2.37 |
| A02478 | OBW66952.1; FAD/NAD(P)-binding domain-containing protein [ *Aureobasidium* *pullulans*] | 0 | 1.27 | 1.48 | 1.54 | 1.74 |
| A02488 | XP_013338745.1; hypothetical protein AUEXF2481DRAFT_34126 [ *Aureobasidium* *subglaciale* EXF-2481] | 0 | 2.24 | 3.28 | 2.87 | 2.51 |
| A02536 | XP_013429502.1; major facilitator superfamily transporter [ *Aureobasidium* *namibiae* CBS 147.97] | 0 | 1.27 | 0.7 | 0.98 | 0.57 |
| A02544 | XP_013344719.1; hypothetical protein AUEXF2481DRAFT_107439 [ *Aureobasidium* *subglaciale* EXF-2481] | 0 | 1.66 | 1.58 | 2.06 | 1.95 |
| A02557 | XP_013429895.1; hypothetical protein M436DRAFT_80029 [ *Aureobasidium* *namibiae* CBS 147.97] | 0 | 1.06 | 1.08 | 1.46 | 1.18 |
| A02606 | KEQ63746.1; hypothetical protein M437DRAFT_46090, partial [ *Aureobasidium* *melanogenum* CBS 110374] | 0 | 2.12 | 3.02 | 1.87 | 3.96 |
| A02625 | OBW69875.1; D123-domain-containing protein [ *Aureobasidium* *pullulans*] | 0 | 0.82 | 1.17 | 1.43 | 1.19 |
| A02638 | XP_013344505.1; hypothetical protein AUEXF2481DRAFT_28829 [ *Aureobasidium* *subglaciale* EXF-2481] | 0 | 1.74 | 1.32 | 1.53 | 1.31 |
| A02652 | XP_013344735.1; hypothetical protein AUEXF2481DRAFT_662302 [ *Aureobasidium* *subglaciale* EXF-2481] | 0 | 1.72 | 2.54 | 3.03 | 2.62 |
| A02673 | KEQ63927.1; cyanate hydratase [ *Aureobasidium* *melanogenum* CBS 110374] | 0 | 1.74 | 1.8 | 2.32 | 1.49 |
| A02748 | KEQ84542.1; acid phosphatase/Vanadium-dependent haloperoxidase [ *Aureobasidium* *pullulans* EXF-150] | 0 | 0.78 | 1.05 | 1.12 | 0.95 |
| A02751 | XP_013343027.1; hypothetical protein AUEXF2481DRAFT_277771 [ *Aureobasidium* *subglaciale* EXF-2481] | 0 | 3.36 | 2.39 | 2.91 | 1.81 |
| A02784 | OBW68539.1; Uncharacterized protein AUREO_013900 [ *Aureobasidium* *pullulans*] | 0 | 1.18 | 1.77 | 1.52 | 1.33 |
| A02815 | KEQ84475.1; cytidine deaminase-like protein [ *Aureobasidium* *pullulans* EXF-150] | 0 | 2.47 | 2.81 | 2.31 | 2.35 |
| A02823 | KEQ60835.1; enolase C-terminal domain-like protein [ *Aureobasidium* *melanogenum* CBS 110374] | 0 | 1.38 | 1.56 | 2.01 | 1.56 |
| A02826 | XP_013344444.1; phosphoglycerate kinase [ *Aureobasidium* *subglaciale* EXF-2481] | 0 | 2.02 | 2.47 | 2.49 | 1.94 |
| A02835 | KEQ84457.1; hypothetical protein M438DRAFT_273461 [ *Aureobasidium* *pullulans* EXF-150] | 0 | 1.22 | 1.35 | 1 | 0.97 |
| A02839 | KEQ66184.1; NTF2-like protein [ *Aureobasidium* *melanogenum* CBS 110374] | 0 | 3.55 | 1.58 | 1.89 | 2.28 |
| A02867 | KEQ66318.1; oxidoreductase [ *Aureobasidium* *melanogenum* CBS 110374] | 0 | 1.5 | 1.14 | 1.29 | 2.14 |
| A02870 | KEQ66321.1; metal-dependent amidase/aminoacylase/carboxypeptidase [ *Aureobasidium* *melanogenum* CBS 110374] | 0 | 1.55 | 0.83 | 1.24 | 1.37 |
| A02875 | KEQ66325.1; hypothetical protein M437DRAFT_62538 [ *Aureobasidium* *melanogenum* CBS 110374] | 0 | 1.59 | 1.71 | 1.2 | 1.32 |
| A02921 | KEQ66369.1; Ppx-GppA-domain-containing protein [ *Aureobasidium* *melanogenum* CBS 110374] | 0 | 0.72 | 0.84 | 1.14 | 0.96 |
| A02926 | KEQ66372.1; hypothetical protein M437DRAFT_62583 [ *Aureobasidium* *melanogenum* CBS 110374] | 0 | 2.25 | 3.47 | 3.19 | 3.02 |
| A02948 | KEQ66391.1; calcium-dependent phosphotriesterase [ *Aureobasidium* *melanogenum* CBS 110374] | 0 | 2.15 | 2.02 | 2.1 | 1.72 |
| A02961 | KEQ66198.1; hypothetical protein M437DRAFT_62425 [ *Aureobasidium* *melanogenum* CBS 110374] | 0 | 3.34 | 3.97 | 4.65 | 4.65 |
| A02964 | KEQ66409.1; carbon-nitrogen hydrolase [ *Aureobasidium* *melanogenum* CBS 110374] | 0 | 4.91 | 4.37 | 4.58 | 5.77 |
| A02966 | KEQ66411.1; hypothetical protein M437DRAFT_39615 [ *Aureobasidium* *melanogenum* CBS 110374] | 0 | 3.63 | 4.14 | 2.72 | 3.14 |
| A02968 | XP_013426885.1; chaperone HchA [ *Aureobasidium* *namibiae* CBS 147.97] | 0 | 2.53 | 3.28 | 2.96 | 2.33 |
| A02975 | KEQ66420.1; alpha/beta-hydrolase [ *Aureobasidium* *melanogenum* CBS 110374] | 0 | 2.04 | 2.24 | 2.83 | 2.88 |
| A03001 | KEQ66445.1; P-loop containing nucleoside triphosphate hydrolase protein [ *Aureobasidium* *melanogenum* CBS 110374] | 0 | 1.63 | 2.06 | 2.01 | 2.27 |
| A03060 | KEQ66172.1; FAD/NAD(P)-binding domain-containing protein [ *Aureobasidium* *melanogenum* CBS 110374] | 0 | 3.44 | 4.64 | 3.71 | 3.38 |
| A03097 | KEQ66476.1; mitochondrial carrier [ *Aureobasidium* *melanogenum* CBS 110374] | 0 | 1.89 | 2.24 | 2.76 | 2.38 |
| A03110 | KEQ66548.1; hypothetical protein M437DRAFT_62740 [ *Aureobasidium* *melanogenum* CBS 110374] | 0 | 1.54 | 1.85 | 1.86 | 2.13 |
| A03122 | OBW64878.1; Heat shock protein 70 [ *Aureobasidium* *pullulans*] | 0 | 3.48 | 3.08 | 3.39 | 3.9 |
| A03137 | KEQ66569.1; putative nonsense-mediated mRNA decay protein 3 [ *Aureobasidium* *melanogenum* CBS 110374] | 0 | 1.68 | 2.48 | 2.64 | 2.68 |
| A03151 | KEQ66582.1; hypothetical protein M437DRAFT_72146 [ *Aureobasidium* *melanogenum* CBS 110374] | 0 | 1.87 | 2.46 | 2.04 | 1.67 |
| A03190 | KEQ66627.1; hypothetical protein M437DRAFT_62815 [ *Aureobasidium* *melanogenum* CBS 110374] | 0 | 1.83 | 2.44 | 2.67 | 1.95 |
| A03233 | XP_013426779.1; lysophospholipase A [ *Aureobasidium* *namibiae* CBS 147.97] | 0 | 2.77 | 1.75 | 2.75 | 2.6 |
| A03243 | KEQ66680.1; general substrate transporter [ *Aureobasidium* *melanogenum* CBS 110374] | 0 | 1.22 | 1.15 | 1.09 | 1.4 |
| A03282 | KEQ66174.1; aldehyde dehydrogenase [ *Aureobasidium* *melanogenum* CBS 110374] | 0 | 1.6 | 2.32 | 1.98 | 2.08 |
| A03283 | KEQ66227.1; aldehyde dehydrogenase [ *Aureobasidium* *melanogenum* CBS 110374] | 0 | 4.42 | 4.02 | 5.41 | 6.15 |
| A03291 | KEQ66725.1; nucleoside hydrolase [ *Aureobasidium* *melanogenum* CBS 110374] | 0 | 1 | 0.59 | 0.67 | 1.28 |
| A03319 | KEQ66757.1; DNA repair protein [ *Aureobasidium* *melanogenum* CBS 110374] | 0 | 1.52 | 1.84 | 2.04 | 1.91 |
| A03349 | XP_013426865.1; hypothetical protein M436DRAFT_82703 [ *Aureobasidium* *namibiae* CBS 147.97] | 0 | 2.39 | 2.3 | 2.82 | 3.07 |
| A03384 | KEQ66823.1; hypothetical protein M437DRAFT_81298 [ *Aureobasidium* *melanogenum* CBS 110374] | 0 | 3.24 | 3.18 | 3.04 | 3.15 |
| A03396 | KEQ66812.1; hypothetical protein M437DRAFT_72340 [ *Aureobasidium* *melanogenum* CBS 110374] | 0 | 5.86 | 4.67 | 6.51 | 7.97 |
| A03403 | KEQ66803.1; hypothetical protein M437DRAFT_81279 [ *Aureobasidium* *melanogenum* CBS 110374] | 0 | 1.07 | 1.89 | 1.82 | 1.86 |
| A03447 | KEQ66762.1; hypothetical protein M437DRAFT_39421 [ *Aureobasidium* *melanogenum* CBS 110374] | 0 | 3.48 | 4.95 | 5.56 | 5.67 |
| A03461 | KEQ66890.1; kinase-like protein [ *Aureobasidium* *melanogenum* CBS 110374] | 0 | 3.91 | 3.7 | 3.06 | 2.91 |
| A03481 | KEQ66914.1; MFS general substrate transporter [ *Aureobasidium* *melanogenum* CBS 110374] | 0 | 2.1 | 2.69 | 2.35 | 2.49 |
| A03523 | XP_013431270.1; sugar transporter [ *Aureobasidium* *namibiae* CBS 147.97] | 0 | 1.31 | 1.61 | 1.62 | 1.39 |
| A03558 | KEQ66279.1; hypothetical protein M437DRAFT_80775 [ *Aureobasidium* *melanogenum* CBS 110374] | 0 | 2.74 | 1.5 | 1.51 | 1.8 |
| A03565 | XP_013343749.1; hypothetical protein AUEXF2481DRAFT_5060 [ *Aureobasidium* *subglaciale* EXF-2481] | 0 | 1.78 | 1.46 | 1.42 | 0.97 |
| A03570 | KEQ66484.1; hypothetical protein M437DRAFT_72055 [ *Aureobasidium* *melanogenum* CBS 110374] | 0 | 2.9 | 2.02 | 1.32 | 1.7 |
| A03596 | KEQ89339.1; rhamnose mutarotase [ *Aureobasidium* *pullulans* EXF-150] | 0 | 1.45 | 1.06 | 1.49 | 1.89 |
| A03610 | XP_013426779.1; lysophospholipase A [ *Aureobasidium* *namibiae* CBS 147.97] | 0 | 4.44 | 4.42 | 5.67 | 5.68 |
| A03674 | OBW69358.1; Uncharacterized protein AUREO_005640 [ *Aureobasidium* *pullulans*] | 0 | 1.74 | 3.57 | 3.24 | 3.43 |
| A03680 | XP_013345193.1; hypothetical protein AUEXF2481DRAFT_3434 [ *Aureobasidium* *subglaciale* EXF-2481] | 0 | 0.68 | 0.79 | 0.86 | 1.11 |
| A03710 | KEQ89595.1; RmlC-like cupin [ *Aureobasidium* *pullulans* EXF-150] | 0 | 2.14 | 1.62 | 2.03 | 1.55 |
| A03711 | NA | 0 | 1.63 | 2.88 | 3.19 | 2.89 |
| A03743 | KEQ89565.1; WSC-domain-containing protein [ *Aureobasidium* *pullulans* EXF-150] | 0 | 0.77 | 0.79 | 1.15 | 1.07 |
| A03751 | XP_013345256.1; glycoside hydrolase family 43 protein [ *Aureobasidium* *subglaciale* EXF-2481] | 0 | 0.93 | 0.82 | 1.03 | 0.8 |
| A03761 | XP_013425104.1; Isochorismatase hydrolase [ *Aureobasidium* *namibiae* CBS 147.97] | 0 | 2.19 | 2.44 | 2.07 | 1.26 |
| A03770 | KEQ89507.1; hypothetical protein M438DRAFT_286866 [ *Aureobasidium* *pullulans* EXF-150] | 0 | 1.55 | 1.61 | 1.36 | 1.06 |
| A03809 | KEQ89660.1; concanavalin A-like lectin/glucanase [ *Aureobasidium* *pullulans* EXF-150] | 0 | 1.23 | 1.24 | 1.33 | 0.71 |
| A03814 | KEQ89666.1; MFS general substrate transporter [ *Aureobasidium* *pullulans* EXF-150] | 0 | 2.84 | 2.05 | 1.93 | 1.56 |
| A03822 | KEQ89676.1; hypothetical protein M438DRAFT_361135 [ *Aureobasidium* *pullulans* EXF-150] | 0 | 2.19 | 1.52 | 1.72 | 1.91 |
| A03832 | KEQ66805.1; pyruvate kinase [ *Aureobasidium* *melanogenum* CBS 110374] | 0 | 1.41 | 1.98 | 1.64 | 1.64 |
| A03854 | KEQ66802.1; hypothetical protein M437DRAFT_72333 [ *Aureobasidium* *melanogenum* CBS 110374] | 0 | 1.88 | 1.89 | 2.42 | 2.67 |
| A03902 | OBW67057.1; hypothetical protein AUREO_028690 [ *Aureobasidium* *pullulans*] | 0 | 0.92 | 1.38 | 1.2 | 1.12 |
| A03912 | PSK60725.1; Methylsterol monooxygenase [Elsinoe australis] | 0 | 4.12 | 6.4 | 6 | 5.32 |
| A03913 | NA | 0 | 0.82 | 1.19 | 1.2 | 1.05 |
| A03919 | XP_013339154.1; hypothetical protein AUEXF2481DRAFT_48606 [ *Aureobasidium* *subglaciale* EXF-2481] | 0 | 1.3 | 1.61 | 1.91 | 1.36 |
| A03927 | XP_013343890.1; hypothetical protein AUEXF2481DRAFT_46949 [ *Aureobasidium* *subglaciale* EXF-2481] | 0 | 1.16 | 1.53 | 1.44 | 1.36 |
| A03932 | XP_013343933.1; hypothetical protein AUEXF2481DRAFT_79696 [ *Aureobasidium* *subglaciale* EXF-2481] | 0 | 3.7 | 1.85 | 2.64 | 2.54 |
| A03941 | XP_013426886.1; alpha/beta-hydrolase [ *Aureobasidium* *namibiae* CBS 147.97] | 0 | 1.25 | 1.44 | 1.57 | 1.21 |
| A04003 | NA | 0 | 1.74 | 1.36 | 1.64 | 1.01 |
| A04021 | XP_013426729.1; hypothetical protein M436DRAFT_64559 [ *Aureobasidium* *namibiae* CBS 147.97] | 0 | 3 | 3.7 | 3.69 | 4.25 |
| A04024 | XP_013343799.1; hypothetical protein AUEXF2481DRAFT_29486 [ *Aureobasidium* *subglaciale* EXF-2481] | 0 | 1.41 | 0.76 | 0.97 | 0.92 |
| A04026 | KEQ89270.1; hypothetical protein M438DRAFT_308516 [ *Aureobasidium* *pullulans* EXF-150] | 0 | 4.23 | 3.29 | 3.7 | 4.15 |
| A04057 | OBW65452.1; Calcium-transporting ATPase [ *Aureobasidium* *pullulans*] | 0 | 2.87 | 3.61 | 3.15 | 2.25 |
| A04060 | KEQ66222.1; endopolygalacturonase PGb [ *Aureobasidium* *melanogenum* CBS 110374] | 0 | 0.93 | 0.74 | 0.82 | 1.12 |
| A04071 | XP_013343912.1; hypothetical protein AUEXF2481DRAFT_65528 [ *Aureobasidium* *subglaciale* EXF-2481] | 0 | 1.63 | 1.05 | 1.34 | 1.88 |
| A04112 | XP_013345229.1; hypothetical protein AUEXF2481DRAFT_3543 [ *Aureobasidium* *subglaciale* EXF-2481] | 0 | 0.92 | 0.87 | 0.89 | 1.47 |
| A04118 | OBW64878.1; Heat shock protein 70 [ *Aureobasidium* *pullulans*] | 0 | 1.18 | 1.57 | 1.85 | 1.74 |
| A04139 | KEQ66333.1; hypothetical protein M437DRAFT_39656 [ *Aureobasidium* *melanogenum* CBS 110374] | 0 | 2.74 | 3.09 | 3.3 | 2.41 |
| A04173 | KEQ89149.1; hypothetical protein M438DRAFT_1875 [ *Aureobasidium* *pullulans* EXF-150] | 0 | 3.53 | 3.77 | 3.48 | 3.82 |
| A04181 | KEQ66576.1; hypothetical protein M437DRAFT_62768 [ *Aureobasidium* *melanogenum* CBS 110374] | 0 | 1.07 | 1.06 | 1.19 | 0.79 |
| A04182 | XP_013343851.1; hypothetical protein AUEXF2481DRAFT_39885 [ *Aureobasidium* *subglaciale* EXF-2481] | 0 | 0.88 | 1.27 | 1.57 | 1.4 |
| A04192 | KEQ66472.1; putative MFS allantoate transporter [ *Aureobasidium* *melanogenum* CBS 110374] | 0 | 4.34 | 2.92 | 3.06 | 3.68 |
| A04195 | XP_013343920.1; hypothetical protein AUEXF2481DRAFT_65581 [ *Aureobasidium* *subglaciale* EXF-2481] | 0 | 1.4 | 0.88 | 1.18 | 0.93 |
| A04205 | XP_013424166.1; pectin lyase-like protein [ *Aureobasidium* *namibiae* CBS 147.97] | 0 | 2.15 | 3.32 | 3.24 | 3.22 |
| A04226 | KEQ65953.1; 1-aminocyclopropane-1-carboxylate deaminase [ *Aureobasidium* *melanogenum* CBS 110374] | 0 | 7.57 | 6.16 | 6.47 | 6.15 |
| A04230 | KEQ65945.1; hypothetical protein M437DRAFT_63478 [ *Aureobasidium* *melanogenum* CBS 110374] | 0 | 2.7 | 3.53 | 3.36 | 4.46 |
| A04234 | XP_013341400.1; hypothetical protein AUEXF2481DRAFT_68011 [ *Aureobasidium* *subglaciale* EXF-2481] | 0 | 1.47 | 1.43 | 1.42 | 1.45 |
| A04238 | XP_013341463.1; hypothetical protein AUEXF2481DRAFT_366072 [ *Aureobasidium* *subglaciale* EXF-2481] | 0 | 2.37 | 3.15 | 2.89 | 2.41 |
| A04263 | XP_013341303.1; hypothetical protein AUEXF2481DRAFT_7324 [ *Aureobasidium* *subglaciale* EXF-2481] | 0 | 1.42 | 1.78 | 2 | 1.59 |
| A04264 | KEQ66016.1; DUF383-domain-containing protein [ *Aureobasidium* *melanogenum* CBS 110374] | 0 | 1.39 | 2.11 | 2 | 2.07 |
| A04295 | XP_013345634.1; hypothetical protein AUEXF2481DRAFT_2992 [ *Aureobasidium* *subglaciale* EXF-2481] | 0 | 2.44 | 1.72 | 1.93 | 1.85 |
| A04304 | XP_013345747.1; glycoside hydrolase family 28 protein [ *Aureobasidium* *subglaciale* EXF-2481] | 0 | 2.96 | 4.34 | 5.26 | 4.72 |
| A04306 | KEQ88832.1; putative inosine-5'-monophosphate dehydrogenase [ *Aureobasidium* *pullulans* EXF-150] | 0 | 2.13 | 2.81 | 2.42 | 1.76 |
| A04309 | KEQ66019.1; hypothetical protein M437DRAFT_63548 [ *Aureobasidium* *melanogenum* CBS 110374] | 0 | 3.77 | 2.26 | 3.61 | 3.23 |
| A04312 | KEQ65734.1; hypothetical protein M437DRAFT_81587 [ *Aureobasidium* *melanogenum* CBS 110374] | 0 | 1.72 | 1.85 | 1.45 | 2.09 |
| A04330 | XP_013424037.1; hypothetical protein M436DRAFT_54540 [ *Aureobasidium* *namibiae* CBS 147.97] | 0 | 2.06 | 2.28 | 1.72 | 2.21 |
| A04339 | OBW64599.1; Uncharacterized protein AUREO_053360 [ *Aureobasidium* *pullulans*] | 0 | 1.27 | 1.4 | 1.86 | 1.61 |
| A04347 | KEQ65983.1; APG17-domain-containing protein [ *Aureobasidium* *melanogenum* CBS 110374] | 0 | 1.22 | 0.87 | 0.95 | 0.89 |
| A04355 | KEQ65907.1; hypothetical protein M437DRAFT_63445 [ *Aureobasidium* *melanogenum* CBS 110374] | 0 | 1.81 | 3.07 | 2.91 | 3.37 |
| A04374 | OBW67126.1; DUF427-domain-containing protein [ *Aureobasidium* *pullulans*] | 0 | 3.14 | 3.38 | 3.91 | 4.44 |
| A04393 | KEQ65701.1; hypothetical protein M437DRAFT_41358 [ *Aureobasidium* *melanogenum* CBS 110374] | 0 | 0.95 | 1.22 | 1.2 | 0.99 |
| A04428 | XP_013345660.1; glycoside hydrolase family 43 protein [ *Aureobasidium* *subglaciale* EXF-2481] | 0 | 1.95 | 2.61 | 2.51 | 1.89 |
| A04432 | KEQ64126.1; MFS general substrate transporter [ *Aureobasidium* *melanogenum* CBS 110374] | 0 | 1.29 | 2.38 | 1.8 | 2.14 |
| A04434 | XP_013345668.1; hypothetical protein AUEXF2481DRAFT_3125 [ *Aureobasidium* *subglaciale* EXF-2481] | 0 | 3.52 | 3.77 | 3.96 | 4.53 |
| A04466 | KEQ88589.1; cat eye syndrome critical region protein 5 precursor [ *Aureobasidium* *pullulans*] | 0 | 1.59 | 1.68 | 1.88 | 2.13 |
| A04472 | KEQ88582.1; hypothetical protein M438DRAFT_351826 [ *Aureobasidium* *pullulans* EXF-150] | 0 | 1.4 | 0.91 | 1.09 | 1.62 |
| A04475 | NA | 0 | 2.58 | 2.78 | 2.4 | 1.99 |
| A04550 | XP_013424734.1; gluconate 5-dehydrogenase [ *Aureobasidium* *namibiae* CBS 147.97] | 0 | 1.66 | 1.18 | 2.27 | 2.17 |
| A04588 | KEQ88515.1; hypothetical protein M438DRAFT_85875 [ *Aureobasidium* *pullulans* EXF-150] | 0 | 1.78 | 2.32 | 2.66 | 3 |
| A04602 | XP_013346058.1; hypothetical protein AUEXF2481DRAFT_596672 [ *Aureobasidium* *subglaciale* EXF-2481] | 0 | 2.21 | 2.37 | 2.03 | 1.47 |
| A04620 | XP_013345893.1; hypothetical protein AUEXF2481DRAFT_37949 [ *Aureobasidium* *subglaciale* EXF-2481] | 0 | 2.16 | 2.27 | 2.51 | 3.19 |
| A04631 | KEQ64284.1; hypothetical protein M437DRAFT_45425 [ *Aureobasidium* *melanogenum* CBS 110374] | 0 | 2.1 | 2.07 | 2.24 | 2.32 |
| A04651 | KEQ64308.1; transcription initiation factor IIA, gamma subunit [ *Aureobasidium* *melanogenum* CBS 110374] | 0 | 0.78 | 1.43 | 1.43 | 1.38 |
| A04664 | XP_013341360.1; hypothetical protein AUEXF2481DRAFT_42574 [ *Aureobasidium* *subglaciale* EXF-2481] | 0 | 0.84 | 1.27 | 1.48 | 1.37 |
| A04668 | XP_013345818.1; hypothetical protein AUEXF2481DRAFT_27761 [ *Aureobasidium* *subglaciale* EXF-2481] | 0 | 1.81 | 1.66 | 1.36 | 0.89 |
| A04697 | NA | 0 | 1.42 | 1.94 | 2.46 | 2.32 |
| A04708 | KFY14963.1; hypothetical protein V491_05836 [Pseudogymnoascus sp. VKM F-3775] | 0 | 2.74 | 2.42 | 3.5 | 3.14 |
| A04715 | XP_013422523.1; DUF1479-domain-containing protein [ *Aureobasidium* *namibiae* CBS 147.97] | 0 | 1.21 | 0.65 | 1.03 | 1.25 |
| A04717 | NA | 0 | 2.54 | 2.27 | 2.03 | 1.69 |
| A04740 | KEQ79193.1; hypothetical protein M438DRAFT_151704 [ *Aureobasidium* *pullulans* EXF-150] | 0 | 2.3 | 3.25 | 3.09 | 2.67 |
| A04742 | KEQ78329.1; multidrug resistance protein-like protein [ *Aureobasidium* *pullulans* EXF-150] | 0 | 2.37 | 4.05 | 4.08 | 3.71 |
| A04744 | KEQ64462.1; hypothetical protein M437DRAFT_45301 [ *Aureobasidium* *melanogenum* CBS 110374] | 0 | 2 | 2.73 | 2.69 | 2.32 |
| A04752 | KEQ79667.1; NAD(P)-binding protein [ *Aureobasidium* *pullulans* EXF-150] | 0 | 1.41 | 1.79 | 1.64 | 1.39 |
| A04753 | KEQ66065.1; hypothetical protein M437DRAFT_40399 [ *Aureobasidium* *melanogenum* CBS 110374] | 0 | 0.74 | 1.19 | 1.15 | 1.13 |
| A04761 | KEQ65988.1; Formate/nitrite transporter [ *Aureobasidium* *melanogenum* CBS 110374] | 0 | 4.51 | 4.83 | 5.67 | 6.75 |
| A04771 | KEQ89016.1; cysteine proteinase [ *Aureobasidium* *pullulans* EXF-150] | 0 | 1.5 | 2.03 | 2.18 | 2.14 |
| A04773 | XP_013341273.1; hypothetical protein AUEXF2481DRAFT_7161 [ *Aureobasidium* *subglaciale* EXF-2481] | 0 | 1.41 | 1.18 | 1.37 | 1.38 |
| A04797 | KEQ58240.1; alpha/beta-hydrolase [ *Aureobasidium* *melanogenum* CBS 110374] | 0 | 2.38 | 2.81 | 2.58 | 1.86 |
| A04817 | KEQ60856.1; hypothetical protein M437DRAFT_53228 [ *Aureobasidium* *melanogenum* CBS 110374] | 0 | 1.68 | 2 | 1.78 | 1.38 |
| A04856 | XP_013430191.1; Arylsulphatase [ *Aureobasidium* *namibiae* CBS 147.97] | 0 | 1.26 | 2.01 | 1.47 | 2.04 |
| A04893 | KEQ64971.1; fatty-acid amide hydrolase [ *Aureobasidium* *melanogenum* CBS 110374] | 0 | 3.16 | 2.52 | 2.51 | 2.87 |
| A04915 | KEQ58398.1; adenosine deaminase [ *Aureobasidium* *melanogenum* CBS 110374] | 0 | 1.55 | 1.92 | 2.14 | 1.59 |
| A04967 | KEQ58309.1; hypothetical protein M437DRAFT_59716 [ *Aureobasidium* *melanogenum* CBS 110374] | 0 | 2.36 | 2.32 | 2.36 | 1.58 |
| A04975 | XP_013430283.1; P-loop containing nucleoside triphosphate hydrolase protein [ *Aureobasidium* *namibiae* CBS 147.97] | 0 | 1.35 | 0.8 | 0.96 | 1.05 |
| A04985 | KEQ58429.1; Metallo-hydrolase/oxidoreductase [ *Aureobasidium* *melanogenum* CBS 110374] | 0 | 2.87 | 2.2 | 3.19 | 4.02 |
| A04990 | KEQ58435.1; hypothetical protein M437DRAFT_69957 [ *Aureobasidium* *melanogenum* CBS 110374] | 0 | 2.58 | 2.91 | 3.29 | 3.91 |
| A05007 | KEQ58457.1; hypothetical protein M437DRAFT_88604 [ *Aureobasidium* *melanogenum* CBS 110374] | 0 | 2.63 | 3.3 | 2.74 | 2.04 |
| A05041 | XP_013422378.1; hypothetical protein M436DRAFT_86608 [ *Aureobasidium* *namibiae* CBS 147.97] | 0 | 1.21 | 0.87 | 1.19 | 1.43 |
| A05055 | KKP05995.1; hypothetical protein THAR02_01867 [Trichoderma harzianum] | 0 | 3.64 | 1.88 | 3.48 | 2.82 |
| A05078 | KIK58096.1; hypothetical protein GYMLUDRAFT_706765 [Gymnopus luxurians FD-317 M1] | 0 | 6.5 | 8.51 | 8.05 | 8.04 |
| A05080 | KEQ62000.1; amino acid transporter [ *Aureobasidium* *melanogenum* CBS 110374] | 0 | 3.51 | 4.97 | 4.33 | 4.05 |
| A05084 | KEQ80879.1; hypothetical protein M438DRAFT_358485 [ *Aureobasidium* *pullulans* EXF-150] | 0 | 3.49 | 3.85 | 5.34 | 4.78 |
| A05114 | KEQ61955.1; hypothetical protein M437DRAFT_50596 [ *Aureobasidium* *melanogenum* CBS 110374] | 0 | 2.42 | 2.72 | 2.77 | 2.38 |
| A05125 | KEQ61935.1; hypothetical protein M437DRAFT_85371 [ *Aureobasidium* *melanogenum* CBS 110374] | 0 | 3.46 | 3.39 | 4.64 | 4.59 |
| A05136 | KEQ61921.1; NAD(P)-binding protein [ *Aureobasidium* *melanogenum* CBS 110374] | 0 | 1.38 | 1.34 | 1.43 | 0.82 |
| A05148 | XP_013422766.1; hypothetical protein M436DRAFT_86261 [ *Aureobasidium* *namibiae* CBS 147.97] | 0 | 3.79 | 5.17 | 5.13 | 4.12 |
| A05173 | KEQ61886.1; hypothetical protein M437DRAFT_76055 [ *Aureobasidium* *melanogenum* CBS 110374] | 0 | 2.5 | 3.7 | 4.55 | 4.05 |
| A05187 | KEQ61872.1; hypothetical protein M437DRAFT_76042 [ *Aureobasidium* *melanogenum* CBS 110374] | 0 | 0.89 | 1.28 | 0.98 | 0.91 |
| A05194 | KEQ61863.1; PEBP-like protein [ *Aureobasidium* *melanogenum* CBS 110374] | 0 | 1.51 | 1.73 | 2.11 | 2.39 |
| A05195 | XP_023628126.1; uncharacterized protein RCC_07100 [Ramularia collo-cygni] | 0 | 3.24 | 5.43 | 5.38 | 4.85 |
| A05200 | KEQ61941.1; putative mitochondrial ornithine carrier protein AmcA/Ort1 [ *Aureobasidium* *melanogenum* CBS 110374] | 0 | 2.48 | 2.85 | 3.24 | 2.09 |
| A05207 | KEQ61944.1; hypothetical protein M437DRAFT_50908 [ *Aureobasidium* *melanogenum* CBS 110374] | 0 | 2.43 | 1.66 | 2.19 | 1.83 |
| A05210 | KEQ61856.1; hypothetical protein M437DRAFT_76029 [ *Aureobasidium* *melanogenum* CBS 110374] | 0 | 2 | 1.26 | 1.58 | 2.77 |
| A05243 | KEQ61816.1; hypothetical protein M437DRAFT_50549 [ *Aureobasidium* *melanogenum* CBS 110374] | 0 | 2.83 | 1.59 | 2.07 | 2.55 |
| A05279 | KEQ65002.1; hypothetical protein M437DRAFT_82989 [ *Aureobasidium* *melanogenum* CBS 110374] | 0 | 1.31 | 1.56 | 1.66 | 1.9 |
| A05302 | KEQ64978.1; hypothetical protein M437DRAFT_44489 [ *Aureobasidium* *melanogenum* CBS 110374] | 0 | 0.96 | 1.18 | 1.29 | 0.88 |
| A05314 | KEQ84991.1; glycoside hydrolase [ *Aureobasidium* *pullulans* EXF-150] | 0 | 2.17 | 2.29 | 2.19 | 1.55 |
| A05315 | KEQ60874.1; hypothetical protein M437DRAFT_53115 [ *Aureobasidium* *melanogenum* CBS 110374] | 0 | 3.25 | 4.63 | 5.46 | 5.18 |
| A05374 | KEQ64865.1; hypothetical protein M437DRAFT_73783 [ *Aureobasidium* *melanogenum* CBS 110374] | 0 | 3.73 | 3.87 | 5.09 | 5.31 |
| A05387 | KEQ64879.1; mRNA turnover protein-like protein 4 [ *Aureobasidium* *melanogenum* CBS 110374] | 0 | 3.4 | 4.4 | 4.27 | 3.56 |
| A05402 | KEQ64889.1; hypothetical protein M437DRAFT_43804 [ *Aureobasidium* *melanogenum* CBS 110374] | 0 | 2.05 | 2.41 | 2.22 | 2.15 |
| A05407 | KEQ64884.1; kinase-like protein [ *Aureobasidium* *melanogenum* CBS 110374] | 0 | 0.89 | 0.75 | 1.24 | 1.24 |
| A05458 | KEQ60241.1; A cutinase-like protein from cryptococcus Sp [ *Aureobasidium* *melanogenum* CBS 110374] | 0 | 2.94 | 2.1 | 2.73 | 3.17 |
| A05470 | KEQ60258.1; alpha-galactosidase [ *Aureobasidium* *melanogenum* CBS 110374] | 0 | 1.86 | 1.73 | 2.33 | 2.65 |
| A05477 | KKY26252.1; putative fad binding domain protein [Phaeomoniella chlamydospora] | 0 | 1.28 | 0.75 | 1.42 | 1.28 |
| A05481 | XP_014659318.1; conserved hypothetical protein [Moesziomyces antarcticus] | 0 | 1.26 | 1.39 | 1.24 | 1.16 |
| A05499 | KEQ60269.1; hypothetical protein M437DRAFT_54698 [ *Aureobasidium* *melanogenum* CBS 110374] | 0 | 2.31 | 1.58 | 1.43 | 2.9 |
| A05508 | KEQ60259.1; acyl-CoA-dependent ceramide synthase [ *Aureobasidium* *melanogenum* CBS 110374] | 0 | 1.56 | 1.82 | 1.56 | 2.03 |
| A05528 | KEQ60429.1; FAS1 domain-containing protein [ *Aureobasidium* *melanogenum* CBS 110374] | 0 | 0.99 | 1.03 | 1.17 | 1.06 |
| A05547 | KEQ60409.1; hypothetical protein M437DRAFT_77479 [ *Aureobasidium* *melanogenum* CBS 110374] | 0 | 4.8 | 5.93 | 5.73 | 5.12 |
| A05566 | KEQ88269.1; hypothetical protein M438DRAFT_80450 [ *Aureobasidium* *pullulans*] | 0 | 1.49 | 2.09 | 2.07 | 1.75 |
| A05627 | KEQ60333.1; alpha/beta hydrolase BEM46/Esterase/lipase/thioesterase [ *Aureobasidium* *melanogenum* CBS 110374] | 0 | 1.02 | 0.6 | 0.72 | 0.87 |
| A05668 | KEQ61767.1; SET domain-containing protein [ *Aureobasidium* *melanogenum* CBS 110374] | 0 | 0.99 | 1.18 | 1.22 | 1.11 |
| A05673 | KEQ60307.1; OPT superfamily oligopeptide transporter [ *Aureobasidium* *melanogenum* CBS 110374] | 0 | 1.15 | 1.77 | 1.54 | 1.41 |
| A05676 | KEQ60310.1; hypothetical protein M437DRAFT_54671 [ *Aureobasidium* *melanogenum* CBS 110374] | 0 | 1.15 | 1.41 | 1.82 | 1.73 |
| A05685 | OBW65140.1; hypothetical protein AUREO_047910 [ *Aureobasidium* *pullulans*] | 0 | 1.07 | 1.66 | 1.75 | 1.65 |
| A05686 | OTA59660.1; transferase [Hypoxylon sp. EC38] | 0 | 1.25 | 1.37 | 1.31 | 1.91 |
| A05687 | NA | 0 | 2.04 | 1.64 | 1.13 | 1.61 |
| A05715 | NA | 0 | 2.84 | 4.74 | 3.27 | 5.5 |
| A05723 | XP_013344211.1; hypothetical protein AUEXF2481DRAFT_39584 [ *Aureobasidium* *subglaciale* EXF-2481] | 0 | 2.86 | 3.59 | 2.73 | 2.65 |
| A05724 | KEQ61633.1; putative 2-hydroxychromene-2-carboxylate isomerase [ *Aureobasidium* *melanogenum* CBS 110374] | 0 | 2.36 | 2.14 | 3.04 | 2.93 |
| A05788 | KEQ61703.1; HHE domain protein [ *Aureobasidium* *melanogenum* CBS 110374] | 0 | 4.22 | 5.97 | 6.63 | 6.43 |
| A05790 | KEQ61706.1; glycoside hydrolase [ *Aureobasidium* *melanogenum* CBS 110374] | 0 | 1.28 | 1.16 | 1.53 | 1.04 |
| A05803 | KEQ61718.1; hypothetical protein M437DRAFT_85668 [ *Aureobasidium* *melanogenum* CBS 110374] | 0 | 4.77 | 5.71 | 5.35 | 4.38 |
| A05871 | KEQ58508.1; hypothetical protein M437DRAFT_59197 [ *Aureobasidium* *melanogenum* CBS 110374] | 0 | 1.34 | 1.5 | 1.8 | 1.46 |
| A05885 | KEQ58494.1; putative cytochrome P450 [ *Aureobasidium* *melanogenum* CBS 110374] | 0 | 3.42 | 2.87 | 2.59 | 2.73 |
| A05892 | OBW69240.1; hypothetical protein AUREO_006760 [ *Aureobasidium* *pullulans*] | 0 | 1.84 | 2.84 | 3 | 2.52 |
| A05912 | KEQ58097.1; Arginase/deacetylase [ *Aureobasidium* *melanogenum* CBS 110374] | 0 | 1.39 | 1.54 | 1.66 | 1.11 |
| A05919 | KEQ58090.1; oxidoreductase [ *Aureobasidium* *melanogenum* CBS 110374] | 0 | 1.22 | 0.83 | 1.14 | 1.4 |
| A05920 | KEQ83022.1; hypothetical protein M438DRAFT_336372 [ *Aureobasidium* *pullulans* EXF-150] | 0 | 1.49 | 1.07 | 0.93 | 1.07 |
| A05922 | KEQ83102.1; carboxylesterase family protein [ *Aureobasidium* *pullulans* EXF-150] | 0 | 2.31 | 2.23 | 1.86 | 1.58 |
| A05934 | KEQ61558.1; hypothetical protein M437DRAFT_51300, partial [ *Aureobasidium* *melanogenum* CBS 110374] | 0 | 2.32 | 1.94 | 2.73 | 2.52 |
| A05969 | OBW64091.1; Uncharacterized protein AUREO_058430 [ *Aureobasidium* *pullulans*] | 0 | 4.51 | 3.92 | 2.64 | 3.64 |
| A05988 | KEQ58067.1; hypothetical protein M437DRAFT_70249 [ *Aureobasidium* *melanogenum* CBS 110374] | 0 | 3.14 | 5.14 | 4.67 | 4.54 |
| A05999 | KEQ58081.1; hypothetical protein M437DRAFT_60165 [ *Aureobasidium* *melanogenum* CBS 110374] | 0 | 1.19 | 1.72 | 1.29 | 1.37 |
| A06000 | KEQ58542.1; sugar transporter-like protein [ *Aureobasidium* *melanogenum* CBS 110374] | 0 | 2.24 | 2.57 | 1.84 | 1.8 |
| A06024 | XP_013426352.1; hypothetical protein M436DRAFT_65032 [ *Aureobasidium* *namibiae* CBS 147.97] | 0 | 3.05 | 2.84 | 3.2 | 3.95 |
| A06048 | OBW69403.1; Uncharacterized protein AUREO_005120 [ *Aureobasidium* *pullulans*] | 0 | 1.37 | 1.12 | 1.66 | 1.84 |
| A06055 | OBW69407.1; Na+/H+ antiporter [ *Aureobasidium* *pullulans*] | 0 | 1.08 | 1.08 | 1.12 | 1.11 |
| A06057 | OBW69409.1; Uncharacterized protein AUREO_005180 [ *Aureobasidium* *pullulans*] | 0 | 1.12 | 1.86 | 1.67 | 1.59 |
| A06074 | KEQ63161.1; siroheme synthase [ *Aureobasidium* *melanogenum* CBS 110374] | 0 | 2.67 | 3.22 | 3.22 | 3.14 |
| A06087 | KEQ63173.1; ATP-dependent rRNA helicase SPB4 [ *Aureobasidium* *melanogenum* CBS 110374] | 0 | 1.83 | 2.78 | 2.78 | 2.31 |
| A06101 | NA | 0 | 0.79 | 1.3 | 1.54 | 1.36 |
| A06120 | KEQ87347.1; glycoside hydrolase family 72 protein [ *Aureobasidium* *pullulans*] | 0 | 1.2 | 1.05 | 1.22 | 1.4 |
| A06126 | KEQ80060.1; hypothetical protein M438DRAFT_349302 [ *Aureobasidium* *pullulans* EXF-150] | 0 | 1.42 | 1.31 | 1.82 | 2.18 |
| A06128 | XP_013342638.1; hypothetical protein AUEXF2481DRAFT_66886 [ *Aureobasidium* *subglaciale* EXF-2481] | 0 | 4.2 | 4.42 | 4.07 | 4.48 |
| A06164 | KEQ80022.1; aquaporin-like protein [ *Aureobasidium* *pullulans*] | 0 | 1.08 | 1.4 | 1.73 | 1.34 |
| A06168 | XP_013342478.1; hypothetical protein AUEXF2481DRAFT_6213 [ *Aureobasidium* *subglaciale* EXF-2481] | 0 | 1.68 | 1.95 | 2.25 | 2.11 |
| A06186 | XP_013428667.1; hypothetical protein M436DRAFT_62941 [ *Aureobasidium* *namibiae* CBS 147.97] | 0 | 1.26 | 1.23 | 1.3 | 0.82 |
| A06241 | KEQ86948.1; MFS general substrate transporter [ *Aureobasidium* *pullulans* EXF-150] | 0 | 2.4 | 3.35 | 3.5 | 4.18 |
| A06244 | XP_013346681.1; hypothetical protein AUEXF2481DRAFT_2513 [ *Aureobasidium* *subglaciale* EXF-2481] | 0 | 1.02 | 0.83 | 0.63 | 0.64 |
| A06245 | XP_013346682.1; hypothetical protein AUEXF2481DRAFT_2516 [ *Aureobasidium* *subglaciale* EXF-2481] | 0 | 3.14 | 4.19 | 3.89 | 3.55 |
| A06248 | XP_013428409.1; hypothetical protein M436DRAFT_43514 [ *Aureobasidium* *namibiae* CBS 147.97] | 0 | 2.02 | 2.36 | 2.42 | 2.77 |
| A06251 | KEQ60162.1; hypothetical protein M437DRAFT_77596 [ *Aureobasidium* *melanogenum* CBS 110374] | 0 | 1.24 | 1.35 | 1.56 | 1.23 |
| A06255 | XP_013346692.1; glycoside hydrolase family 45 protein [ *Aureobasidium* *subglaciale* EXF-2481] | 0 | 0.66 | 0.7 | 0.8 | 1.01 |
| A06282 | KEQ60160.1; hypothetical protein M437DRAFT_86997 [ *Aureobasidium* *melanogenum* CBS 110374] | 0 | 1.35 | 1.84 | 1.73 | 1.49 |
| A06314 | XP_013347064.1; hypothetical protein AUEXF2481DRAFT_482058 [ *Aureobasidium* *subglaciale* EXF-2481] | 0 | 1.38 | 0.62 | 0.82 | 1.15 |
| A06324 | KEQ60222.1; NAD(P)-binding protein [ *Aureobasidium* *melanogenum* CBS 110374] | 0 | 1.85 | 1.5 | 2.01 | 2.15 |
| A06343 | KEQ87052.1; hypothetical protein M438DRAFT_395298 [ *Aureobasidium* *pullulans* EXF-150] | 0 | 1.7 | 1.9 | 1.99 | 2.19 |
| A06345 | KEQ60183.1; DUF125-domain-containing protein [ *Aureobasidium* *melanogenum* CBS 110374] | 0 | 2.37 | 2.89 | 3.35 | 3.68 |
| A06346 | KEQ60182.1; alpha/beta-hydrolase [ *Aureobasidium* *melanogenum* CBS 110374] | 0 | 3.43 | 4.21 | 3.29 | 3.32 |
| A06363 | KEQ59204.1; hypothetical protein M437DRAFT_87818 [ *Aureobasidium* *melanogenum* CBS 110374] | 0 | 2.24 | 2.31 | 2.87 | 3.13 |
| A06367 | XP_013339318.1; hypothetical protein AUEXF2481DRAFT_70724 [ *Aureobasidium* *subglaciale* EXF-2481] | 0 | 1.28 | 1.52 | 1.8 | 2.08 |
| A06387 | KEQ87017.1; Na+/solute symporter [ *Aureobasidium* *pullulans*] | 0 | 4.55 | 3.54 | 4.65 | 5.34 |
| A06397 | XP_013346879.1; hypothetical protein AUEXF2481DRAFT_37014 [ *Aureobasidium* *subglaciale* EXF-2481] | 0 | 1.62 | 1.89 | 2.5 | 2.12 |
| A06402 | XP_013346924.1; hypothetical protein AUEXF2481DRAFT_46117 [ *Aureobasidium* *subglaciale* EXF-2481] | 0 | 0.99 | 1.11 | 0.66 | 0.88 |
| A06418 | KEQ59308.1; hypothetical protein M437DRAFT_57525 [ *Aureobasidium* *melanogenum* CBS 110374] | 0 | 3.55 | 2.92 | 3.02 | 2.78 |
| A06428 | KEQ61493.1; hypothetical protein M437DRAFT_51775 [ *Aureobasidium* *melanogenum* CBS 110374] | 0 | 0.85 | 0.87 | 1.03 | 1.18 |
| A06431 | OCK85268.1; GABA permease [Lepidopterella palustris CBS 459.81] | 0 | 1.56 | 1.7 | 1.5 | 2.33 |
| A06433 | KUM64657.1; hypothetical protein ACN42_g2409 [Penicillium freii] | 0 | 3.9 | 3.12 | 2.98 | 4.13 |
| A06445 | KEQ59149.1; hypothetical protein M437DRAFT_57311 [ *Aureobasidium* *melanogenum* CBS 110374] | 0 | 1.43 | 2.02 | 2.6 | 2.34 |
| A06459 | EER41964.1; 2-nitropropane dioxygenase [Histoplasma capsulatum H143] | 0 | 2.05 | 1.69 | 1.71 | 1.19 |
| A06460 | XP_013347099.1; hypothetical protein AUEXF2481DRAFT_494178 [ *Aureobasidium* *subglaciale* EXF-2481] | 0 | 1.41 | 0.91 | 0.87 | 0.82 |
| A06471 | XP_013339323.1; hypothetical protein AUEXF2481DRAFT_70795 [ *Aureobasidium* *subglaciale* EXF-2481] | 0 | 1.11 | 0.74 | 1.04 | 1.56 |
| A06474 | XP_013339338.1; hypothetical protein AUEXF2481DRAFT_527677 [ *Aureobasidium* *subglaciale* EXF-2481] | 0 | 1.28 | 1.73 | 1.73 | 2.04 |
| A06476 | KEQ86835.1; peptide-N4-(N-acetyl-beta-D-glucosaminyl) asparaginase amidase N [ *Aureobasidium* *pullulans* EXF-150] | 0 | 4.48 | 3.51 | 2.75 | 2.64 |
| A06477 | XP_013422626.1; ribonuclease Trv, RNase Trv [ *Aureobasidium* *namibiae* CBS 147.97] | 0 | 2.02 | 2.69 | 1.79 | 2.18 |
| A06524 | KEQ79983.1; D-hydantoinase [ *Aureobasidium* *pullulans* EXF-150] | 0 | 2.74 | 2.12 | 1.62 | 1.81 |
| A06526 | OBW65342.1; Nitric oxide synthase-interacting protein [ *Aureobasidium* *pullulans*] | 0 | 2.23 | 1.71 | 2.45 | 2.23 |
| A06569 | XP_013342063.1; hypothetical protein AUEXF2481DRAFT_41790 [ *Aureobasidium* *subglaciale* EXF-2481] | 0 | 1.11 | 0.96 | 1.33 | 1.59 |
| A06573 | ARG41451.1; beta-fructofuranosidase [ *Aureobasidium* *melanogenum*] | 0 | 2.3 | 1.84 | 2.16 | 2.75 |
| A06596 | KEQ67457.1; hypothetical protein M437DRAFT_37364 [ *Aureobasidium* *melanogenum* CBS 110374] | 0 | 1.8 | 2.71 | 3.29 | 2.76 |
| A06607 | KEQ67467.1; carboxyl transferase [ *Aureobasidium* *melanogenum* CBS 110374] | 0 | 2.03 | 1.14 | 2.08 | 1.81 |
| A06646 | KEQ67504.1; uricase [ *Aureobasidium* *melanogenum* CBS 110374] | 0 | 0.68 | 0.84 | 1.07 | 0.97 |
| A06653 | KEQ67512.1; hypothetical protein M437DRAFT_61911 [ *Aureobasidium* *melanogenum* CBS 110374] | 0 | 1.41 | 1.19 | 1.16 | 1.1 |
| A06670 | KEQ67212.1; lipase 3 precursor [ *Aureobasidium* *melanogenum* CBS 110374] | 0 | 2.64 | 1.91 | 3.29 | 3.29 |
| A06692 | KEQ67185.1; RmlC-like cupin [ *Aureobasidium* *melanogenum* CBS 110374] | 0 | 1.47 | 2.04 | 1.61 | 1.35 |
| A06770 | XP_008023243.1; hypothetical protein SETTUDRAFT_105256 [Exserohilum turcica Et28A] | 0 | 1.3 | 1.42 | 1.55 | 2.06 |
| A06818 | KEQ67068.1; hypothetical protein M437DRAFT_18268, partial [ *Aureobasidium* *melanogenum* CBS 110374] | 0 | 0.87 | 1.14 | 1 | 1.02 |
| A06820 | KEQ67066.1; hypothetical protein M437DRAFT_36266 [ *Aureobasidium* *melanogenum* CBS 110374] | 0 | 1.67 | 1.88 | 1.95 | 1.93 |
| A06826 | XP_013424416.1; Formate/nitrite transporter [ *Aureobasidium* *namibiae* CBS 147.97] | 0 | 2.98 | 4.61 | 5.47 | 5.02 |
| A06831 | KEQ82802.1; glycoside hydrolase [ *Aureobasidium* *pullulans* EXF-150] | 0 | 1.43 | 1.43 | 1.58 | 2.05 |
| A06861 | KEQ67023.1; FAD dependent oxidoreductase [ *Aureobasidium* *melanogenum* CBS 110374] | 0 | 1.33 | 2.51 | 2.21 | 2.47 |
| A06900 | KEQ62511.1; RNA-directed RNA polymerase [ *Aureobasidium* *melanogenum* CBS 110374] | 0 | 1.94 | 2.78 | 2.92 | 2.97 |
| A06912 | KEQ82681.1; glucosamine-6-phosphate isomerase [ *Aureobasidium* *pullulans* EXF-150] | 0 | 1.31 | 0.89 | 1.15 | 1.05 |
| A06923 | KEQ62462.1; hypothetical protein M437DRAFT_75602 [ *Aureobasidium* *melanogenum* CBS 110374] | 0 | 0.75 | 1.19 | 0.99 | 1.06 |
| A06954 | KEQ62492.1; putative inorganic phosphate transporter PHO84 [ *Aureobasidium* *melanogenum* CBS 110374] | 0 | 1.16 | 1.46 | 0.89 | 1.33 |
| A06968 | KEQ66980.1; hypothetical protein M437DRAFT_36985 [ *Aureobasidium* *melanogenum* CBS 110374] | 0 | 2.36 | 3.05 | 2.83 | 2.34 |
| A06975 | KEQ66974.1; hypothetical protein M437DRAFT_35805 [ *Aureobasidium* *melanogenum* CBS 110374] | 0 | 1.01 | 1.51 | 1.3 | 1.24 |
| A06978 | KEQ66971.1; hypothetical protein M437DRAFT_37117 [ *Aureobasidium* *melanogenum* CBS 110374] | 0 | 1.46 | 1.45 | 1.6 | 1.41 |
| A06980 | KEQ66968.1; carboxylesterase type B [ *Aureobasidium* *melanogenum* CBS 110374] | 0 | 1.26 | 1.6 | 1.93 | 2.02 |
| A06981 | XP_013424423.1; hypothetical protein M436DRAFT_84731 [ *Aureobasidium* *namibiae* CBS 147.97] | 0 | 1.78 | 2.26 | 1.72 | 2.48 |
| A06993 | KEQ59396.1; Thi4-domain-containing protein [ *Aureobasidium* *melanogenum* CBS 110374] | 0 | 3.53 | 3.21 | 3.08 | 2.26 |
| A07003 | KEQ66955.1; hypothetical protein M437DRAFT_36719 [ *Aureobasidium* *melanogenum* CBS 110374] | 0 | 1.81 | 1.43 | 1.04 | 2.09 |
| A07005 | KEQ66956.1; hypothetical protein M437DRAFT_37693 [ *Aureobasidium* *melanogenum* CBS 110374] | 0 | 2.5 | 2.01 | 2.09 | 2.76 |
| A07006 | KEQ66957.1; general substrate transporter [ *Aureobasidium* *melanogenum* CBS 110374] | 0 | 0.95 | 1.27 | 1.57 | 1.43 |
| A07057 | XP_013423506.1; hypothetical protein M436DRAFT_67282 [ *Aureobasidium* *namibiae* CBS 147.97] | 0 | 1.7 | 2.85 | 2.45 | 2.29 |
| A07060 | KEQ62561.1; Malate/L-lactate dehydrogenase [ *Aureobasidium* *melanogenum* CBS 110374] | 0 | 1.56 | 2.12 | 1.7 | 1.41 |
| A07061 | KEQ62562.1; hypothetical protein M437DRAFT_66417 [ *Aureobasidium* *melanogenum* CBS 110374] | 0 | 3.91 | 4.36 | 4.47 | 4.73 |
| A07062 | KEQ62563.1; dihydroxy-acid and 6-phosphogluconate dehydratase [ *Aureobasidium* *melanogenum* CBS 110374] | 0 | 1.09 | 1.57 | 1.64 | 1.33 |
| A07072 | KEQ62572.1; nitrite transporter [ *Aureobasidium* *melanogenum* CBS 110374] | 0 | 2.98 | 2.89 | 3.13 | 3.27 |
| A07082 | KEQ62582.1; hypothetical protein M437DRAFT_66436 [ *Aureobasidium* *melanogenum* CBS 110374] | 0 | 1.41 | 1.35 | 1.45 | 1.57 |
| A07095 | NA | 0 | 1.58 | 2.12 | 2.2 | 2.2 |
| A07107 | KEQ62609.1; hypothetical protein M437DRAFT_84931 [ *Aureobasidium* *melanogenum* CBS 110374] | 0 | 1.58 | 1.73 | 1.62 | 1.81 |
| A07131 | KEQ62631.1; tannase and feruloyl esterase [ *Aureobasidium* *melanogenum* CBS 110374] | 0 | 1.53 | 1.9 | 1.77 | 1.46 |
| A07183 | KEQ59374.1; NAD-dependent glutamate dehydrogenase [ *Aureobasidium* *melanogenum* CBS 110374] | 0 | 1.87 | 1.71 | 2.18 | 1.59 |
| A07195 | KEQ59361.1; hypothetical protein M437DRAFT_78179 [ *Aureobasidium* *melanogenum* CBS 110374] | 0 | 1.57 | 1.08 | 1.54 | 1.46 |
| A07224 | KEQ58878.1; glycoside hydrolase family 55 protein [ *Aureobasidium* *melanogenum* CBS 110374] | 0 | 1.77 | 2.7 | 2.78 | 2.68 |
| A07231 | KEQ58884.1; glycosyl hydrolase 53 [ *Aureobasidium* *melanogenum* CBS 110374] | 0 | 1.05 | 1.19 | 1.36 | 1.02 |
| A07255 | KEQ59479.1; hypothetical protein M437DRAFT_69158 [ *Aureobasidium* *melanogenum* CBS 110374] | 0 | 1.31 | 0.96 | 1.14 | 0.97 |
| A07264 | XP_013427743.1; hypothetical protein M436DRAFT_81596 [ *Aureobasidium* *namibiae* CBS 147.97] | 0 | 2.07 | 2.29 | 2.86 | 3.04 |
| A07272 | KEQ59496.1; cytochrome P450 alkane hydroxylase-like protein [ *Aureobasidium* *melanogenum* CBS 110374] | 0 | 1.57 | 1.24 | 1.87 | 1.74 |
| A07285 | NA | 0 | 2.82 | 1.82 | 1.54 | 3.13 |
| A07303 | NA | 0 | 2.53 | 1.95 | 2.5 | 2.51 |
| A07315 | XP_013425795.1; DUF1917-domain-containing protein [ *Aureobasidium* *namibiae* CBS 147.97] | 0 | 2.07 | 2.11 | 2.65 | 2.45 |
| A07342 | KEQ67731.1; MFS general substrate transporter [ *Aureobasidium* *melanogenum* CBS 110374] | 0 | 2.63 | 2.8 | 3.76 | 3.89 |
| A07343 | KEQ67730.1; actin-like ATPase domain-containing protein [ *Aureobasidium* *melanogenum* CBS 110374] | 0 | 2.25 | 3.09 | 2.39 | 2.32 |
| A07363 | KEQ67825.1; hypothetical protein M437DRAFT_37255, partial [ *Aureobasidium* *melanogenum* CBS 110374] | 0 | 1.54 | 1.49 | 1.79 | 2.12 |
| A07368 | KEQ89815.1; peptidase C1B, bleomycin hydrolase [ *Aureobasidium* *pullulans* EXF-150] | 0 | 4.32 | 2.65 | 3.26 | 2.89 |
| A07387 | KEQ67802.1; hypothetical protein M437DRAFT_37677 [ *Aureobasidium* *melanogenum* CBS 110374] | 0 | 2 | 3.03 | 2.5 | 2.97 |
| A07399 | KEQ67929.1; hypothetical protein M437DRAFT_80556 [ *Aureobasidium* *melanogenum* CBS 110374] | 0 | 3.23 | 2.6 | 2.16 | 2.87 |
| A07412 | KEQ67941.1; Acetamidase/Formamidase [ *Aureobasidium* *melanogenum* CBS 110374] | 0 | 3.22 | 1.95 | 2.53 | 2.71 |
| A07459 | KEQ58832.1; Arabinanase/levansucrase/invertase [ *Aureobasidium* *melanogenum* CBS 110374] | 0 | 2.22 | 1.81 | 2.12 | 1.43 |
| A07483 | OQO29666.1; hypothetical protein B0A51_02571 [Rachicladosporium sp. CCFEE 5018] | 0 | 1.42 | 1.38 | 1.42 | 1.83 |
| A07500 | KEQ68027.1; hypothetical protein M437DRAFT_36781 [ *Aureobasidium* *melanogenum* CBS 110374] | 0 | 3.36 | 4.02 | 3.8 | 3.58 |
| A07501 | KEQ68028.1; putative beta-glucosidase [ *Aureobasidium* *melanogenum* CBS 110374] | 0 | 2.56 | 2.74 | 3.07 | 3.04 |
| A07516 | XP_013348158.1; hypothetical protein AUEXF2481DRAFT_35598 [ *Aureobasidium* *subglaciale* EXF-2481] | 0 | 1.05 | 0.87 | 1.21 | 1.16 |
| A07524 | NA | 0 | 2.34 | 2.86 | 2.26 | 1.71 |
| A07525 | XP_013347940.1; hypothetical protein AUEXF2481DRAFT_1334 [ *Aureobasidium* *subglaciale* EXF-2481] | 0 | 1.41 | 2.3 | 2.43 | 2.25 |
| A07532 | XP_013348277.1; hypothetical protein AUEXF2481DRAFT_61752 [ *Aureobasidium* *subglaciale* EXF-2481] | 0 | 2.16 | 1.91 | 2.17 | 2.01 |
| A07567 | KEQ67757.1; hypothetical protein M437DRAFT_36723 [ *Aureobasidium* *melanogenum* CBS 110374] | 0 | 1.1 | 1.26 | 1.27 | 0.87 |
| A07593 | KEQ78369.1; MFS general substrate transporter [ *Aureobasidium* *pullulans* EXF-150] | 0 | 2.55 | 3.66 | 2.68 | 3.47 |
| A07600 | XP_013344119.1; hypothetical protein AUEXF2481DRAFT_29018 [ *Aureobasidium* *subglaciale* EXF-2481] | 0 | 3.04 | 4.07 | 4.04 | 3.15 |
| A07613 | NA | 0 | 2.85 | 3.08 | 3.72 | 4.01 |
| A07618 | KEQ67680.1; beta-lactamase/transpeptidase-like protein [ *Aureobasidium* *melanogenum* CBS 110374] | 0 | 3.75 | 4.91 | 3.67 | 4.61 |
| A07669 | XP_003040753.1; hypothetical protein NECHADRAFT_67931 [Nectria haematococca mpVI 77-13-4] | 0 | 1.26 | 1.99 | 1.58 | 2.01 |
| A07670 | KEQ58834.1; flavo protein [ *Aureobasidium* *melanogenum* CBS 110374] | 0 | 0.85 | 0.81 | 1.08 | 1.23 |
| A07682 | XP_013342252.1; hypothetical protein AUEXF2481DRAFT_30790 [ *Aureobasidium* *subglaciale* EXF-2481] | 0 | 1.44 | 1.29 | 1.32 | 1.26 |
| A07690 | KEQ67857.1; hypothetical protein M437DRAFT_38543 [ *Aureobasidium* *melanogenum* CBS 110374] | 0 | 1.51 | 1.15 | 1.66 | 1.8 |
| A07714 | KEQ58916.1; hypothetical protein M437DRAFT_78668 [ *Aureobasidium* *melanogenum* CBS 110374] | 0 | 1.51 | 2 | 2 | 1.85 |
| A07738 | XP_013431513.1; hypothetical protein M436DRAFT_17923, partial [ *Aureobasidium* *namibiae* CBS 147.97] | 0 | 2.32 | 2.89 | 2.38 | 3.14 |
| A07743 | KEQ67914.1; concanavalin A-like lectin/glucanase [ *Aureobasidium* *melanogenum* CBS 110374] | 0 | 3.32 | 3.5 | 3.57 | 3.45 |
| A07756 | KEQ67793.1; beta ketoadipate:succinyl-CoA transferase, TR1 [ *Aureobasidium* *melanogenum* CBS 110374] | 0 | 2.31 | 2.3 | 2.83 | 3.12 |
| A07774 | NA | 0 | 2.25 | 3.1 | 3.15 | 3.1 |
| A07795 | KEQ67630.1; GroES-like protein [ *Aureobasidium* *melanogenum* CBS 110374] | 0 | 2.2 | 2.09 | 2.22 | 3.07 |
| A07808 | KEQ67618.1; RNA polymerase I-specific transcription initiation factor RRN3 [ *Aureobasidium* *melanogenum* CBS 110374] | 0 | 2.7 | 3.51 | 3.53 | 2.95 |
| A07831 | KEQ81480.1; glutathione S-transferase [ *Aureobasidium* *pullulans* EXF-150] | 0 | 1.57 | 1.59 | 1.02 | 0.86 |
| A07847 | KEQ58899.1; yippee-domain-containing protein [ *Aureobasidium* *melanogenum* CBS 110374] | 0 | 1.25 | 1.43 | 2.08 | 1.92 |
| A07848 | KEQ67581.1; hypothetical protein M437DRAFT_61975 [ *Aureobasidium* *melanogenum* CBS 110374] | 0 | 2.26 | 3.62 | 3.87 | 3.97 |
| A07849 | XP_013432386.1; cation efflux protein [ *Aureobasidium* *namibiae* CBS 147.97] | 0 | 1.28 | 1.16 | 1.14 | 0.92 |
| A07873 | KEQ67556.1; nascent polypeptide-associated complexase [ *Aureobasidium* *melanogenum* CBS 110374] | 0 | 0.88 | 1.06 | 1.25 | 0.95 |
| A07875 | KEQ67553.1; hypothetical protein M437DRAFT_80197 [ *Aureobasidium* *melanogenum* CBS 110374] | 0 | 0.76 | 1.04 | 0.65 | 1.12 |
| A07884 | KEQ67545.1; hypothetical protein M437DRAFT_36047 [ *Aureobasidium* *melanogenum* CBS 110374] | 0 | 1.59 | 1.48 | 1.57 | 1.27 |
| A07968 | KEQ67274.1; hypothetical protein M437DRAFT_61697 [ *Aureobasidium* *melanogenum* CBS 110374] | 0 | 1.04 | 0.82 | 0.93 | 0.79 |
| A07973 | KEQ67278.1; hypothetical protein M437DRAFT_37481 [ *Aureobasidium* *melanogenum* CBS 110374] | 0 | 2.41 | 3.04 | 3.1 | 3.3 |
| A07975 | KEQ67279.1; hypothetical protein M437DRAFT_22760, partial [ *Aureobasidium* *melanogenum* CBS 110374] | 0 | 2.31 | 1.64 | 1.99 | 2.3 |
| A07978 | XP_013432366.1; catalase [ *Aureobasidium* *namibiae* CBS 147.97] | 0 | 1.71 | 1.69 | 1.84 | 1.52 |
| A07984 | KEQ67287.1; hypothetical protein M437DRAFT_37871 [ *Aureobasidium* *melanogenum* CBS 110374] | 0 | 0.92 | 1.23 | 1.16 | 1.43 |
| A08049 | KEQ67348.1; PLP-dependent transferase [ *Aureobasidium* *melanogenum* CBS 110374] | 0 | 4.41 | 5.06 | 5.11 | 3.37 |
| A08091 | NA | 0 | 1.99 | 3.45 | 2.94 | 3.79 |
| A08092 | KEQ67392.1; hypothetical protein M437DRAFT_26894, partial [ *Aureobasidium* *melanogenum* CBS 110374] | 0 | 2.95 | 2.6 | 2.93 | 2.77 |
| A08098 | KEQ67397.1; Pentulose kinase [ *Aureobasidium* *melanogenum* CBS 110374] | 0 | 1.67 | 1.89 | 2.14 | 1.87 |
| A08127 | KEQ62052.1; Cloroperoxidase [ *Aureobasidium* *melanogenum* CBS 110374] | 0 | 2.16 | 2.61 | 1.79 | 3.08 |
| A08128 | KEQ61271.1; cytochrome P450 oxidoreductase [ *Aureobasidium* *melanogenum* CBS 110374] | 0 | 4.56 | 4.78 | 5.05 | 5.27 |
| A08147 | KEQ60451.1; ubiquitin carboxyl-terminal hydrolase [ *Aureobasidium* *melanogenum* CBS 110374] | 0 | 1.71 | 1.85 | 2.6 | 2.25 |
| A08169 | KEQ60482.1; hypothetical protein M437DRAFT_54327 [ *Aureobasidium* *melanogenum* CBS 110374] | 0 | 1.42 | 1.04 | 1.39 | 1.27 |
| A08174 | KEQ60487.1; hypothetical protein M437DRAFT_86580 [ *Aureobasidium* *melanogenum* CBS 110374] | 0 | 1.12 | 0.92 | 0.82 | 0.63 |
| A08182 | KEQ60471.1; amino acid transporter [ *Aureobasidium* *melanogenum* CBS 110374] | 0 | 1.8 | 0.76 | 0.89 | 1.08 |
| A08185 | KEQ60496.1; acyl-CoA dehydrogenase [ *Aureobasidium* *melanogenum* CBS 110374] | 0 | 1.83 | 3.34 | 2.68 | 3.16 |
| A08193 | KEQ60504.1; alpha/beta hydrolase fold protein [ *Aureobasidium* *melanogenum* CBS 110374] | 0 | 2.95 | 1.78 | 2.99 | 2.15 |
| A08217 | KEQ60531.1; N-carbamoyl-L-amino acid hydrolase [ *Aureobasidium* *melanogenum* CBS 110374] | 0 | 3.9 | 2.03 | 2.26 | 2.41 |
| A08221 | NA | 0 | 3.73 | 3.61 | 3.25 | 4.04 |
| A08255 | KEQ60576.1; ClpP/crotonase [ *Aureobasidium* *melanogenum* CBS 110374] | 0 | 3.6 | 2.97 | 2.78 | 3.15 |
| A08272 | KEQ60594.1; hypothetical protein M437DRAFT_68130 [ *Aureobasidium* *melanogenum* CBS 110374] | 0 | 1.61 | 1.54 | 1.23 | 1.4 |
| A08289 | KEQ60612.1; DUF125-domain-containing protein [ *Aureobasidium* *melanogenum* CBS 110374] | 0 | 2.58 | 2.25 | 2.87 | 2.5 |
| A08299 | KEQ60621.1; Arginase/deacetylase [ *Aureobasidium* *melanogenum* CBS 110374] | 0 | 9.63 | 8.26 | 8.27 | 8.16 |
| A08306 | KEQ62994.1; nitrite reductase [ *Aureobasidium* *melanogenum* CBS 110374] | 0 | 3.37 | 3.38 | 3.59 | 3.3 |
| A08307 | KEQ62993.1; nitrate reductase [ *Aureobasidium* *melanogenum* CBS 110374] | 0 | 2.55 | 2.95 | 2.95 | 2.62 |
| A08352 | KEQ63106.1; hypothetical protein M437DRAFT_84145 [ *Aureobasidium* *melanogenum* CBS 110374] | 0 | 3.29 | 1.84 | 2.35 | 1.61 |
| A08360 | KEQ63113.1; hypothetical protein M437DRAFT_75009 [ *Aureobasidium* *melanogenum* CBS 110374] | 0 | 1.78 | 1.61 | 1.32 | 1.52 |
| A08372 | KEQ83688.1; hypothetical protein M438DRAFT_366348 [ *Aureobasidium* *pullulans* EXF-150] | 0 | 1.62 | 1.6 | 1.84 | 1.88 |
| A08445 | KEQ63128.1; hypothetical protein M437DRAFT_47817 [ *Aureobasidium* *melanogenum* CBS 110374] | 0 | 2.25 | 3.2 | 2.88 | 2.33 |
| A08450 | KEQ63133.1; hypothetical protein M437DRAFT_14554, partial [ *Aureobasidium* *melanogenum* CBS 110374] | 0 | 0.9 | 1.08 | 1.03 | 0.97 |
| A08458 | KEQ63141.1; general substrate transporter [ *Aureobasidium* *melanogenum* CBS 110374] | 0 | 1.38 | 1.84 | 1.78 | 1.89 |
| A08473 | KEQ63154.1; Bud-site selection protein [ *Aureobasidium* *melanogenum* CBS 110374] | 0 | 1.42 | 2.02 | 1.47 | 1.64 |
| A08477 | KEQ63158.1; hypothetical protein M437DRAFT_48006 [ *Aureobasidium* *melanogenum* CBS 110374] | 0 | 1.95 | 2.86 | 3.17 | 2.63 |
| A08481 | KEQ63161.1; siroheme synthase [ *Aureobasidium* *melanogenum* CBS 110374] | 0 | 2.14 | 2.6 | 3.18 | 3.41 |
| A08492 | KEQ63173.1; ATP-dependent rRNA helicase SPB4 [ *Aureobasidium* *melanogenum* CBS 110374] | 0 | 1.28 | 2.09 | 2.46 | 2.22 |
| A08504 | XP_013346264.1; hypothetical protein AUEXF2481DRAFT_27241 [ *Aureobasidium* *subglaciale* EXF-2481] | 0 | 1.33 | 1.91 | 1.97 | 1.95 |
| A08508 | KEQ63193.1; purine-cytosine permease [ *Aureobasidium* *melanogenum* CBS 110374] | 0 | 1.98 | 2.76 | 2.52 | 2.43 |
| A08575 | XP_013341604.1; hypothetical protein AUEXF2481DRAFT_42443 [ *Aureobasidium* *subglaciale* EXF-2481] | 0 | 1.07 | 1.04 | 1.44 | 1.19 |
| A08638 | XP_016211752.1; hypothetical protein PV09_06730 [Verruconis gallopava] | 0 | 3.3 | 3.74 | 4.01 | 4.26 |
| A08647 | KEQ79817.1; OPT-domain-containing protein [ *Aureobasidium* *pullulans* EXF-150] | 0 | 1.41 | 1.24 | 1.34 | 1.67 |
| A08658 | OBW64644.1; hypothetical protein AUREO_052870 [ *Aureobasidium* *pullulans*] | 0 | 2.5 | 2.46 | 2.06 | 1.48 |
| A08680 | KEQ61122.1; hypothetical protein M437DRAFT_67501 [ *Aureobasidium* *melanogenum* CBS 110374] | 0 | 1.49 | 1.49 | 1.38 | 0.8 |
| A08683 | OBW67502.1; hypothetical protein AUREO_024180 [ *Aureobasidium* *pullulans*] | 0 | 1.65 | 3.07 | 2.67 | 2.77 |
| A08689 | KEQ61132.1; A1pp-domain-containing protein [ *Aureobasidium* *melanogenum* CBS 110374] | 0 | 2.64 | 3.86 | 3.29 | 2.69 |
| A08697 | KEQ61141.1; hypothetical protein M437DRAFT_76709 [ *Aureobasidium* *melanogenum* CBS 110374] | 0 | 1.68 | 1.58 | 1.27 | 1.36 |
| A08702 | KEQ61239.1; MFS general substrate transporter [ *Aureobasidium* *melanogenum* CBS 110374] | 0 | 1.02 | 0.69 | 1 | 1.28 |
| A08711 | OBW69832.1; Amidohydrolase family protein [ *Aureobasidium* *pullulans*] | 0 | 1.13 | 1.25 | 0.99 | 0.69 |
| A08722 | KEQ61076.1; hypothetical protein M437DRAFT_67463 [ *Aureobasidium* *melanogenum* CBS 110374] | 0 | 1.03 | 0.56 | 0.7 | 0.86 |
| A08725 | KEQ61079.1; hypothetical protein M437DRAFT_52614 [ *Aureobasidium* *melanogenum* CBS 110374] | 0 | 3.48 | 5.15 | 4.6 | 5.28 |
| A08726 | KEQ61080.1; hypothetical protein M437DRAFT_52550 [ *Aureobasidium* *melanogenum* CBS 110374] | 0 | 5.69 | 7.94 | 8.34 | 9.3 |
| A08754 | KEQ61212.1; hypothetical protein M437DRAFT_52723 [ *Aureobasidium* *melanogenum* CBS 110374] | 0 | 1.38 | 0.77 | 1.08 | 0.66 |
| A08759 | KEQ61206.1; Metallo-dependent phosphatase [ *Aureobasidium* *melanogenum* CBS 110374] | 0 | 1.51 | 2.6 | 2.6 | 2.51 |
| A08771 | KEQ61192.1; hypothetical protein M437DRAFT_67567 [ *Aureobasidium* *melanogenum* CBS 110374] | 0 | 1.37 | 1.39 | 1.52 | 0.91 |
| A08797 | KEQ61227.1; glycoside hydrolase family 31 protein [ *Aureobasidium* *melanogenum* CBS 110374] | 0 | 3.01 | 3.4 | 3.61 | 3.18 |
| A08826 | KEQ61240.1; cytochrome P450 [ *Aureobasidium* *melanogenum* CBS 110374] | 0 | 1 | 1.49 | 1.33 | 1.36 |
| A08866 | KEQ58150.1; NAD(P)-binding protein [ *Aureobasidium* *melanogenum* CBS 110374] | 0 | 2.09 | 3.1 | 3.31 | 2.76 |
| A08890 | KEQ58231.1; hypothetical protein M437DRAFT_59858 [ *Aureobasidium* *melanogenum* CBS 110374] | 0 | 1.48 | 1.71 | 2.27 | 2.04 |
| A08900 | NA | 0 | 1.25 | 1.23 | 1.11 | 1.75 |
| A08920 | XP_013331645.1; MFS transporter [Rasamsonia emersonii CBS 393.64] | 0 | 1.02 | 1.28 | 1.46 | 1.45 |
| A08956 | KEQ59053.1; hypothetical protein M437DRAFT_58037 [ *Aureobasidium* *melanogenum* CBS 110374] | 0 | 1.52 | 1.8 | 1.69 | 1.27 |
| A08978 | KEQ58180.1; DEAD/DEAH box helicase-like protein [ *Aureobasidium* *melanogenum* CBS 110374] | 0 | 0.94 | 0.65 | 0.57 | 1.07 |
| A08985 | KEQ58188.1; hypothetical protein M437DRAFT_88797 [ *Aureobasidium* *melanogenum* CBS 110374] | 0 | 2.1 | 2.02 | 1.74 | 1.63 |
| A08988 | KEQ58191.1; glycoside hydrolase family 62 protein [ *Aureobasidium* *melanogenum* CBS 110374] | 0 | 1.24 | 1.6 | 1.25 | 1.4 |
| A08996 | KEQ59103.1; putative P450 monooxygenase [ *Aureobasidium* *melanogenum* CBS 110374] | 0 | 2.22 | 2.06 | 2.39 | 3.14 |
| A08997 | KEQ59080.1; NAD(P)-binding protein [ *Aureobasidium* *melanogenum* CBS 110374] | 0 | 4.7 | 4.17 | 4.71 | 5.37 |
| A08998 | KEQ59081.1; amino acid permease [ *Aureobasidium* *melanogenum* CBS 110374] | 0 | 2.91 | 3.85 | 2.35 | 3.6 |
| A08999 | KEQ59082.1; YjgF-like protein [ *Aureobasidium* *melanogenum* CBS 110374] | 0 | 1.87 | 2.25 | 2.07 | 2.62 |

*: The expression levels at 6 h are set as 100%. Data results are the average of three biological replicates.
